# Clone copy number diversity is linked to survival in lung cancer

**DOI:** 10.1038/s41586-025-09398-w

**Published:** 2025-08-13

**Authors:** Piotr Pawlik, Kristiana Grigoriadis, Abigail Bunkum, Helena Coggan, Alexander M. Frankell, Carlos Martinez-Ruiz, Takahiro Karasaki, Ariana Huebner, Andrew Rowan, Jasmin Fisher, Allan Hackshaw, Charles Swanton, Simone Zaccaria, Nicholas McGranahan

**Affiliations:** 1 Cancer Research UK Lung Cancer Centre of Excellence, University College London Cancer Institute, London, UK; 2 Cancer Genome Evolution Research Group, University College London Cancer Institute, London, UK; 3 Cancer Evolution and Genome Instability Laboratory, The Francis Crick Institute, London, UK; 4 Computational Cancer Genomics Research Group, University College London Cancer Institute, London, UK; 5 Cancer Metastasis Laboratory, University College London Cancer Institute, London, UK; 6 Computational Cancer Biology Research Group, University College London Cancer Institute, London, UK; 7 Department of Mathematics, University College London, London, UK; 8 Somatic Evolution Monitoring Laboratory, Early Cancer Institute, University of Cambridge, Cambridge, UK; 9 Department of Thoracic Surgery, Graduate School of Medicine, The University of Tokyo, Tokyo, Japan; 10 Cancer Research UK & UCL Cancer Trials Centre, London, UK; 11 Department of Oncology, University College London Hospitals, London, UK

**Keywords:** Non-small-cell lung cancer, Phylogeny, Computational models

## Abstract

Both single nucleotide variants (SNVs) and somatic copy number alterations (SCNAs) accumulate in cancer cells during tumour development, fuelling clonal evolution. However, accurate estimation of clone-specific copy numbers from bulk DNA-sequencing data is challenging. Here we present allele-specific phylogenetic analysis of copy number alterations (ALPACA), a method to infer SNV and SCNA coevolution by leveraging phylogenetic trees reconstructed from multi-sample bulk tumour sequencing data using SNV frequencies. ALPACA estimates the SCNA evolution of simulated tumours with a higher accuracy than current state-of-the-art methods^1–4^. ALPACA uncovers loss-of-heterozygosity and amplification events in minor clones that may be missed using standard approaches and reveals the temporal order of somatic alterations. Analysing clone-specific copy numbers in TRACERx421 lung tumours^5,6^, we find evidence of increased chromosomal instability in metastasis-seeding clones and enrichment for losses affecting tumour suppressor genes and amplification affecting *CCND1*. Furthermore, we identify increased SCNA rates in both tumours with polyclonal metastatic dissemination and tumours with extrathoracic metastases, and an association between higher clone copy number diversity and reduced disease-free survival in patients with lung cancer.

## Abstract (web summary)

A study presents ALPACA, a computational method for inferring clone- and allele-specific copy numbers of individual clones from multi-sample bulk DNA-sequencing data, and demonstrates its use to study metastasis trajectories.

## Main

A key driver of cancer development and progression is genomic instability. This includes mutational processes and chromosomal instability (CIN), resulting in SNVs and SCNAs, respectively. Multiple genomic instability measures, based on either SNVs or SCNAs, have been linked to poor prognosis^7–12^. The somatic alterations caused by genomic instability may be subject to positive selection through clonal evolution^7,13^, resulting in genetically heterogeneous, phylogenetically related, cell populations.

However, most work exploring cancer evolution, genomic instability and tumour diversity has considered SNVs and SCNAs separately^2,3,14–19^. As such, the underlying genomic features of aggressive tumour clones and the interplay between SNV and SCNA diversity remain unclear. Obtaining reliable clone-specific SNVs and SCNAs simultaneously is required to obtain a deeper understanding of genome instability processes and how they contribute to metastasis and poor prognosis.

To address this, we developed ALPACA, a method for inference of allele-specific copy numbers of individual clones from multiple bulk DNA-sequencing samples. Here we show that ALPACA accurately infers clone-specific copy numbers and outperforms other approaches. We apply ALPACA to a large cohort of non-small cell lung cancers (NSCLCs), from the recent longitudinal, prospective TRACERx421 study^5,6^.

## ALPACA algorithm

**Extended Data Fig. 1:**
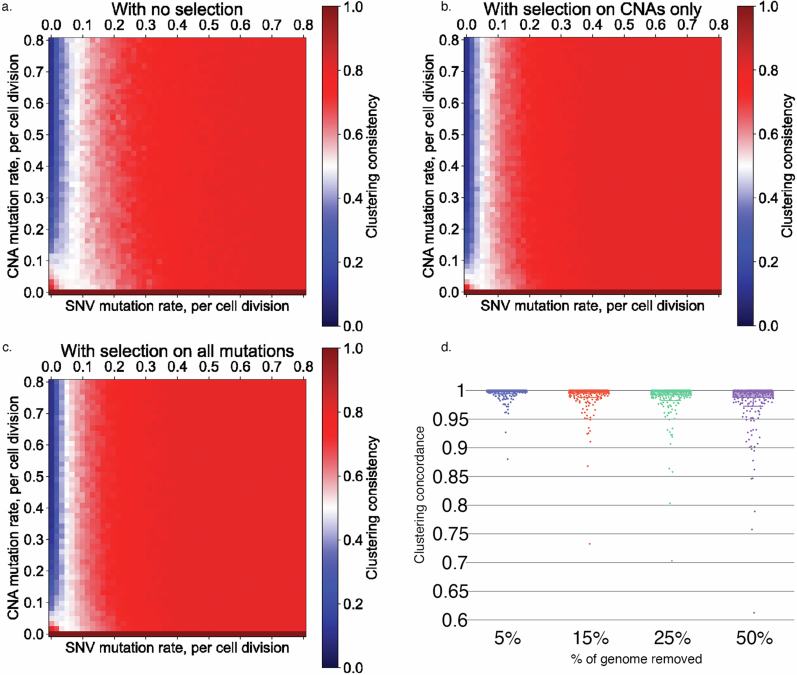
ALPACA assumptions. **a-c**, Variation of clustering consistency with varied SNV and CNA mutation rates, (a) assuming neutral growth, (b) with selection on CNAs only, (c) and with selection on both CNAs and SNVs. For all pairs of mutation rates, consistency values are averaged across 10 simulations. **d**, Clustering concordance of mutations from TRACERx primary cohort cases between the full set of mutations and subsets. Each box plot represents one subset (n = 310), where 5%, 15%, 25%, or 50% of the genome with extensive SCNA has been removed.

ALPACA leverages the clonal structure and tumour phylogeny derived from SNVs as a scaffold to guide inference of SCNA evolution (Methods and Supplementary Information). Thus, ALPACA assumes that the SNV mutation rate is sufficiently high to ensure that each clone harbours mutations that can act as a barcode to accurately estimate clone prevalence. Our modelling suggests that a rate of only about 0.3 SNVs per cell division is required to meet this assumption (Extended Data Fig. 1a–c and Supplementary Information), and thereby many cancer types, including renal and colorectal cancers^20^, have sufficient mutations using whole-exome sequencing (WES) to fulfil this assumption. Moreover, we extensively validated the suitability of the SNV-derived phylogenetic trees for subsequent SCNA reconstruction (Extended Data Fig. 1d and Supplementary Information).

**Fig. 1:**
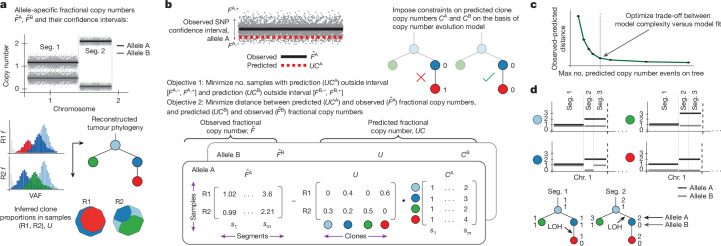
Overview of ALPACA algorithm. **a**, Input: observed fractional copy numbers $\hat{F}$ (where *F* denotes the average copy number of all cancer cells in the sample) for each genomic segment (Seg.), tumour phylogeny and clone proportions, $U$. The fraction of the octagonal cloneMap^44^ covered by each colour represents the clone proportion in that sample. VAF, variant allele frequency. **b**, The model aims to infer integer clone copy numbers, $C,$ by optimizing two objectives: minimizing the number of samples with a predicted fractional copy number outside the confidence intervals of $\hat{F}$; and minimizing the distance between the predicted and observed fractional copy numbers. The linear optimization is subject to evolutionary constraints, including persistent LOH on each tree path. **c**, The optimal solution is selected on the basis of a model selection procedure. **d**, ALPACA outputs clone- and allele-specific integer copy-number profiles for each genomic segment.

ALPACA requires three inputs (Fig. 1a): a tumour clone tree, clone proportions in each sample and allele-specific fractional copy number estimates. These inputs can be obtained by existing methods for tumour phylogenetic analysis^21–27^ and SCNA analysis^28–30^. ALPACA infers the clone-specific copy numbers using a mixed-integer linear programming and model selection approach, adopting an evolutionary model similarly to previous studies^4,31^ (for example, persistence of loss-of-heterozygosity (LOH) events; Fig. 1b,c). As a result, ALPACA outputs a tumour evolutionary history incorporating both SNVs and SCNAs (Fig. 1d).

**Extended Data Fig. 2:**
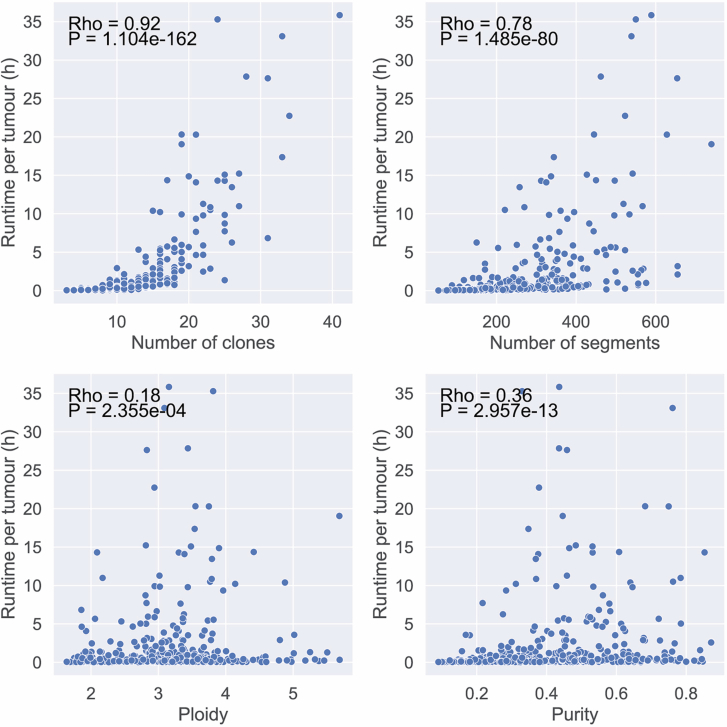
CPU runtime per tumour in TRACERx421 cohort. Scatter plots showing the total CPU runtime (in hours) in TRACERx421 cohort per tumour against number of clones (upper left), number of segments (upper right), ploidy (bottom left) and purity (bottom right). Spearman’s Rho and P values are annotated on each plot. Each point represents one tumour (n = 395).

In contrast to other methods^2,3,14,19^, ALPACA infers the copy numbers of extant tumour clones (that is, clones that are present in at least one sample) and also of extinct clones, enabling exploration of SCNA evolution and timing. Critically, ALPACA simplifies the complex copy-number deconvolution problem into an easier linear optimization problem, permitting analysis of large cohorts^5,6^ (Extended Data Fig. 2).

## ALPACA accurately infers clone copy number

**Fig. 2:**
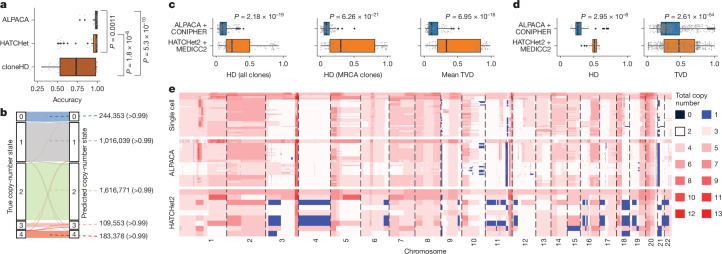
Benchmarking ALPACA. **a**, Accuracy comparison between ALPACA, HATCHet and cloneHD on MASCoTE simulations^2^ (*n* = 64 simulations). **b**, True (0–4, left) and predicted (0–4, right) copy numbers in simulated dataset. Values to the right of each flow represent the number and fraction of true copy numbers per clone- and allele-specific segment predicted correctly. **c**, Comparison of SCNA inference between CONIPHER + ALPACA and HATCHet2 + MEDICC2 (*n* = 150 simulations). **d**, Comparison of SCNA inference between CONIPHER + ALPACA and HATCHet2 + MEDICC2 applied to experimental WES dataset (*n* = 707 genomic segments). **e**, Heat maps showing reconstructed total copy-number profiles of the experimental dataset in Fig. 2d using single cell, ALPACA and HATCHet2. All tests in this figure are two-sided paired Wilcoxon. In data represented using box plots, the box represents the interquartile range (IQR) with the median line. Whiskers denote the lowest and highest values within 1.5 times the IQR from the first and third quartiles, respectively.

**Extended Data Fig. 3:**
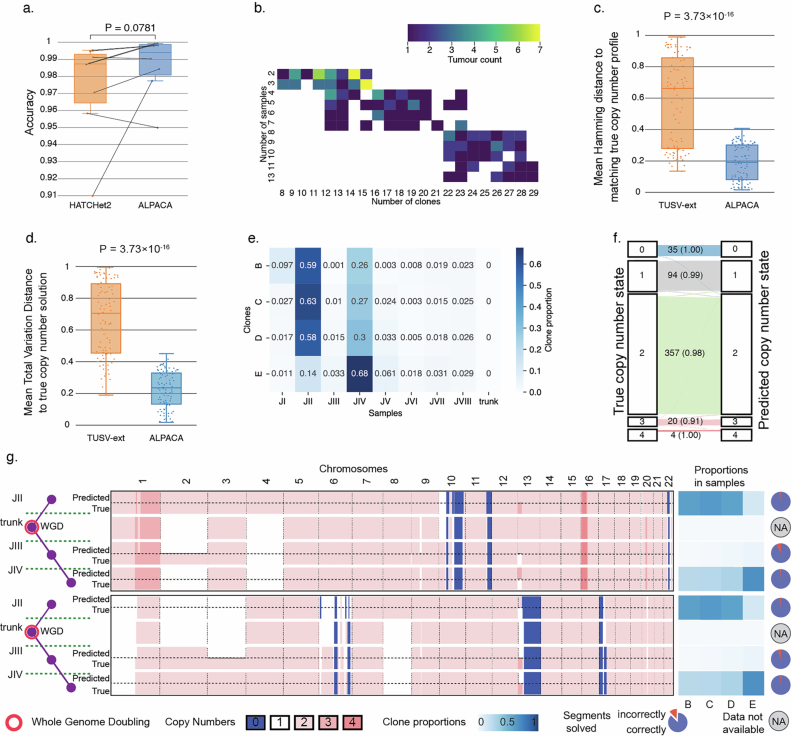
Benchmarking ALPACA. **a**, Accuracy comparison between ALPACA and HATCHet2 using MASCoTE simulations (n = 8 simulated tumours). Each data point represents one simulated tumour (Wilcoxon signed-rank test), **b**, Overview of the simulated cohort. **c**, Comparison of results between ALPACA and TUSV-ext applied to the simulated dataset (n = 88 simulated tumours) using total variation distance (Wilcoxon signed-rank test). **d**, Comparison of results between ALPACA and TUSV-ext applied to simulated dataset (n = 88 simulated tumours) using mean Hamming distance between true copy number profile of each clone and most similar (based on Hamming distance) copy number profile predicted by each model (Wilcoxon signed-rank test). **e**, Clone proportions in each sample of the analysed single cell breast tumour case. **f**, Sankey plot showing the predicted vs true copy number states in the single cell breast tumour experiment. Values in each flow represent the number and fraction of true copy number states (0–4, left) per clone- and allele-specific segment predicted correctly (0–5+ or 4, right). **g**, ALPACA output for single cell breast tumour case. Top: allele A, bottom: allele B. Each row represents one clone. The first column contains a phylogenetic tree. Heatmap in the middle shows the true and predicted copy number states. Each clone track is divided into sections: upper (showing predicted copy numbers) and lower (showing true copy numbers). True copy number states for the trunk were not available. Heatmap (right) represents clone proportions in each sample (B, C, D and E), pie charts represent the fraction of correctly (blue) and incorrectly (red) solved segments per clone. Since the true data for the trunk was not available, pie charts show ‘NA’ for this clone.

We benchmarked ALPACA against HATCHet^2^ and cloneHD^18^ using MASCoTE^2^ simulations; ALPACA significantly outperformed both methods (*P* = 5.3 × 10^−10^ and *P* = 0.0011, respectively, Wilcoxon signed-rank test; Fig. 2a). ALPACA also outperformed HATCHet2^3^ in six out of eight MASCoTE simulations in which this tool had been implemented (Extended Data Fig. 3a).

To assess the accuracy of ALPACA using more complex trees, we harnessed a published cohort of 150 simulated tumours^5,21^ (Tx simulations; Extended Data Fig. 3b).

**Extended Data Fig. 4:**
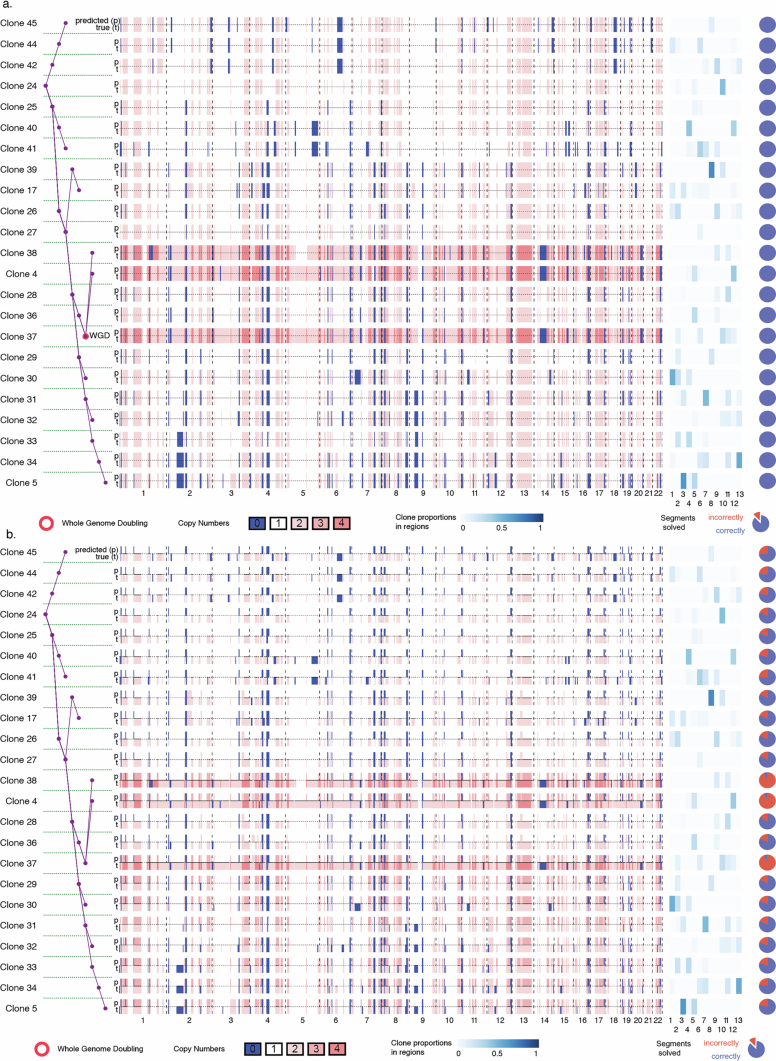
ALPACA vs simple model. **a**, ALPACA output for simulated case SIM127, allele A. Each row represents one clone. The first column contains a phylogenetic tree. Heatmap in the middle shows the true (top part of each track) and predicted (bottom part of each track) copy number states for each segment of each clone. Heatmap on the right hand side represents clone proportions in each sample, accompanied by pie charts representing the fraction of correctly (blue) and incorrectly (red) solved segments per clone. **b**, As in **a** but the solution was obtained with the simple model.

We first compared ALPACA with a published method that maps sample-specific SCNAs to clones of the inferred tree on the basis of multi-sample tumour data^5^ (Methods). ALPACA maintained high accuracy in complex tumours compared with this method (Extended Data Fig. 4). In more than 99% (3,170,094 of 3,179,926) of clone- and allele-specific segments, the copy-number state predicted by ALPACA was correct (Fig. 2b).

Next, given that ALPACA relies on accurate tree topology, we compared the joint performance of a phylogenetic reconstruction method (CONIPHER^21^) combined with ALPACA against that of clone copy-number inference using HATCHet2 (ref. ^3^) combined with copy-number phylogenetic reconstruction using MEDICC2 (ref. ^4^). As inferred phylogenies differed, we used two approaches to compare them: Hamming distance (HD)^32,33^ and total variation distance^3^ (TVD; Methods). The combination of CONIPHER and ALPACA obtained significantly better results compared to the combination of HATCHet2 and MEDICC2 (mean HD: 0.12 versus 0.35, *P* = 2.18 × 10^−19^; mean HD most recent common ancestor (MRCA) 0.10 versus 0.44, *P* = 6.26 × 10^−21^; mean TVD 0.16 versus 0.48, *P* = 6.95 × 10^−18^, Wilcoxon signed-rank test; Fig. 2c).

Additionally, for a subset of 88 simulations, we compared the results with TUSV-ext, a method designed to infer tumour evolutionary trees using SNVs, SCNAs and structural variants^1^. Our simulations did not contain structural variants, so we could not take full advantage of TUSV-ext. However, when using only SNVs and SCNAs, ALPACA obtained significantly better scores for both mean HD (0.19 versus 0.58, *P* = 3.73 × 10^−16^) and TVD (0.23 versus 0.67, *P* = 3.73 × 10^−16^, Wilcoxon signed-rank test; Extended Data Fig. 3c,d).

To compare ALPACA’s performance with that of HATCHet2 plus MEDICC2 using real tumour data, we leveraged a published single-cell multi-sample whole-genome sequencing dataset obtained from a lung squamous cell carcinoma tumour (LUSC)^34^. We applied CONIPHER + ALPACA and HATCHet2 + MEDICC2 to WES data from these samples and compared total copy-number profiles with the data obtained from SPRINTER^34^. ALPACA obtained significantly better results compared to HATCHet2 and MEDICC2 (mean HD of 0.24 versus 0.50, *P* = 2.95 × 10^−8^; mean TVD 0.28 versus 0.47, *P* = 2.61 × 10^−54^; Wilcoxon signed-rank test; Fig. 2d). Some discrepancies in WES-based inference might be explained by the fact that adjacent tumour samples were taken for WES and whole-genome sequencing experiments (Fig. 2e).

Finally, to further evaluate the performance of ALPACA, we harnessed published single-cell sequencing data from a breast tumour^35^. After exclusion of small clones (Methods and Extended Data Fig. 3e), we found that in more than 97% (510 of 522) of clone- and allele-specific segments, the copy-number state predicted by ALPACA was consistent with single-cell inference (Extended Data Fig. 3f,g).

## ALPACA identifies concealed SCNAs

Having established that ALPACA can reliably infer clone- and allele-specific copy numbers, we applied it to multi-sample tumour data from TRACERx421 (refs. ^5,6^). TRACERx421 comprises 421 prospectively recruited, untreated patients with NSCLC (predominantly lung adenocarcinoma (LUAD, *n* = 248) and LUSC, *n* = 138) subject to multi-sample WES. Of this cohort, 126 had metastatic disease (including lymph nodes at the time of primary surgery) that was sampled and sequenced^5,6^.

**Extended Data Fig. 5:**
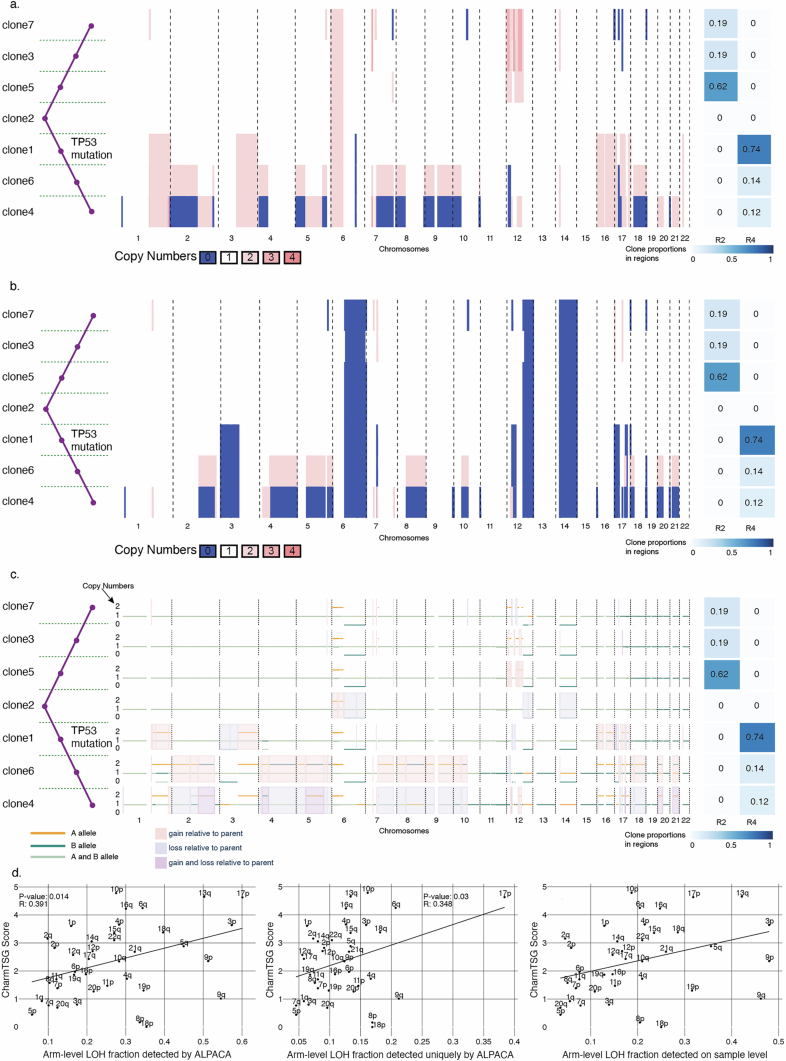
Example output. **a-b**, ALPACA solution for alleles A and B respectively for tumour case CRUK0628. Each row represents one clone. The first column contains a phylogenetic tree with TP53 driver mutation annotated in the clone where it occurred. Heatmap in the middle shows predicted copy number states for each segment of each clone. Heatmap on the right hand side represents clone proportions in each sample. **c**, Cross-genome plot for CRUK0628 case with tree (left hand side) and clone proportions heatmap (right hand side) as in **a** and **b**. Tracks in the middle show copy numbers for both alleles, highlighting the difference between child and parent clones. **d**, Left: correlation between fraction of arm level LOH called by ALPACA and CharmTSG scores (Pearson R = 0.391, P = 0.014). Centre: correlation between fraction of arm level LOH called by ALPACA, excluding LOH event called by sample-level analysis (i.e. where fractional copy-number was below 0.5) and CharmTSG scores (Pearson R = 0.348, P = 0.03). Right: correlation between fraction of arm level LOH called by sample-level analysis (i.e. where fractional copy-number was below 0.5) and CharmTSG scores (Pearson R = 0.354, P = 0.027).

A total of 395 primary tumours (Tx421-P cohort) and 126 paired primary–metastatic tumours (Tx421-PM cohort) had all of the required ALPACA inputs, resulting in 4,024 primary and 1,847 metastatic clone-level copy-number profiles. ALPACA plots for the primary LUAD tumour case with the Cancer Research UK identifier CRUK0628 with two samples and seven clones^6^ are shown in Extended Data Fig. 5a–c.

**Fig. 3:**
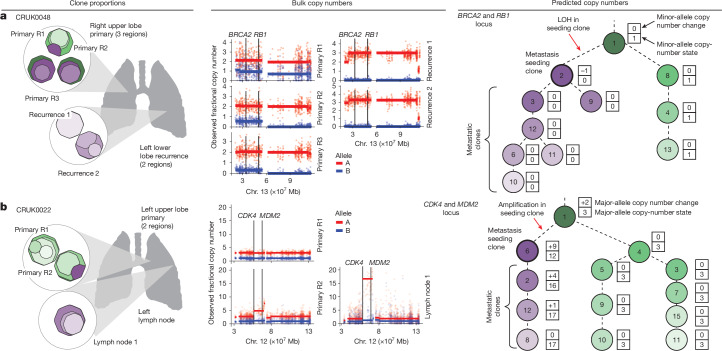
ALPACA provides additional clone-level SCNA resolution. **a**,**b**, ALPACA input and output for Tx421-PM cases CRUK0048 (**a**) and CRUK0022 (**b**). First two panels show input clone proportions in samples and bulk fractional copy numbers. The third panel shows the tumour phylogenetic tree annotated with ALPACA-predicted clone copy-number output. The green shaded clones are the primary-unique clones; the purple shaded clones are seeding and metastatic clones. The seeding clone node has a darker black outline on the tree. Mb, megabases.

The clone-specific resolution provided by ALPACA revealed new insights into the metastatic process. For instance, for CRUK0048, a LUAD tumour with 11 clones, ALPACA inferred LOH affecting 13q12.12–13q14.3 (encompassing *BRCA2*, *PDS5B* and *RB1*) to occur in primary tumour clone 2, before seeding a recurrence metastasis lesion (Fig. 3a). Notably, clone 2 was present at a low prevalence in all primary tumour samples; Primary R1 (clone prevalence = 22%), Primary R2 (clone prevalence = 18%) and Primary R3 (clone prevalence = 42%), and thus copy-number events occurring in this clone could be missed without ALPACA. More generally, the LOH calls made by ALPACA on the Tx421-P and Tx421-PM cohorts were corroborated by their correlation with published CharmTSG scores (Methods and Extended Data Fig. 5d).

ALPACA also provided increased resolution to identify subclonal amplifications. For example, in the metastatic LUAD tumour case CRUK0022 with 13 tumour clones^6^, the metastasis-seeding clone (clone 6) was present in the sample Primary R2 at clone prevalence of only about 21% and absent from the metastatic lymph node sample 1 (Fig. 3b). The 12q14.1–12q15 locus, harbouring *MDM2* and *CDK4*, exhibited a high copy number in the lymph node sample (fractional allele A copy number of about 16.64) and much lower values in the remaining primary samples (about 2.99 in Primary R1 and about 4.83 in Primary R2). ALPACA inferred that an amplification event had occurred in the seeding clone 6 in the primary tumour, before metastatic seeding (Fig. 3b). Notably, standard sample-level analysis would probably erroneously have inferred that the amplification event occurred following metastatic seeding, not before.

## Timing of events during NSCLC tumour evolution

**Fig. 4:**
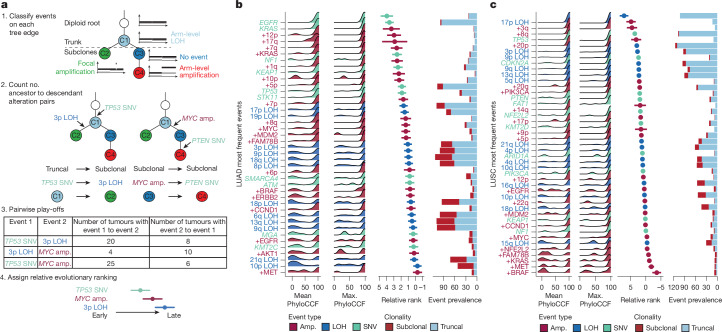
Timing of SNV and SCNA events in LUAD and LUSC. **a**, Method for timing somatic alterations using a Bradley–Terry model. amp., amplification. **b**,**c**, Bradley–Terry relative ranking estimate for the most frequent events in Tx421-P LUAD (*n* = 225; **b**) and LUSC (*n* = 126; **c**) cohorts. Plots include the following annotations: the distribution of the mean and maximum (max.) phylogenetic cancer cell fraction (PhyloCCF^21^) of the mutation clusters corresponding to the tree nodes that each event was assigned to, and the number of edges each event was assigned to, coloured by whether the edge was truncal or subclonal.

Exploiting ALPACA’s ability to assign SCNAs to cancer phylogenies, we investigated the relative evolutionary ordering of driver alterations in NSCLC (Fig. 4a). We quantified the frequency of ancestor–descendant relationships for selected alterations and used a Bradley–Terry model^36,37^ to infer their relative timing.

Arm-level amplifications typically preceded the focal amplifications in LUAD^38^. The proportion of subclonal arm-level LOH events was higher in LUAD than LUSC, resulting in these events being ordered relatively later in evolutionary time. In LUAD, the earliest events observed were driver SNVs in *EGFR*, *KRAS*, *NF1* and *KEAP1*, consistent with previous studies^36^, and arm-level amplifications affecting chromosome arms 12p, 17q, 7q and 1q (Fig. 4b). By contrast, in LUSC, the most prevalent arm-level amplifications and LOH events occurred predominantly in the MRCA clones (for example, chr. 17p LOH, which was never subclonal). Arm-level events typically preceded focal amplifications and many driver SNVs, with the exceptions of *TP53* SNV and *PIK3CA* amplification, which were almost always truncal (Fig. 4c).

**Extended Data Fig. 6:**
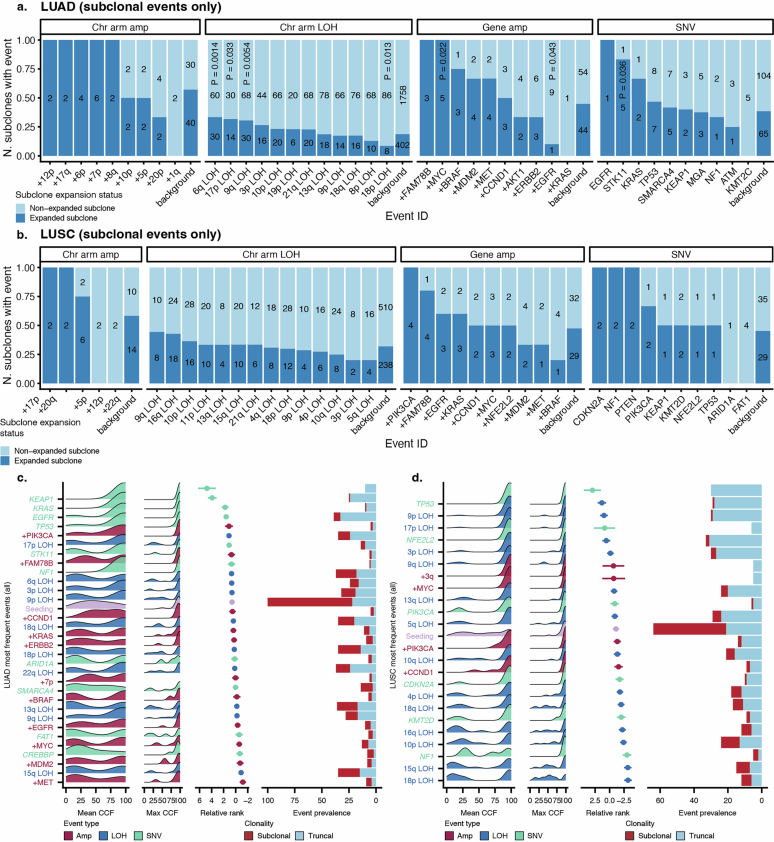
ALPACA enables evolutionary ordering of subclonal SNV and SCNA events in NSCLC. **a**, Comparing the proportions of subclonal occurrences of the top most frequent events from the Tx421-P cohort in expanded vs non-expanded subclones, compared with a background calculated as the total number of subclonal event occurrences of each event type (chromosome arm amplification, chromosome arm LOH, gene amplification and driver SNV) in expanded vs non-expanded subclones. Significant P values are annotated on the plot (Fisher’s exact test). **b, c** Pairwise event ordering and Bradley-Terry relative ranking estimate for the most frequent events in Tx421-PM **b**), **d**) LUAD (n = 65), and **c**), **e**) LUSC (n = 38) cohorts. The top most frequent arm-level SCNAs, focal SCNAs, driver SNVs and metastatic seeding are included. Plots include the following annotations: the distribution of the mean and maximum phylogenetic cancer cell fraction (PhyloCCF^21^) of the mutation clusters corresponding to the tree nodes that each event was assigned to, and the number of edges each event was assigned to, coloured by whether the edge was truncal or subclonal.

Next we investigated the relative size of the clones with the selected alterations. Subclonal amplifications affecting *MYC* in LUAD were enriched in expanded subclones^5^ (Methods), compared to a background of all oncogene amplifications (Fisher’s exact test: *P* = 0.022; Extended Data Fig. 6a), potentially indicating that these events are under stronger selection. Similarly, subclonal LOH of chromosome arms 17p (containing *TP53*), 6q (containing *LATS1*) and 9q (containing *NOTCH1*) were enriched in expanded clones, compared with a background of all chromosome-arm LOH events (Fisher’s exact text, *P* = 0.0326, *P* = 0.001 and *P* = 0.005, respectively). A similar analysis of these loci on the basis of single-sample analysis would erroneously assign these events to be truncal, and therefore earlier in tumour evolution. Further, multi-sample analysis without ALPACA would not be able to estimate the clonal fraction of the amplifications in different tumour samples. Notably, *EGFR* amplifications in LUAD were observed to be enriched in minor subclones that did not expand in the primary tumour (Fisher’s exact test, *P* = 0.043; Extended Data Fig. 6a), suggesting either that these frequently occur late in tumour development or that subclonal *EGFR* amplifications do not always confer a sufficient selective advantage to engender a large clonal expansion.

We further investigated the timing of the most frequent driver alterations relative to metastatic seeding. Similar relative timings were observed between the matched primary–metastatic phylogenies (Extended Data Fig. 6c,d) and the primary-alone phylogenies (Fig. 4). Notably, although *CCND1* amplification in LUAD was estimated to occur relatively late in primary tumour evolution, it nevertheless occurred frequently before metastatic seeding consistent with a potential role in the metastatic transition.

## SCNA patterns in metastasis

**Fig. 5:**
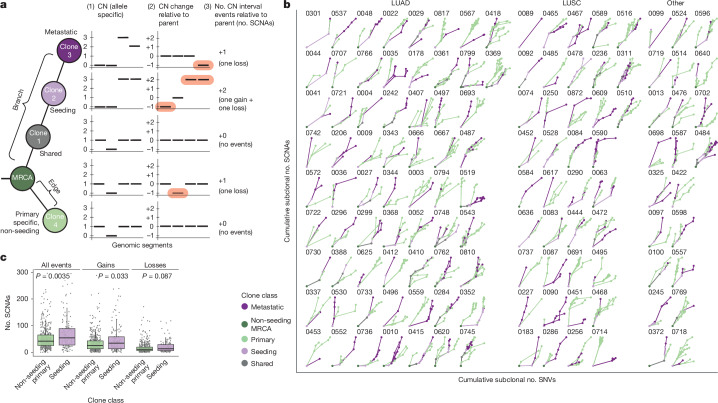
SCNA patterns in metastasis. **a**, Schematic example of a phylogenetic tree showing clone classes accompanied by (1) single-allele ALPACA output for four genomic segments, (2) segment-specific copy number changes on each edge and (3) aggregation of segment-specific copy number changes to interval events per edge. CN, copy number. **b**, Cumulative number of SNVs on each edge versus the cumulative number of SCNAs for each phylogenetic tree in the Tx421-PM cohort^6^. Numbers above trees represent the Cancer Research UK identifier. Patients are split by histology and branches are coloured on the basis of the clone classifications. **c**, Box plot comparing the number of SCNAs in seeding (*n* = 121) versus non-seeding (*n* = 434) primary clones (two-sided Wilcoxon test). The box represents the interquartile range (IQR) with the median line. Whiskers denote the lowest and highest values within 1.5 times the IQR from the first and third quartiles, respectively.

**Extended Data Fig. 7:**
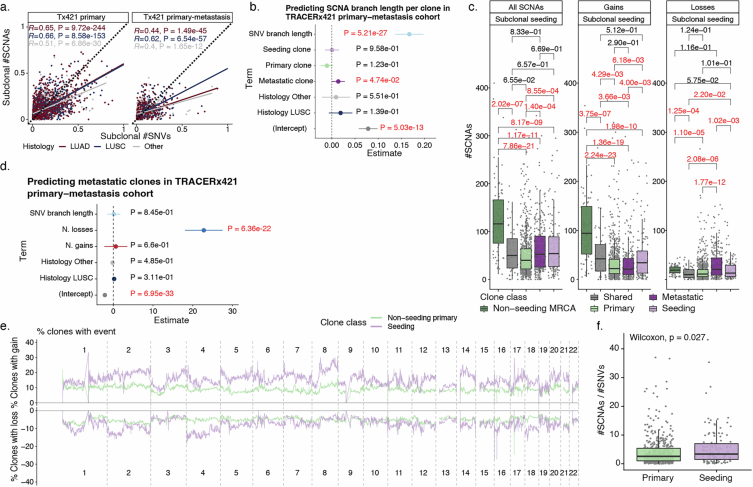
SCNA patterns in metastasis 1. **a**, Normalised number of SNVs versus normalised number of SCNAs (Pearson’s correlation) for every subclonal edge on the phylogenetic tree in the TRACERx421 primary (left) and primary-metastatic (right) cohorts. **b**, Estimated coefficients and p-value from a mixed-effects linear regression predicting dependent variable number of SCNAs on each tree edge (#SCNAs) from fixed explanatory variables: number of SNVs (#SNVs), metastatic clone class, histology, and random effect tumour ID **c**, The total number of SCNA events (quantified as number of gain breakpoints + loss breakpoints separating the parent and child node), number of gains and number of losses (Methods) is compared between clones of different classes in the metastatic transition, for tumours in which the MRCA was non-seeding (n = 76 tumours, with the following number of clones per class: Non-seeding MRCA n = 76, Shared n = 56, Primary n = 697, Metastatic n = 242, Seeding n = 121). Wilcoxon test significant P values are marked with red colour. **d**, Estimated coefficients and p-value from a mixed-effects logistic regression predicting binary dependent variable whether a clone is metastasis-unique from fixed explanatory variables: number of SNVs, number of gains, number of losses, histology, and random effect tumour ID. **e**, Fraction of seeding (purple) and non-seeding (green) clones with gain (top) or loss (bottom) at each genomic locus. **f**, Boxplot comparing the number of SCNAs/number of SNVs for seeding clones (n = 121) versus non seeding clones (n = 434), for tumours where the MRCA is not seeding (n = 76, two sided Wilcoxon).

To explore the SCNA patterns associated with metastasis, we classified clones in relation to the metastatic transition as follows: MRCA, shared, seeding, primary specific and metastasis specific. On each tree edge, we computed metrics of allele-specific copy number change, including per-segment change and total number of interval events (no. SCNAs; Fig. 5a and Methods) and observed inter- and intra-tumour variability in the slopes of tree paths, indicating that clones varied in rates of SNV and SCNA acquisition (Fig. 5b). Normalized subclonal numbers of SNVs and SCNAs were significantly and positively correlated (Extended Data Fig. 7a). However, the correlation was weaker when including metastatic clones, particularly in LUAD (Pearson’s *R* = 0.65 versus *R* = 0.44, for primary and primary–metastasis LUAD cohorts, respectively), which could reflect a different rate of acquisition of SCNAs in different stages of the metastatic transition. In the Tx421-PM cohort, the number of SCNAs per edge was significantly associated with both metastatic clones and number of SNVs in a linear mixed-effects model with histology as a fixed effect and tumour as a random effect (*P* = 0.0474 for metastatic clones, and *P* = 5.03 × 10^−13^ for number of SNVs; Extended Data Fig. 7b), and there was an increased number of SCNAs occurring in seeding and metastatic clones (Extended Data Fig. 7c).

To compare the SCNA acquisition rate between primary and metastatic clones, we fitted a mixed-effects logistic regression model to predict whether a subclone was metastasis specific on the basis of the number of SNVs, gains and losses, accounting for histology as a fixed effect and clones from the same tumour as a random effect. An elevated number of losses was significantly associated with the likelihood that a clone was metastatic in origin (*P* = 6.92 × 10^−22^; Extended Data Fig. 7d).

It is conceivable that seeding clones may harbour metastatic capacity as a result of an increased SCNA acquisition rate, increasing the likelihood of acquiring the genomic alterations required to initiate metastasis. We therefore aimed to compare the proportion of clones harbouring SCNA events between the primary tumour subclones that seed metastasis versus those that do not. We observed that for most genomic loci, a higher proportion of seeding clones than non-seeding clones harboured SCNAs: 96.4% and 81.5% of the genome showed more gains and losses, respectively, in seeding versus non-seeding clones (Extended Data Fig. 7e). The relationship held when comparing the overall number of SCNAs, gains and losses per edge in seeding versus non-seeding clones (Fig. 5c). Additionally, we observed that in primary tumour, seeding clones exhibited a higher SCNA/SNV ratio than non-seeding clones (*P* = 0.027, Wilcoxon test; Extended Data Fig. 7f).

**Extended Data Fig. 8:**
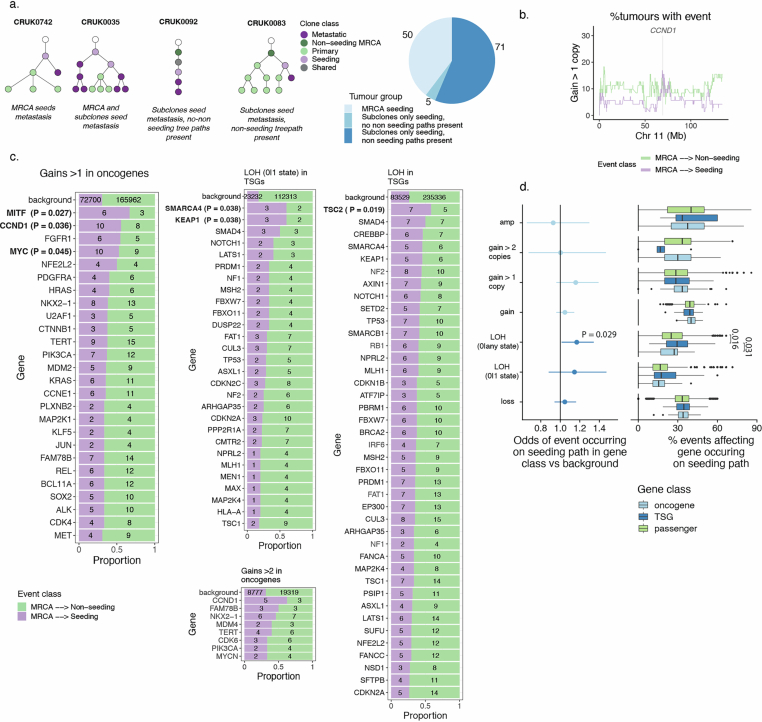
SCNA patterns in metastasis 2. **a**, Left: Example CRUK trees^6^ with nodes labelled by their clone class. The box around case CRUK0083 indicates an example of a tumour case that has (1) subclonal seeding only and (2) non-seeding tree paths present. Right: The number of tumours for which both seeding and non-seeding tree paths are present is shown in a pie chart, as a subset of the full TRACERx421 primary-metastasis cohort^6^. **b**, Chromosome 11 plot showing the number of tumours with gain of > 1 copy occurring on tree paths between the MRCA and the seeding clones (purple) and occurring on tree paths between the MRCA and non-seeding clones (green). **c**. Panels show the proportion of tumours affected by different SCNA events in seeding vs non-seeding paths (purple and green, respectively) compared to background of all genes of these types with $\ge 5$ such events (Methods). Oncogenes are considered for gains, TSGs are considered for losses. The background counts of such events in all genes are shown as the top entry in each panel. P values are annotated only for genes with P < 0.05. Fisher’s exact test. **d**. Left: Odds of occurrence of each of the event types on seeding paths vs non-seeding paths for oncogenes and TSGs compared to background of all genes. Right: Comparing %SCNA events affecting all genes (with $\ge 5$ such events) in seeding paths, split by SCNA event type and gene driver class. LOH (0|any state) describes the clone copy number state of 0 copies in one allele, and any copy number state in the other. LOH (0 | 1 state) describes the clone copy number state of 0 in one allele and 1 in the other (two sided Wilcoxon). For the purpose of discovery, results in **c** and **d** are presented without multiple testing correction.

We next investigated whether primary-tumour seeding clones in NSCLC acquired SCNAs at specific loci. We evaluated the subset of tumours containing both seeding and non-seeding clones (*n* = 71; Extended Data Fig. 8a). We compared the frequency of SCNAs on the ‘seeding path’ (the subclonal tree path of edges connecting the MRCA and the seeding clones) versus that on the ‘non-seeding path’ (the subclonal tree path connecting the MRCA and non-seeding clones). Gains of >1 copy affecting chr. 11q13.3 (*CCND1*) and 8q24.21 (*MYC*) and LOH with the remaining allele at 1 copy affecting chr. 19p13.2 (*SMARCA4* and *KEAP1*) were enriched in seeding paths versus non-seeding paths (Fisher’s exact test, *P* = 0.0362, *P* = 0.0454 and *P* = 0.0383, respectively; Extended Data Fig. 8b,c).

To test the combined impact on multiple drivers, we quantified the overall number of SCNAs affecting oncogenes and tumour suppressor genes (TSGs) compared to a background of all genes in seeding versus non-seeding paths (Methods). We observed a modest enrichment for LOH affecting TSGs (287 of 979; 29%) compared to a background (83,529 of 318,865; 26%) in seeding versus non-seeding paths (one-sided Fisher’s exact test, *P* = 0.0289; Extended Data Fig. 8d), possibly reflecting selection for losing TSGs before metastatic seeding.

## SCNA patterns mediate modes of dissemination

**Extended Data Fig. 9:**
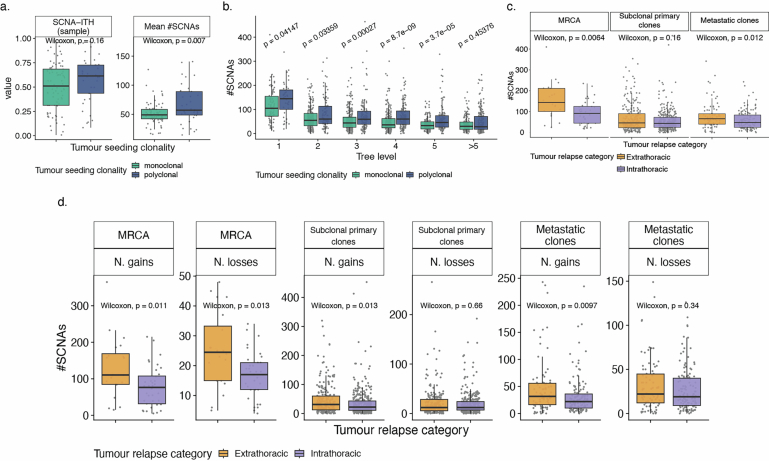
Modes of dissemination. **a**, Box plot comparing SCNA-ITH (sample level analysis, left) and mean number of SCNAs across all tree edges (clone-level analysis, right) for tumours in the TRACERx421 primary cohort between tumours exhibiting monoclonal (n = 86) and polyclonal (n = 40) dissemination patterns. Each point represents one tumour. **b**, Box plots comparing number of SCNA events per edge split between monoclonal and polyclonal seeding tumours, grouped by tree level Total number of clones in monoclonal and polyclonal seeding tumours are n = 1170, and n = 677, respectively. **c**, Box plot comparison of SCNA count per clone in patients with exclusively extrathoracic (n = 20) and exclusively intrathoracic (n = 36) seeding. **d**, Boxplot comparison of number of SCNAs, number of gains and number of losses per clone in patients with exclusively extrathoracic (n = 20) and exclusively intrathoracic (n = 36) seeding. All tests in this figure are two-sided Wilcoxon.

We previously observed that high SCNA intra-tumour heterogeneity (SCNA-ITH) was associated with increased likelihood of an extrathoracic relapse, and that extrathoracic relapse was associated with polyclonal dissemination (in which multiple primary clones are the founders of metastatic sites)^5,6^. Therefore, we explored whether polyclonal seeding is associated with increased SCNA-ITH in the primary tumour. When comparing SCNA-ITH scores computed on a sample level^5^ within primary tumours that metastasize through monoclonal (*n* = 80) versus polyclonal (*n* = 37) seeding, we observed no significant difference. However, using the results generated by ALPACA, we observed a significantly higher average number of SCNAs per primary clone in tumours with polyclonal versus monoclonal seeding (*P* = 0.007, Wilcoxon test; Extended Data Fig. 9a). An increased number of SCNA events was also visible at an individual clone level, when comparing clones from monoclonal versus polyclonal seeding tumours in the Tx421-PM cohort^6^, classifying clones by their tree level (defined as the number of ancestors separating each clone from the MRCA; Extended Data Fig. 9b).

We further compared the number of SCNA events by location of metastasis, and found that clones from patients who had only extrathoracic metastasis (*n* = 30 tumours), as opposed to exclusively intrathoracic metastases (*n* = 36), had significantly more SCNAs both in the MRCA and metastatic clones (Wilcoxon test, *P* = 0.0064 and *P* = 0.012, respectively; Extended Data Fig. 9c). Additionally, we observed an enrichment for gains in the MRCA, subclonal primary tumour clones and metastatic clones (Wilcoxon test, *P* = 0.011, *P* = 0.013 and *P* = 0.0097, respectively; Extended Data Fig. 9d). Taken together, these findings may indicate that CIN acquired early in tumour evolution confers (potentially multiple) tumour clones the potential to seed extrathoracic metastases, and this phenotype of increased CIN, especially the acquisition of copy-number gains in patients with extrathoracic metastases, is maintained throughout tumour evolution.

## Clone copy number diversity is prognostic

**Fig. 6:**
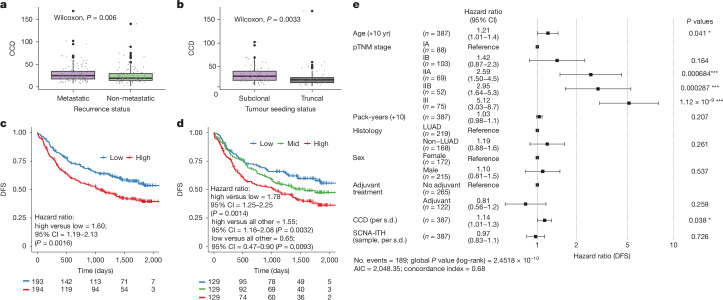
CCD predicts poor patient survival. **a**, CCD comparison (two-sided Wilcoxon) between tumours of patients with (*n* = 117 tumours) and without (*n* = 126 tumours, 3-yr follow-up period^6^) metastatic disease. **b**, CCD comparison between tumours with only subclonal (*n* = 73 tumours) or truncal (*n* = 44 tumours) seeding. **c**,**d**, Kaplan–Meier curves showing difference in DFS between patients with tumours with greater or less than the median value of CCD (median CCD = 21.95; **c**) and with high, mid or low tertile values of CCD (**d**). The number of patients at risk in each group is indicated below each time point. CI, confidence interval. **e**, A multivariable Cox proportional hazards model including covariates age, stage, pack-years, histology, sex, adjuvant treatment status, CCD (increase per standard deviation) and fraction of the aberrant genome with subclonal SCNAs (SCNA-ITH (sample), increase per standard deviation). The measure of centre represents the hazard ratio estimate and its 95% confidence intervals are indicated in parentheses and represented by the error bars. All survival analyses were performed on *n* = 387 patients. pTNM, pathological tumour–node–metastasis; AIC, Akaike information criterion. *P < 0.05; **P < 0.01; ***P < 0.001. In data represented using box plots, the box represents the interquartile range (IQR) with the median line. Whiskers denote the lowest and highest values within 1.5 times the IQR from the first and third quartiles, respectively.

**Extended Data Fig. 10:**
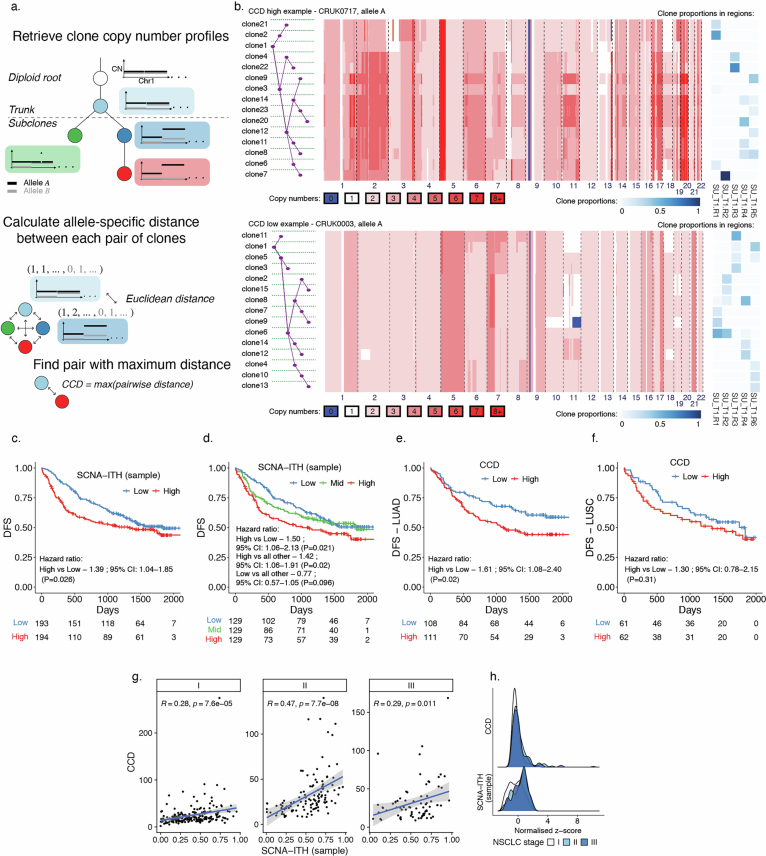
Clone copy number diversity predicts poor patient survival. **a**, Diagram describing the calculation of the clone copy number diversity metric (CCD). **b**, Inferred clone copy number from allele A from example cases CRUK0717, which was classified as having high clone copy number diversity, and CRUK0003, which was classified as having low clone copy number diversity. **c**, Kaplan-Meier curve showing the difference in DFS between patients harbouring tumours with greater (red) or less (blue) than the median value of SCNA-ITH (sample) (median = 0.497). The number of patients at risk in each group is indicated below each timepoint. **d**, Kaplan-Meier curve showing the difference in DFS between tumours with high, mid, or low values of SCNA-ITH (sample). **e**, Kaplan-Meier curve showing the difference in DFS between patients diagnosed with lung invasive adenocarcinoma harbouring tumours with greater or less than the median value of CCD (median = 20.2). **f**, Kaplan-Meier curve showing the difference in DFS between patients diagnosed with lung squamous cell carcinoma harbouring tumours with greater or less than the median value of CCD (median = 23.5). **g**, Correlation between SCNA-ITH (sample) and CCD for tumours within each TNM stage. **h**, Z-normalised density of SCNA-ITH (sample) and CCD values across the patient cohort, by TNM tumour stage.

Given the association between increased SCNA activity and metastasis, we reasoned that SCNA diversity in the primary tumour may be associated with survival. To obtain an estimate of SCNA diversity in primary tumours, we calculated the maximum pairwise Euclidean distance between the copy-number profiles of the primary tumour clones (clone copy number diversity (CCD); Extended Data Fig. 10a,b). Within the Tx421-P cohort, patients with metastatic disease (*n* = 117) had significantly higher CCD (Wilcoxon test, *P* = 0.006; Fig. 6a) compared to patients who did not relapse within the follow-up period (*n* = 126^10^). We next compared the CCD of primary tumours of patients with metastatic disease with only subclonal seeding from the primary tumour (*n* = 73) versus those with metastasis seeded by the MRCA (*n* = 44). We found that CCD was significantly higher for subclonal seeding tumours (Wilcoxon test, *P* = 0.0033; Fig. 6b). Taken together, these results indicate that the diversity of different clone-specific SCNAs might facilitate development of metastasis.

We previously demonstrated that SCNA-ITH (measured as the fraction of the aberrant genome with subclonal SCNAs, here termed SCNA-ITH (sample)) was associated with a poor disease-free survival (DFS)^5^, but the effect was not consistent across time (Extended Data Fig. 10c), and the metric did not predict a difference in DFS when splitting the cohort into tertiles and comparing patients with low SCNA-ITH (sample) versus all others (Extended Data Fig. 10d). We performed a similar analysis using CCD and observed that within the same patient cohort, patients with lower CCD had consistently better DFS when splitting the cohort by the median (log-rank test, *P* = 0.0016; Fig. 6c) and by tertiles (log-rank test, high versus low, *P* = 0.0014; high versus all other, *P* = 0.0032; low versus all other, *P* = 0.0093; Fig. 6d), indicating that the higher-resolution clone-level CCD metric is able to better stratify patients. The association of higher CCD with poorer DFS was observed most strongly in LUAD (Extended Data Fig. 10e,f). Although CCD and SCNA-ITH (sample) were significantly positively correlated, particularly in tumour–node–metastasis stage II tumours (Pearson *R* = 0.47, *P* = 7.7 × 10^−8^; Extended Data Fig. 10g), the distribution of CCD values across the cohort was more symmetrical, despite having a long tail for high values (Extended Data Fig. 10h). Finally, combining CCD with the clinical variables age, stage, pack-years, histology, adjuvant therapy status and SCNA-ITH (sample) in a multivariable model, we found that CCD, but not SCNA-ITH (sample), remained a significant predictor of DFS (hazard ratio = 1.14, 95% confidence interval = 1.01–1.3, log-rank test, *P* = 0.038; Fig. 6e).

## Discussion

Despite the clinical significance of SCNAs in cancer, a detailed understanding of clone-level SCNA evolution is lacking. Here we introduce ALPACA, a computational method to infer clone- and allele-specific copy numbers from bulk multi-sample DNA-sequencing data. The ALPACA method is founded on the idea that tumour evolution can be reconstructed in a two-step process: first, reconstruct the evolution of SNVs alone; then use the reconstructed tumour phylogeny and clone proportions as a scaffold to infer the evolution of SCNAs. This reduces the copy-number mixture deconvolution problem into a simpler linear optimization task, in which only integer copy numbers are inferred, and clone prevalence is assumed to be known. Clone-level analysis using ALPACA has enabled new clinical insights. We observed that metastasis-seeding clones had higher levels of CIN than primary clones that did not seed metastasis, a correlation between the number of copy-number events occurring in a clone and its seeding modality (monoclonal versus polyclonal), as well as the anatomical location of relapse lesions (extrathoracic versus intrathoracic), and an association between CCD and survival in lung cancer. When analysing recurrent patterns of copy number changes, we found gains in *CCND1* and *MYC* to be enriched in subclonal tree paths preceding metastatic seeding. Gains at these gene loci have previously been identified as positively selected in metastatic samples in our recent NSCLC metastasis study^6^ and in LUAD–brain metastasis^39^. However, clone-level copy numbers obtained using ALPACA suggest that these gains occur in primary tumour clones before metastatic seeding, and do so more frequently than in non-metastasizing subclones. This is consistent with a related TRACERx421 study that observed higher transcriptional signatures of proliferation in tumour samples harbouring the seeding clone^40^. We additionally observed enrichment for LOH in TSGs in evolutionary paths preceding metastatic seeding, indicative of copy-number selection occurring in primary tumour subclones with metastatic potential. Using a sample-level SCNA analysis would have identified these changes to have occurred in the metastatic site itself, only after seeding^6^, thereby masking subclonal copy-number selection present in the primary tumour.

The framework outlined in this paper may provide a foundation for the development of more evolutionary-aware copy-number signatures. For example, copy-number signatures have recently begun to shed light on recurrent patterns of copy number alterations across several cancer types^41–43^. However, at present, these consider the aggregate of all copy number alterations together, without distinguishing their temporal acquisition or the fact that many copy number alterations build on each other. The ALPACA method also enables the analysis of specific classes of clones; for example, interrogation of copy-number profiles and changes associated with the MRCA or clones that show evidence of recent subclonal expansion.

Nevertheless, the proposed approach has limitations. ALPACA requires multi-sample datasets as an input, and the results depend on reliable SNV and SCNA calls, the quality of the reconstructed SNV tree and the clone proportions derived from the upstream processing. In particular, errors in the copy-number correction during these processes might lead to incorrect cancer cell fractions and phylogeny. Indeed, incorporating uncertainty in the input clone proportions to ALPACA could be leveraged in a future improvement to the ALPACA method, whereby clone proportion estimates could be refined after clone copy numbers are derived, in a two-step process, as previously proposed^2,3,31^. In this study, we used CONIPHER^21^, which provided robust phylogenetic tree topologies for input to ALPACA (Extended Data Fig. 1d and Supplementary Information).

In conclusion, ALPACA provides a framework to easily explore the evolution of SNVs and SCNAs jointly at the resolution of clones, from bulk DNA-sequencing data, and sheds light on the importance of SCNAs as a key process underpinning NSCLC tumour evolution to a more aggressive, metastatic phenotype, leading to a poorer patient survival.

## Methods

### The ALPACA algorithm

We introduce ALPACA, an algorithm to infer allele-specific copy-number profiles of every tumour clone, given three inputs that can be inferred by existing methods: estimates of allele-specific fractional copy number from multiple bulk tumour samples; a fixed number of clones and the proportion of each clone in each tumour sample; and a fixed topology of the tumour phylogenetic tree representing the phylogenetic relationships between all clones. We provide an overview of the model underlying ALPACA, the formulation of the problem that ALPACA aims to solve, and the ALPACA algorithms used to solve it. Additional details can be found in the Supplementary Information.

#### Copy-number evolutionary model

We assume that a tumour is composed of $n$ distinct tumour clones with their genome partitioned into $m$ copy-number segments owing to the effect of the SCNAs that have been accumulated through tumour evolution. As such, each tumour clone $i\in \{1,\,...,{n}\}$ in each genomic segment $s\in \{1,\,...,{m}\}$ has a number ${c}_{s,i}^{{\rm{A}}}$ of copies for allele A, and ${c}_{s,i}^{{\rm{B}}}$ for allele B. For a normal diploid clone $i=0$, we know that ${c}_{s,0}^{{\rm{A}}}\,=\,{c}_{s,0}^{{\rm{B}}}\,=\,1$. Instead, for any tumour clone $i\in \{1,\,...,{n}\}$, the copy numbers ${c}_{s,i}^{{\rm{A}}}$ and ${c}_{s,i}^{{\rm{B}}}$ are unknown, and ALPACA aims to infer them.

We model the evolution of the $n$ tumour clones with a phylogenetic tree $T=(V,{E})$, in which $V$ represents the set of nodes and $E$ represents the related set of edges, which also defines the topology of $T$. Specifically, each clone $i$ is represented by a vertex ${v}_{i}$ of the tree; that is, ${v}_{i}\in V$. If tumour clone $i$ is a parent of clone $j$, then $({v}_{i},{v}_{j})$ is an edge of $T$; that is, $({v}_{i},{v}_{j})\in E$. We assume, without loss of generality, that the MRCA of the tumour clones is clone $i=1$. We also assume that the normal clone $i=0$ is the root vertex of the tree ${v}_{0}\in V$, such that $({v}_{0},{v}_{1})\in E$.

As we model SCNA evolution of the $n$ tumour clones, each node of the tree $T$ is labelled by related copy numbers for all segments. Specifically, for each tumour clone $i\in \{1,\,...,{n}\}$, the corresponding vertex ${v}_{i}\in V$ is labelled in each segment $s\in \{1,\,...,{m}\}$ by the copy number ${c}_{s,i}^{{\rm{A}}}\in {\mathbb{N}}$ for allele A and ${c}_{s,i}^{{\rm{B}}}\in {\mathbb{N}}$ for allele B. The normal root clone at vertex ${v}_{0}$ is labelled by ${c}_{s,0}^{{\rm{A}}}=\,1$ for allele A and ${c}_{s,0}^{{\rm{B}}}=1$ for allele B. Each edge of the tree $({v}_{i},{v}_{j})\in E$ is thus labelled with events that are required to transform the copy numbers of clone $i$ to those of clone $j$. In particular, similarly to previous copy-number evolutionary models^14,15,17^, we model each copy-number event for each segment independently as an event that either increases or decreases a copy number by one unit. As such, the label of each edge corresponds to ${| {c}_{s,i}^{{\rm{A}}}-{c}_{s,j}^{{\rm{A}}}| }_{1}\,+$ ${| {c}_{s,i}^{{\rm{B}}}-{c}_{s,j}^{{\rm{B}}}| }_{1}$ events for each genomic segment $s$, as ${| {c}_{s,i}^{{\rm{A}}}-{c}_{s,j}^{{\rm{A}}}| }_{1}$ events are required for allele A of each segment $s$ and ${| {c}_{s,i}^{{\rm{B}}}-{c}_{s,j}^{{\rm{B}}}| }_{1}$ for allele B.

Given a fixed topology for a tree $T$, an arbitrarily high number of copy-number labellings for $T$ can be defined in general to explain SCNA evolution of the $n$ tumour clones. Therefore, we introduce an evolutionary model based on three biologically realistic assumptions that define the best copy-number-labelled tree $T$ to represent SCNA evolution. First, similarly to previous models of SCNA evolution^4,31^, we assume that LOH events are irreversible; that is, if all of the copies of one allele in any segment are lost, these cannot be regained by any descendant. Specifically, for any segment $s\in \{1,\,...,{m}\}$ in any edge $({v}_{i},{v}_{j})\in E$ it holds that ${c}_{s,i}^{{\rm{A}}}=0\,\Rightarrow {c}_{s,j}^{{\rm{A}}}=\,0$ and ${c}_{s,i}^{{\rm{B}}}=0\,\Rightarrow {c}_{s,j}^{{\rm{B}}}=\,0$. Second, we limit the changes between gains and losses of the same genomic segment that occur along each evolutionary path to prevent overfitting (by default, the upper bound is 2; that is, only one type of change (negative change (loss) or positive change (gain)) can happen multiple times on a single path), whereby a path refers to the sequence of edges $\{({v}_{0},{v}_{1}),\,...,({v}_{i-1},{v}_{i})\}$ connecting the diploid root clone ${v}_{0}$ to any node ${v}_{i}$. Last, under the assumption of parsimony, we define the tree with the least number of copy-number events that explain the input data to be the more likely explanation of SCNA evolution, similarly to previous approaches^4,31^.

#### Model of the sequencing experiment

Each bulk tumour sample comprises an unknown mixture of the $n$ tumour clones. When sequencing $k$ bulk tumour samples, we thus represent the proportion of each clone $i\in \{0,\,...,{n}\}$ in each tumour sample $r\in \{1,\,...,{k}\}$ as ${u}_{i,r}\in [0,\,1]$. Specifically, ${u}_{i,r} > 0$ indicates that clone $i$ is present in sample $r$, and absent otherwise. We assume that, within each tumour sample $r$, the tumour purity (that is, the proportion of all tumour clones) is ${\sum }_{i=1}^{n}{u}_{i,r}=1-{u}_{0,r}$, in which ${u}_{0,r}$ represents the proportion of normal cells in sample $r$.

We cannot directly observe the copy numbers ${c}_{s,i}^{{\rm{A}}}$ and ${c}_{s,i}^{{\rm{B}}}$ of each tumour clone $i$ in each tumour sample from bulk DNA-sequencing data. However, existing methods^28–30,45,46^ can provide estimates of the allele-specific, fractional copy numbers ${\widehat{f}}_{s,r}^{{\rm{A}}}$, ${\widehat{f}}_{s,r}^{{\rm{B}}}$ of each segment $s$ in each tumour sample $r$, such that ${\widehat{f}}_{s,r}^{{\rm{A}}},{\widehat{f}}_{s,r}^{{\rm{B}}}\in {{\mathbb{R}}}^{\ge 0}$ are real-valued observed estimates of the average copy number of all cancer cells in the sample ${f}_{s,r}^{{\rm{A}}}=\,{\sum }_{i=1}^{n}{c}_{s,i}^{{\rm{A}}}{u}_{i,r}$ and ${f}_{s,r}^{{\rm{B}}}=\,{\sum }_{i=1}^{n}{c}_{s,i}^{{\rm{B}}}{u}_{i,r}$, respectively. Using existing methods, we can also observe estimates of the upper bound, ${f}_{s,r}^{{\rm{A}},+},\,{f}_{s,r}^{{\rm{B}},+}\in {{\mathbb{R}}}^{\ge 0}$, and lower bound, ${f}_{s,r}^{{\rm{A}},-},\,{f}_{s,r}^{{\rm{B}},-}\in {{\mathbb{R}}}^{\ge 0}$, of ${f}_{s,r}^{{\rm{A}}}$ and ${f}_{s,r}^{{\rm{B}}}$, for each sample $r$ and genomic segment $s$. The fractional copy number estimates, ${\widehat{f}}_{s,r}^{{\rm{A}}}$, ${\widehat{f}}_{s,r}^{{\rm{B}}}$, and their associated upper and lower bound estimates $[{f}_{s,r}^{{\rm{A}},-},{f}_{s,r}^{{\rm{A}},+}]$ and $[{f}_{s,r}^{{\rm{B}},-},{f}_{s,r}^{{\rm{B}},+}]$ represent measurements obtained from standard DNA-sequencing experiments.

#### Problem formulation

Hence, given allele-specific estimates of fractional copy numbers from multiple bulk DNA-sequenced tumour samples, ALPACA aims to infer the integer clone copy-number values ${c}_{s,i}^{{\rm{A}}}$ and ${c}_{s,i}^{{\rm{B}}}$ that minimize the objective functions above, similarly to the clone copy-number mixture deconvolution problem explored in previous work^2,3,15,16,19,31^. In contrast to existing methods, ALPACA does not however aim to jointly infer the number of clones present $n$, and their clone proportions ${u}_{i,r}$. Instead, as described above, the hypothesis underlying ALPACA is that copy-number inference from fractional copy numbers can be improved by integrating other data that can be inferred by existing methods, in addition to the estimated fractional copy numbers: the topology of the tree $T$ defined by the related edges $E$, and the proportion ${u}_{i,r}$ of each clone $i$ in each sample $r$. Using fixed clone proportions is expected to improve the performance of copy-number inference because it simplifies the complex copy-number deconvolution problem into an easier linear optimization problem.

In fact, the tree, $T$, that is taken as input to ALPACA can be derived from standard approaches for phylogenetic reconstruction from SNVs^21–24,26,27^. Similarly, clone proportions of the tumour clones ${u}_{1,r},\,...,\,{u}_{n,r}$ in sample $r$ can be derived from standard approaches for tumour clonal reconstruction using SNVs^21–26,47–49^, and we assume that the tumour purity of each sample $r$ (that is, $(1-{u}_{0,r})$) can be estimated from standard approaches for tumour deconvolution from bulk DNA sequencing (for example, ASCAT^28^ and Sequenza^29^).

Overall, ALPACA thus aims to infer the unknown copy numbers of each clone in a tree $T$ with a given topology (that is, given the set of edges $E$) and given the fractional copy numbers in each sequenced tumour sample. Formally, ALPACA is framed as a linear optimization algorithm, aiming to find the copy numbers ${c}_{s,i}^{{\rm{A}}}$ and ${c}_{s,i}^{{\rm{B}}}$ for all clones $i\in \{1,\,...,{n}\}$ in all segments $s\in \{1,\,...,{m}\}$ that satisfy the following model objectives:

1. Given the observed, allele-specific fractional copy number estimates in each sample ${\widehat{f}}_{s,r}^{{\rm{A}}},{\widehat{f}}_{s,r}^{{\rm{B}}}$, their upper and lower bound estimates $[\,{f}_{s,r}^{{\rm{A}},-},{f}_{s,r}^{{\rm{A}},+}\,]$ and $[\,{f}_{s,r}^{{\rm{B}},-},{f}_{s,r}^{{\rm{B}},+}\,]$, the clone proportions in each sample ${u}_{i,r}$, and constraints imposed by the phylogenetic tree $T$, find ${c}_{s,i}^{{\rm{A}}},{c}_{s,i}^{{\rm{B}}}\in {\mathbb{N}}$ that minimize the number of samples for which ${\sum }_{i=1}^{n}{c}_{s,i}^{{\rm{A}}}{u}_{i,r}\,\notin$ $[\,{f}_{s,r}^{{\rm{A}},-}\,,{f}_{s,r}^{{\rm{A}},+}\,]$ or ${\sum }_{i=1}^{n}{c}_{s,i}^{{\rm{B}}}{u}_{i,r}\notin [\,{f}_{s,r}^{{\rm{B}},-},{f}_{s,r}^{{\rm{B}},+}\,]$.
2. Among these solutions, find ${c}_{s,i}^{{\rm{A}}}$ and ${c}_{s,i}^{{\rm{B}}}$ that minimize the absolute difference (Manhattan distance) between the predicted and expected fractional copy number, ${| {\widehat{f}}_{s,r}^{{\rm{A}}}-{\sum }_{i=1}^{n}{c}_{s,i}^{{\rm{A}}}{u}_{i,r}| }_{1}$ and ${| {\widehat{f}}_{s,r}^{{\rm{A}}}-{\sum }_{i=1}^{n}{c}_{s,i}^{{\rm{B}}}{u}_{i,r}| }_{1}$.

ALPACA solves this optimization problem using an algorithm based on a mixed-integer linear program (MILP implemented with gurobipy, v11.0.1) and a model selection approach defined in the following section and in the Supplementary Information.

#### Inferring clone-specific copy numbers

The ALPACA algorithm solves the problem stated above using a MILP and a model selection approach^2,3,15,31^. Specifically, given a fixed number of copy-number events ${\lambda }_{s}\in {\mathbb{N}}$, ALPACA identifies the copy numbers that minimize the above model objectives 1 and 2 under the previously described constraints, using an MILP formulation (details are reported in Supplementary Information). That is, for each segment $s$, we aim to find ${c}_{s,i}^{{\rm{A}}}$ and ${c}_{s,i}^{{\rm{B}}}$ that minimize the objective functions subject to constraints described above, such that the total number of copy-number events across all edges $({v}_{i},{v}_{j})$ of the tree $T$ (including both gains and losses), and both alleles is less than ${\lambda }_{s}$. ALPACA performs this optimization for each segment independently, as the evolution of each segment is modelled independently for the fixed tree topology. This allows ALPACA to scale to tumours with a high burden of subclonal SCNAs and thus a high number of segments at once.

The number of copy-number events ${\lambda }_{s}$ that occur throughout evolution is unknown. Therefore, similarly to previous studies^4,31^, we apply a model selection approach to find ${\lambda }_{s}$ while finding the best compromise between solutions with higher complexity (higher values of ${\lambda }_{s}$) and those with better fit of the data (lower values of the objective function). Specifically, we consider an increasing number of copy-number events ${\lambda }_{s}$, and we select the best number of events, $\hat{{\lambda }_{s}}$, using the elbow approach implemented by the Kneedle algorithm (kneed v0.8.5)^50^. Further details on the model selection algorithm used by ALPACA are available in the Supplementary Information.

The ALPACA algorithm outputs inferred clone copy numbers ${c}_{s,i}^{{\rm{A}}}$ and ${c}_{s,i}^{{\rm{B}}}$ for each segment $s\in \{1,\,...,{m}\}$ within every ancestral and observed tumour clone in the $k$ sequenced samples.

#### Run time

In the experimental TRACERx421 primary cohort, the total CPU time for a single tumour ranged from 48 s to 36 h with a median run time of 19 min. The differences in run time across tumours can be partially explained by the number of segments (ALPACA iterates over each segment independently) and by the number of SNV clones detected in a tumour; mean purity and ploidy of tumours show only a very weak correlation with the CPU run time (Extended Data Fig. 2). Iterating over genomic segments independently allows for a very efficient parallelization, in which segments predicted to complete quickly can be grouped into large batches, and segments predicted to complete slowly can be grouped into small ones.

### Benchmarking ALPACA on simulated data

#### MASCoTE simulations

##### Creating input

We used the previously published simulation framework MASCoTE^2^ to simulate multi-sample bulk sequencing, using the following steps.

First, to create the input required by ALPACA, we mapped the 50-kilobase binned simulated data to the ground-truth segments, represented by $S$. Second, we calculated the average read depth $R$ of each segment $s\in \{1,\,...,{|S|}\}$ and each sample $r\in \{1,\,...,{k}\}$, ${R}_{s,{r}}$, by averaging $R$ across all bins $b\in B$ overlapping with a segment $s$:

${R}_{s,{r}}=\frac{1}{|{B}_{s}|}\sum _{b\in {B}_{s}}{R}_{b}$, in which $|{B}_{s}|$ is the number of bins overlapping with segment $s$, and ${R}_{b}$ is the read depth of bin $b$. Using the same method, we then calculated the BAF $B$ for each segment $s$ and each sample $r$, using the following equation:

$${B}_{s,r}=\frac{1}{|{B}_{s}|}\sum _{b\in {B}_{s}}{B}_{b}$$

For each segment $s$ and each sample $r$, we then determined the single nucleotide polymorphism (SNP) confidence intervals by fitting a *t*-distribution to the read depth values within each segment and sample. To simulate the experimental noise, we first calculated the true fractional copy numbers ${F}_{T}$ for each sample $r$ and segment $s$ by taking the dot product of two vectors: the vector representing the clone proportions in the sample ${{\bf{U}}}_{{\bf{r}}}$ and the vector of integer copy numbers of the clones present in the sample, ${\bf{C}}$:

$${F}_{T}={{\bf{U}}}_{{\bf{r}}}\cdot {\bf{C}}$$

Using the true fractional copy numbers ${F}_{T,r}$ and read depth ${R}_{r}$ for each sample $r$, we derived a scaling factor for each segment and each sample, ${\varGamma }_{S,R}$, by dividing ${F}_{T,r}$ by the ${R}_{r}$ values for each segment $s$ and each sample $r$. By taking a mean of ${\varGamma }_{s,r}$ across all segments for each sample, we obtained the mean scaling factor per sample $\bar{{\varGamma }_{r}}$:

$${\varGamma }_{s,r}=\frac{{F}_{T,s,r}}{{R}_{s,r}}$$

$$\bar{{\varGamma }_{r}}=\frac{1}{|S|}\sum _{s\in S}{\varGamma }_{s,r}$$

By multiplying the mean scaling factor per sample, ${\varGamma }_{r}$, by $R$ of each segment in each sample, we obtained the final total fractional copy numbers, $F$, for each segment in each sample:

$${F}_{s,r}=\bar{{\varGamma }_{r}}\times {R}_{s,r}$$

Last, using previously calculated $B$, we obtained fractional copy numbers for alleles A and B (${F}^{{\rm{A}}}$ and ${F}^{{\rm{B}}}$) for each segment and each sample:

$${F}_{s,r}^{{\rm{A}}}={F}_{s,r}\times ({1-B}_{s,r})$$

$${F}_{s,r}^{{\rm{B}}}={F}_{s,r}\times {B}_{s,r}$$

##### Accuracy calculation

To compare ALPACA, HATCHet, HATCHet2 and cloneHD results using MASCoTE simulations, we used the accuracy metric adapted from a similar metric used in the HATCHet2 publication^3^. Let ${S}_{s}$ signify the set of allele-specific, integer copy-number states inferred by a model for a specific genomic segment $s$. Let ${T}_{s}$ signify the set of true integer copy-number states for segment $s$. Let ${l}_{s}$ signify the length of segment $s$ (expressed in base pairs), and let $L=\sum _{s}{l}_{s}$ represent the total length of genomic segments under consideration. The accuracy of a specific model can then be represented as

$${\rm{Accuracy}}=\frac{1}{L}{\Sigma }_{s}{l}_{s}\frac{| {S}_{s}\bigcap {T}_{s}| }{| {S}_{s}\bigcup {T}_{s}| }$$

#### Simple model

##### Tx simulations in simple model

To recapitulate the clonal and SCNA landscape of NSCLC, we used the previously described simulated cohort^5,21^, which provided us with phylogenetic trees, copy-number events across the genome and clone proportions in each tumour sample. To obtain the ALPACA input using these data, we calculated the true fractional copy number for each simulated genomic segment and matched it with a real segment from the TRACERx421 primary cohort^5^ (randomly selected from a pool of segments with similar length and fractional copy number: tolerance ±10%). To obtain confidence intervals of these fractional copy-number values per segment, we assigned SNPs of the true segment to the simulated segment, and corrected the copy numbers of the assigned SNPs by the difference between the fractional copy-number states of the simulated segment and the true matched segment.

##### Implementation

The simple model was implemented similarly to the method described previously^5^. In brief, for each segment, clone and allele, the clone-specific copy number was computed as a rounded fractional copy number (to the nearest integer) of a sample in which a given clone was most prevalent, on the basis of the difference in cancer cell fraction values between samples in which the event is and is not present. For clones that have no representation in any sample, copy number was inherited from the closest ancestor. If no ancestors were available for a clone, this clone’s copy number was assigned to take the copy-number states of its children.

#### Matching comparison between methods

##### Creating input

To evaluate the ability of ALPACA to accurately infer the evolution of SCNAs, we extended the previously created realistic simulated instances that modelled SNV evolution (Tx simulations)^5,21^. This simulated dataset contained 2,704 clones and 87,393 genomic segments, amounting to 3,179,926 clone- and allele-specific segments. These simulated instances modelled not only truncal and subclonal SNVs, but also the effect of multiple other genomic alterations such as truncal and subclonal SCNAs and whole-genome duplication on SNV evolution, enabling their use to evaluate the reconstruction of SCNA evolution as well as SNV evolution. Additionally, the simulations were developed to reflect real non-small cell lung cancer phylogenies by using distributions of statistics measured from sequencing data for the TRACERx421 cohort^5^, such as the number of samples per simulation, the frequency of SCNAs and the proportion of events occurring truncally or subclonally. We used these simulations to evaluate the performance of phylogeny reconstruction using CONIPHER^21^ and ALPACA, and compared the results to the existing state-of-the-art copy-number reconstruction tools HATCHet2 (ref. ^3^) and MEDICC2 (ref. ^4^).

To reconstruct copy-number evolution, each method required sequencing information across the entire genome. However, as the Tx simulation framework was developed to simulate SNV evolution, the instances contained simulated sequencing data for only select regions of the genome (those affected by simulated SNVs). We therefore located the positions across the simulated genome with copy-number information and defined the size of the copy number alterations to cover between 50 and 100 mb, resulting in a simulated genome of 1-mb bins, for which each bin is denoted by $i$. Any bins containing more than one simulated SNV were split into multiple smaller bins. For each sample $s$, the ploidy ${\rho }_{s}$ and purity ${\mu }_{s}$ values from the Tx simulated sequencing data were used.

The sequencing depth of each bin, or the observed total number of reads ${t}_{i,s}$, was modelled using a Poisson distribution with the mean equal to the expected total number of reads present in each bin $i$ for all cells in each sample $s$. The expected total number of reads can be calculated from the true fractional copy number ${f}_{i,s}$, in which ${f}_{i,s}$ corresponds to the average total copy number of each bin $i$ for all cells present in sample $s$. The values of ${f}_{i,s}$, as well as the expected allele-specific fractional copy number for allele A ${x}_{i,s}$ and allele B ${y}_{i,s}$, for bins containing a SCNA were populated using the respective true fractional copy number values calculated in the Tx simulations, whereas the values for any bins without a SCNA were populated with either a diploid or tetraploid copy-number value depending on the most common value in the simulated sequencing data for each sample. The expected total number of reads is equal to $\frac{{f}_{i,s}}{{\rho }_{s}}{\gamma }_{s}$, in which ${\gamma }_{s}$ represents the expected average coverage per sample. ${\gamma }_{s}$ was set to a value of 1,000 for all samples $s$ in all simulated instances. Therefore, we simulated ${t}_{i,s}$ as drawn from a Poisson distribution with the mean equal to the expected number of total reads ${t}_{i,s}\, \sim {\rm{Poisson}}\left(\frac{{f}_{i,s}}{{\rho }_{s}}{\gamma }_{s}\right)$.

We next used a binomial model to simulate the observed B-allele frequency (BAF) of each bin. We defined the true BAF ${\beta }_{i,s}$ for each bin $i$ and sample $s$ on the basis of the fractional copy number of both alleles ${f}_{i,s}$ and allele B ${y}_{i,s}$ as ${\beta }_{i,s}\,=\,\frac{{y}_{i,s}}{{f}_{i,s}}$. We then modelled the observed BAF ${\hat{\beta }}_{i,s}$ of each bin using the binomial distribution with the probability of success equal to ${\beta }_{i,s}$ and the number of trials equal to a simulated coverage value ${\gamma }_{s}$ of 1,000, such that ${\widehat{\beta }}_{i,s}\, \sim \,{\rm{binomial}}({\gamma }_{s},{\beta }_{i,s})$.

The observed fractional copy-number values for each bin $i$ and sample $s$ were then calculated for allele A ${\hat{x}}_{i,s}$ and allele B ${\hat{y}}_{i,s}$ on the basis of the observed total number of reads present in each bin ${t}_{i,s}$, the ploidy of the sample ${\rho }_{s}$, and the observed BAF ${\hat{\beta }}_{i,s}$ using the following equations for allele A and allele B, respectively: ${\hat{x}}_{i,s}\,={t}_{i,s}\times {\rho }_{s}\times (1-{\hat{\beta }}_{i,s})$, ${\hat{y}}_{i,s}\,={t}_{i,s}\times {\rho }_{s}\times {\hat{\beta }}_{i,s}$.

##### Parameters used for HATCHet2 and MEDICC2

Using our set of ground-truth simulations (Tx simulations), we compared the performance of CONIPHER + ALPACA to HATCHet2 + MEDICC2 to accurately infer copy-number evolution. HATCHet2 (v2.0.1) was run with default parameters, except for an increase in the possible number of clones identified. A maximum value of 12 clones was used for simulations from LTXSIM001 to LTXSIM050, and a maximum value of 18 clones was used for simulations from LTXSIM051 to LTXSIM150, owing to the increased number of clones in the ground-truth instances. MEDICC2 (v2.0.4) was run with default parameters. For one simulated case (LTXSIM063), HATCHet2 did not complete within the 72 h time limit, and we excluded this case from the comparison.

#### Comparison metrics

##### HD matching clone comparison

To evaluate model performance using HD, we followed the following procedure. For each simulated clone, we calculated the HD between its ground-truth copy-number profile and the copy-number profiles of all clones predicted by the model. This allowed us to identify the most similar predicted clone for each true clone. After assigning a matching predicted clone and its corresponding HD to each true clone, we computed the mean of these distances. This final mean value represents how accurately the model retrieved the true copy-number states.

##### TVD

To evaluate model performance using TVD, we considered the output of each method (that is, copy-number states for each genomic segment and clone proportions associated with each such state) as probability distribution over discrete copy-number states and calculated the TVD between true and inferred distributions for each model. Owing to the high number of genomic segments in Tx simulations, TVD in Fig. 2c is averaged per patient before plotting.

#### Single-cell data processing

To evaluate the performance of ALPACA on real tumour data, we harnessed single-cell data acquired from 10x Genomics sequencing of a breast tumour and used CHISEL^35^ to estimate the copy number, reconstruct clonal architecture of the samples, generate ground-truth copy numbers and generate input data required by ALPACA. This dataset contained five samples (A–E). Sample A contained only normal cells and was excluded from this analysis. On the basis of the known number of cells within each clone, we calculated clone proportions in each sample and created a ground-truth reference for all clones excluding the MRCA, for which ground-truth data were not available. Next, we created confidence intervals for each segment and each sample by fitting the normal distribution to each segment’s fractional copy-number state with mean 0 and standard deviation of 0.1 to simulate noise. These data, together with the phylogenetic tree obtained from the original study^35^, were used as input to ALPACA. In this dataset, two clones dominate the tumour (JII and JIV), with the largest other clone corresponding to only approximately 4% of the sampled tumour (average across all samples, Extended Data Fig. 3e), and thus probably below the threshold of SNV clone detection using bulk sequencing using standard approaches^5^. Therefore, we excluded these small clones from the comparison.

### Identifying regions affected by extensive copy-number heterogeneity

To identify regions affected by extensive SCNA events, we calculated the coefficient of variation for each allele’s fractional copy number across all samples within each genomic segment. We then averaged these values to obtain the mean coefficient of variation for each segment. Next, we ranked the genomic segments within each tumour on the basis of their mean coefficient of variation and created four cohorts by progressively filtering out the top 5%, 15%, 25% or 50% of the genome with the highest variation.

### Mutation clustering concordance

We calculated clustering concordance scores for each pair of mutations, by checking whether mutations in both sets of results retained the same phylogenetic relationships in the default CONIPHER output compared to a cohort with 5%, 15%, 25% or 50% of the genome with the highest copy number variation removed. We classified each pair of mutations as: belonging to the same cluster; ancestor–descendant; descendant–ancestor; or parallel (not belonging to any other class). We scored each pair as concordant if the class of phylogenetic relationship was found to be the same in the full cohort and filtered cohort. The final score represents the fraction of concordant pairs out of all the possible pairs

### Preprocessing real data for input to ALPACA

#### Cohort

All biological analyses in Figs. 3–6 and Extended Data Figs. 1d, 2 and 5–10) were performed on the processed multi-sample bulk WES data from the recent longitudinal, prospective cohort TRACERx421 (refs. ^5,6^). For each tumour, the eligibility criteria for input to ALPACA included: allele-specific copy number computed for at least two primary tumour samples; a tumour phylogenetic tree computed from SNVs present across these same tumour samples, comprising at least two tumour clones (that is, at least an MRCA clone and one subclonal tree edge); and computed clone proportions of each clone on the phylogenetic tree in each sample. This resulted in *n* = 395 primary tumours^5^ and *n* = 126 paired primary–metastatic tumours with all of the correct inputs required for ALPACA.

#### Obtaining allele-specific fractional copy numbers per bulk tumour sample

The fractional copy numbers were further processed in the following way. We first identified clonal segments, by performing a statistical test to identify whether the fractional, allele-specific copy numbers were significantly different from their closest integer (*t*-test, *P*-value threshold of 0.0001). Segments with values significantly different from the nearest integers were considered subclonal. We further tested for evidence of allelic imbalance, by performing a statistical test to identify whether the fractional, allele-specific copy numbers for both alleles were different from one another in at least one sample (*t*-test, *P*-value threshold of 0.001). For each subclonal segment without evidence for allelic imbalance in any sample, we recalculated their total fractional copy number by summing the original fractional copy numbers for both alleles and assigning an integer copy number (closest integer to the original fractional copy number of respective allele) to allele B if the original fractional copy numbers for both alleles were larger than the closest integers for both alleles, or to allele A if they were less than the closest integer. The remaining allele (A or B, respectively) was assigned a fractional copy number calculated as the sum of its nearest integer and total fractional copy number minus the sum of closest integers for both original fractional copy numbers.

#### Correcting erroneous homozygous deletions

To prevent small clones being predicted as having copy number zero in both alleles for long stretches of the genome, the ALPACA method additionally implements constraints on the permitted number of homozygous deletions. Specifically, we introduce a threshold ${h}=1$ within each genomic segment $s\in \{1,\,...,{m}\}$, such that a clone can have a homozygous deletion only if: the clone or its descendants are present in a tumour sample that has a fractional copy number of less than the threshold $h$ in both alleles; and the genomic segment is shorter than 50 MB.

#### Obtaining a tumour phylogenetic tree

To obtain a single tumour phylogenetic tree per patient case in the TRACERx study, we used the published phylogenetic trees from the recent primary^5^ and primary–metastasis^6^ TRACERx421 cohorts. These tumour phylogenies were constructed from DNA-sequencing data using CONIPHER^21^, as described previously^5^. For tumour cases with multiple possible tumour phylogenetic trees, the tree with the lowest error was selected^21^.

#### Obtaining tumour clone proportions

For each tumour case, clone proportions of each tumour clone in each tumour sample were computed from the phylogenetic tree and mutation-cluster mean phylogenetic cancer cell fraction (PhyloCCF) values using the R function compute_subclone_proportions from the CONIPHER R package (v2.2.0)^21^. The input parameter value force_clonal_100 was set to be TRUE, to force clones whose distribution of associated mutation PhyloCCF values was not statistically distinct from that of the truncal cluster PhyloCCF values in certain tumour samples to have mean PhyloCCF = 1 in these samples. This ensured that when computing the clone proportions the clonal diversity was reduced and prevented noisy cancer cell fraction estimates resulting in samples harbouring clones with very small clone proportion.

### Computing clone ploidy

We used ALPACA to compute the clone ploidy for every clone from each tumour run through ALPACA in the TRACERx421 primary and primary–metastasis cohorts^5,6^. Clone ploidy was defined as follows: considering clone $i$ with total copy number ${c}_{s,i}={c}_{s,i}^{{\rm{A}}}+{c}_{s,i}^{{\rm{B}}}$ in genomic segment $s$, with segment size ${w}_{s}$, then the clone ploidy, ${p}_{i}$, was computed as the average clone copy number across all genomic segments, weighted by segment size:

$${p}_{i}=\frac{{\sum }_{s=1}^{m}{w}_{s}{c}_{s,i}}{{\sum }_{s=1}^{m}{w}_{s}}$$

### WGD detection using ALPACA

To identify WGD events, we compared copy-number states across the genome between each clone and its parent and grandparent. If the mean ratio, weighted by segment length, between the child clone and the parent clone was above 1.5, we classified the child clone as genome-doubled. If this ratio was below 1.5, but the ratio between the child and the grandparent was above 1.5 and the parent clone was not already classified as genome-doubled, we also classified the child clone as genome-doubled.

### Defining copy-number events on edges of the phylogenetic tree

#### Defining segment-specific copy-number events

For each tumour case and each genomic segment, we inferred the allele-specific copy number change on each edge of the phylogenetic tree on the basis of ALPACA’s output. Next, we applied the following criteria to classify whether a copy-number event had occurred on each edge. Considering an edge of the phylogenetic tree $({v}_{i},{v}_{j})\in E(T)$, and the corresponding allele-specific clone copy number of the parent clone ${{c}^{{\rm{A}}}}_{i}$, ${{c}^{{\rm{B}}}}_{i}$ and the child clone ${{c}^{{\rm{A}}}}_{j}$, ${{c}^{{\rm{B}}}}_{j}$, we define the following criteria for copy-number events, in which ${p}_{i}$ represents the clone ploidy of clone $i$:

Event name: criteria:

Copy-number gain: ${{c}^{{\rm{A}}}}_{j} > \,{{c}^{{\rm{A}}}}_{i}$ or ${{c}^{{\rm{B}}}}_{j} > {{c}^{{\rm{B}}}}_{i}$

Copy-number loss: ${{c}^{{\rm{A}}}}_{j} < \,{{c}^{{\rm{A}}}}_{i}$ or ${{c}^{{\rm{B}}}}_{j} < {{c}^{{\rm{B}}}}_{i}$

LOH: $${{c}^{{\rm{A}}}}_{i}\,\ne \,0\,\& \,{{c}^{{\rm{A}}}}_{j}\,=\,0$$ or $${{c}^{{\rm{B}}}}_{i}\,\ne \,0\,\& \,{{c}^{{\rm{B}}}}_{j}\,=\,0\,$$

Amplification: $${{c}^{{\rm{A}}}}_{j} > 2\times \,{{c}^{{\rm{A}}}}_{i}\,\& \,{{c}^{{\rm{A}}}}_{j}\ge 4\,\& \,{{c}^{{\rm{A}}}}_{j} > {p}_{j}$$

or $${{c}^{{\rm{B}}}}_{j} > 2\times \,{{c}^{{\rm{B}}}}_{i}\,\,{\rm{\& }}\,{{c}^{{\rm{B}}}}_{j}\ge 4\,\& \,{{c}^{{\rm{B}}}}_{j} > {p}_{j}$$

#### Defining chromosome-arm-level copy-number events

For each edge of the phylogenetic tree, we considered a whole chromosome arm to be affected by a copy-number event on the basis of the following classification, based on previous sample-level chromosome-arm copy-number calling analysis^10^:

Chromosome-arm event name: criteria

Copy-number gain: 90% of the chromosome arm is affected by gain, for either allele

Copy-number loss: 90% of the chromosome arm is affected by loss, for either allele

LOH: $\ge 90 \%$ of the chromosome arm is at copy number 0 in the child clone, but $\le 20 \%$ of the chromosome arm is at copy number 0 in the parent clone, for either allele

Amplification: $\ge 90 \%$ of the chromosome arm is amplified as per the criteria described above, for either allele.

#### Defining chromosome-arm-level copy-number events detectable with sample-level analysis

For each sample, we called an arm-level LOH event if at least 0.98 of the analysed genome had a fractional copy-number state below 0.5 for either allele.

### Event-ordering analysis

#### Identifying and assigning top frequently altered driver events to edges of the phylogenetic tree

To select driver SNVs and relevant copy-number events to include in the Bradley–Terry model for ordering driver events, we first classified the genomic driver events for each tumour case in the TRACERx421 primary and primary–metastasis cohorts^5,6^. These driver events included chromosome-arm amplifications and LOH events, focal (gene-level) amplifications and SNV driver events. We considered amplifications affecting oncogenes on the basis of a list collated from known copy-number drivers and known pan-cancer drivers as described in a previous study^5^. Oncogenes were considered to be amplified if the gene locus was completely contained within a copy-number segment called as amplified on an edge of the phylogenetic tree (using criteria described above). If a focal amplification was overlapping with a chromosome-arm amplification on an edge of the phylogenetic tree, only the chromosome-arm-level event was considered to have affected that edge of the tree. Then, including both clonal and subclonal events, we quantified the frequency of the number of tumours in which any such event was called. We selected the following events that were identified most frequently across the cohort to include in the ordering analysis, for LUAD and LUSC separately:

Event ID: number of top most frequent events selected for ordering analysis:

Chromosome-arm amplifications: 10

Chromosome-arm LOH events: 10

Subclonal chromosome-arm amplifications: 5

Subclonal chromosome-arm LOH events: 5

Focal amplifications (genes not part of an arm event): 10

Subclonal focal amplifications (genes not part of an arm event): 5

All SNVs classified as driver mutations as described in a previous study^26^: 10

Subclonal SNVs classified as driver mutations as described in a previous study^26^: 5

From this list of the top most frequent alterations, in LUAD and LUSC separately, we further subset to the events that were called as present in at least five tumours.

#### Specifying the event-ordering model

On the basis of these most frequent alterations, for LUAD and LUSC separately, we counted the number of tumours harbouring an alteration X from the top frequent alteration list occurring on an ancestral node, followed by any descendant node on the tree harbouring alteration Y from the top frequent alteration list (as depicted in Fig. 4a). If multiple descendant clones harboured the same descendant alteration Y to ancestral alteration X, we counted the ancestor–descendant relationship only once per tumour. These pairwise counts were entered into a Bradley–Terry model including a bias-reduced estimate, which was implemented using the BTm and update functions from the R package BradleyTerry2 (v1.1-2)^51^. When running the ordering analysis for the TRACERx421 primary–metastasis cohort^6^, we additionally considered metastatic ‘seeding’ as an event that could affect an edge of the phylogenetic tree, and therefore the metastatic seeding was ordered in the same way as other top most frequent events. Clones were labelled as being a seeding clone on the basis of the criteria described previously^6^.

### Expanded subclones

Expanded subclones were defined as non-MRCA tumour clones whose associated set of mutations seem clonal in at least one tumour sample^5^.

### Clone classification

We classified clones into classes as follows (Fig. 5a):

1. MRCA clones;
2. shared clones (clones with mutations shared between the primary and metastatic samples);
3. seeding clones (the most recent shared clones);
4. primary specific (clones with mutations detected only in primary samples); and
5. metastasis specific (clones with mutations detected only in metastatic samples).

### Computing number of SCNAs on a tree edge

ALPACA infers copy-number events for each segment independently; however, it is known that SCNAs can overlap multiple genomic segments^4,31,52,53^. Using the ALPACA output, we hence aimed to compute a summary statistic, called the number of SCNAs on a tree edge, $N$, to enumerate the actual number of copy-number events that occurred in the copy-number evolutionary tree inferred by ALPACA while taking into account neighbouring SCNAs. In brief, we computed $N$ as follows. Considering each allele and each chromosome separately, we counted the number of contiguous copy-number segments with a copy-number gain event of the same amplitude, and the number of contiguous segments with a copy-number loss event of the same amplitude, between the parent and the child node. The total number of copy-number events on this tree edge was then computed as the total sum of gains and losses on the tree edge, across all chromosomes, across both alleles. We note that in contrast to previous studies^4,52,53^, this method does not model the most parsimonious number of interval events required to transform the copy-number profile of the parent clone to that of the child clone. A mathematical description of the specific method used to compute the number of gains, losses and overall number of SCNAs on each edge is available in Supplementary Information section 4.

### Comparing rate of SNV and SCNA acquisition per tree edge

To compare the rate of SNVs and SCNAs acquisition on each tree edge, we considered the number of SNVs on each edge divided by the total sum of subclonal SNVs (that is, all mutations on subclonal edges of the tumour phylogeny) in the tumour across all tree edges, and compared this with the number of copy-number events (that is, number of SCNAs) on the same edge, divided by the total sum of subclonal copy-number events across all edges of the tumour. This analysis was run considering only subclones in both the TRACERx421 primary and primary–metastasis cohorts. Further, using the set of all subclones in the TRACERx421 primary–metastasis cohort, we fitted a linear mixed-effects model with the dependent variable being the normalized number of SCNAs, tumour as a random-effect explanatory variable, and the fixed-effect explanatory variables: normalized number of SNVs, histology and clone type (shared clones (reference level), seeding clones, primary clones or metastatic clones). Then, using the same set of all subclones in the TRACERx421 primary–metastasis cohort, we fitted a mixed-effects logistic regression model with the dependent binary variable of whether a clone was metastatic or not, tumour as a random-effect explanatory variable, and the fixed-effect explanatory variables number of SNVs, number of gains on the edge, number of losses on the edge and histology.

### Comparing SCNAs in seeding versus non-seeding clones

To identify specific copy-number events occurring on evolutionary trajectories leading to metastatic seeding, we considered only a subset of tumours from the TRACERx421 primary–metastasis cohort that satisfied the following conditions: the tumours had exclusively subclonal seeding; and the tumours had at least one non-seeding trajectory, which is defined as the tree path connecting the MRCA clone to a non-seeding primary clone: a primary clone that has no ancestors that are seeding (*n* = 71; Extended Data Fig. 8a). This set of tumours thus had both of the following tree paths present: paths connecting the MRCA clone to a subclonal seeding clone, and paths connecting the MRCA clone to a subclonal non-seeding primary clone.

For each oncogene in our collated list^5^, for each tumour, we performed a binary classification of whether a copy-number increase has occurred on a trajectory between the (non-seeding) MRCA and any seeding subclone, and a binary classification of whether a copy-number increase has occurred on a trajectory between the non-seeding MRCA and any non-seeding subclone (a copy-number decrease is considered for TSGs, respectively, and the specific copy-number events we considered are listed below). Then, for each oncogene event, we counted the number of tumours for which an increase affected subclonal seeding trajectories and the number of tumours for which an increase affected subclonal non-seeding trajectories. There are a different number of seeding and non-seeding clones in each tumour, and indeed a larger number of non-seeding clones overall, for each oncogene event. Hence, we tested whether the counts of tumours with events in seeding and non-seeding trajectories differed from a background count of the total number of such events affecting seeding and non-seeding trajectories summed across all non-driver genes.

We categorized ‘increase’ copy-number events at oncogene loci into: gains, gains >1 copy, amplifications. For TSG loci, we categorized ‘decrease’ copy-number events into: losses, losses >1 copy, LOH and LOH 0|1 (which describes the clone copy-number state of one allele with copy number 0, and the remaining allele with 1 copy).

### Calculating CCD

To calculate CCD for clones within the primary cohort, we first removed all of the clones exclusively found in the lymph nodes during the primary surgery, as these clones represent cells that have already migrated from the primary site. For the remaining clones, we concatenated the vectors of predicted clone copy-number states for alleles A and B along the genome for each clone, and computed the pairwise Euclidean distance of this vector between each pair of clones. Then, for each tumour case in the TRACERx421 primary cohort^5^, the maximum value distance between any two clones was chosen as the CCD score for that tumour.

### Clinical data and survival analysis

The clinical data were preprocessed in the same way as described in previous work^5^. Multivariable Cox model and Kaplan–Meier analyses were performed as described in previous work^5^. Covariates — age, stage, pack-years, histology, adjuvant and fraction of the aberrant genome with subclonal SCNAs computed on a sample level (termed ‘SCNA-ITH’ in ref. ^5^) — were processed as described in ref. ^5^ and were included as covariates in the Cox proportional hazards model.

### Statistical information

Statistical tests were performed using either R or Python (scipy v1.10.1).

All tests were two-sided, unless stated otherwise, and were carried out using paired or unpaired options as appropriate, unless stated otherwise. Hazard ratios and *P* values for the survival analysis were computed using the R Survival packages. For all plots involving statistical tests, all data points included are plotted or the number of data points included is specified in the figure legend.

When data is represented using box plots, the box represents the interquartile range with the median line. Whiskers denote the lowest and highest values within 1.5 times the interquartile range from the first and third quartiles, respectively.

### Reporting summary

Further information on research design is available in the Nature Portfolio Reporting Summary linked to this article.

## Online content

Any methods, additional references, Nature Portfolio reporting summaries, source data, extended data, supplementary information, acknowledgements, peer review information; details of author contributions and competing interests; and statements of data and code availability are available at 10.1038/s41586-025-09398-w.

## Supplementary information

Supplementary Information Supplementary Information sections 1–7 ((1) ALPACA’s model; (2) The ALPACA algorithm; (3) Implementation; (4) Computing the number of SCNAs on an edge; (5) Preparing input required by ALPACA; (6) Validation of the model selection procedure; (7) Validation of ALPACA assumptions), including Supplementary Figs. 1–9, Tables 1–4 and References.

Reporting Summary

## Data Availability

Processed data used in this study have been deposited at Zenodo, a platform maintained by CERN, the European Organization for Nuclear Research, serving for sharing and preserving data for scientific publications. The TRACERx primary and matched primary–metastasis processed data have been deposited at Zenodo at 10.5281/zenodo.7822002 (ref. ^54^). The single-cell study from the TRACERx PEACE study has been deposited at Zenodo at 10.5281/zenodo.13754279 (ref. ^55^). Processed data used in this publication have been deposited at Zenodo at 10.5281/zenodo.15519765 (ref. ^56^). The WES data (from the TRACERx study) used during this study have been deposited at the European Genome–Phenome Archive (EGA), which is hosted by the European Bioinformatics Institute and the Centre for Genomic Regulation, under the accession code EGAS00001006494; raw single-cell DNA-sequencing data used in this study from the patients enrolled in the TRACERx and PEACE studies have been deposited at the EGA under the accession code EGAD00001015411. Access is controlled by the TRACERx and PEACE data access committees. Details on how to apply for access are available via the EGA at https://ega-archive.org.

## Code availability

The code to run the ALPACA model is available via GitHub at https://github.com/McGranahanLab/ALPACA-model. The code to reproduce the figures is available via GitHub at https://github.com/McGranahanLab/ALPACA-paper. The end-to-end pipeline is available via GitHub at https://github.com/McGranahanLab/ALPACA-pipeline.
