# Supplementary material for: Clone copy number diversity is linked to survival in lung cancer
Source: Nature. 2025 Aug 13;646(8083):190–7. doi: 10.1038/s41586-025-09398-w (PMC12488491; doi:10.1038/s41586-025-09398-w)
Supplement: Supplementary file 1 — Supplementary Information sections 1–7 ((1) ALPACA’s model; (2) The ALPACA algorithm; (3) Implementation; (4) Computing the number of SCNAs on an edge; (5) Preparing input required by ALPACA; (6) Validation of the model selection procedure; (7) Validation of ALPACA assumptions), including Supplementary Figs. 1–9, Tables 1–4 and References. [file 41586_2025_9398_MOESM1_ESM.pdf]

nature portfolio

https://doi.org/10.1038/s41586-025-09398-w

Supplementary information

Clone copy number diversity is linked to survival in lung cancer

In the format provided by the authors and unedited

Clone specific chromosomal instability mediates metastatic routes and is linked to survival in lung cancer

Supplementary Information

Piotr Pawlik, Kristiana Grigoriadis,
Abigail Bunkum, Helena Coggan, Alexander M. Frankell, Carlos Martinez-Ruiz, Takahiro Karasaki,
Ariana Huebner, Andrew Rowan, Allan Hackshaw, Charles Swanton,
Simone Zaccaria*, Nicholas McGranahan*

April 2024

Contents

1 ALPACA's model **3**
- 1.1 Copy number model . . . 3
- 1.2 Tumour evolutionary model . . . 3
- 1.3 Model of sequencing observations . . . 4
- 1.4 Inference's objective . . . 4

2 The ALPACA algorithm **5**
- 2.1 Implementation of the ALPACA evolutionary model . . . 5
  - 2.1.1 Formulating copy number changes along each edge . . . 5
  - 2.1.2 Formulating persistent copy number loss . . . 6
  - 2.1.3 Formulating copy number fluctuation along a tree path . . . 7
- 2.2 Implementation of the model objective . . . 8
  - 2.2.1 Formulating the fractional copy number confidence intervals . . . 8
  - 2.2.2 Formulating the distance between predicted and observed fractional copy number estimates . . . 10
- 2.3 Complete ILP formulation . . . 11
- 2.4 Model selection . . . 13

3 Implementation **14**

4 Computing the number of SCNAs on an edge **14**

5 Preparing input required by ALPACA **15**
5.1 Preprocessing pipeline 15
5.2 Required inputs 16
5.2.1 Fractional copy-numbers for each sample and each genomic segment 16
5.2.2 Confidence intervals associated with each allele-specific fractional copy-number 16
5.2.3 Clone proportions table 16
5.2.4 Phylogenetic tree 17
5.2.5 Example input file structure 17
5.2.6 Running ALPACA 18

6 Validation of the model selection procedure **18**

7 Validation of ALPACA assumptions **21**
7.1 Estimating sufficient SNV acquisition rate with simulations 21
7.1.1 Background and aims 21
7.1.2 Methods 21
7.1.3 Results 23
7.2 Evaluating influence of SCNA affected genomic segments on SNV-based phylogeny reconstruction 24
7.3 Evaluating effects of using alternative CONIPHER trees 26
7.4 Evaluating effects of uncertainty in clone proportions derived from SNVs 28

1 ALPACA's model

ALPACA is an algorithm to infer clone-specific copy numbers and their evolution given three inputs that can be inferred by other existing methods from bulk DNA sequencing data of multiple samples obtained from the same tumour: (1) allele-specific fractional copy number, (2) the clone proportion of each tumour clone present in each tumour sample, and (3) the topology of the tumour phylogenetic tree representing the ancestral relationships of the inferred clones and their ancestors. In this section, we provide the model of copy number evolution underlying ALPACA and we define the objective of its inference.

1.1 Copy number model

We assume that Somatic Copy Number Alterations (SCNAs) partition all genomic positions into m copy number segments. We assume a tumour is composed of n distinct tumour clones. Each tumour clone $i \in \{1, ..., n\}$ in each genomic segment $s \in \{1, ..., m\}$ has a number $c^A_{s,i}$ of copies for allele A, and $c^B_{s,i}$ for allele B. For a normal diploid clone $i = 0$, we know that $c^A_{s,0} = c^B_{s,0} = 1$. Instead, for any tumour clone $i \in \{1, \ldots, n\}$ the copy numbers $c^A_{s,i}, c^B_{s,i}$ are unknown and ALPACA aims to infer them.

When sequencing k samples from a tumour, each sample comprises an unknown mixture of normal and tumour clones. Hence, we represent the proportion of each clone $i \in \{1, ..., n\}$ in each sample $r \in \{1, ..., k\}$ with $u_{i,r} \in [0, 1]$ such that $\sum_{i=1}^{n} u_{i,r} = 1$. Specifically, $u_{i,r} > 0$ indicates that clone i is present in sample r, and absent otherwise. Moreover, we define the tumour purity of each sample r as the proportion of all tumour clones, i.e., $1 - u_{0,r}$.

1.2 Tumour evolutionary model

All tumour clones are ancestrally related by a phylogenetic tree $T = (V, E)$, in which V represents the set of nodes and E the related set of edges, which also defines the *topology* of T. Specifically, each clone i is represented by a vertex v_i of the tree, i.e., $v_i \in V$. If tumour clone i is a parent of clone j, then (v_i, v_j) is an edge of T, i.e., $(v_i, v_j) \in E$. Without loss of generality, we assume that the most recent common ancestor of the tumour clones is clone $i = 1$. We also assume that the normal clone $i = 0$ is the root vertex of the tree $v_0 \in V$, such that $(v_0, v_1) \in E$.

Each node of the tree T is labelled by related copy numbers for all segments. Specifically, for each tumour clone $i \in \{1, ..., n\}$, the corresponding vertex $v_i \in V$ is labelled in each segment $s \in \{1, ..., m\}$ by the copy number $c^A_{s,i} \in \mathbb{N}$ for allele A, and $c^B_{s,i} \in \mathbb{N}$ for allele B. The normal root clone at vertex v_0 is labelled by $c^A_{s,0} = 1$ for allele A and $c^B_{s,0} = 1$ for allele B. Each edge of the tree $(v_i, v_j) \in E$ is thus labelled with events that are required to transform the copy numbers of clone i to those of clone j. In particular, similarly to previous copy-number evolutionary models (Nik-Zainal 2012; Ha 2014; Ha 2018), we model each copy number event for each segment independently as an event that either increases or decreases a copy number by one unit. As such, the label of each edge corresponds to $\sum_{s=1}^{m} |c^A_{s,i} - c^A_{s,j}| + |c^B_{s,i} - c^B_{s,j}|$ events, since $|c^A_{s,i} - c^A_{s,j}|$ events are required for allele A of each segment s and $|c^B_{s,i} - c^B_{s,j}|$ for allele B.

Given a fixed topology for a tree T, an arbitrarily high number of copy-number labelings for T can be defined in general. Therefore, we introduce an evolutionary model based on three realistic assumptions that define the best copy-number-labelled tree T to represent copy-number evolution. First, we assume loss-of-heterozygosity (LOH) events are irreversible, i.e., if all the copies of one allele in any segment are lost these cannot be regained by any descendent. Specifically, for any segment $s \in \{1, ..., m\}$ in any edge $(v_i, v_j) \in E$ it holds that $c^A_{s,i} = 0 \implies c^A_{s,j} = 0$ and $c^B_{s,i} = 0 \implies c^B_{s,j} = 0$. Second, ALPACA allows both amplifications and

deletions to occur along every evolutionary path, however, to prevent overfitting, changes in amplifications and deletions along each path can occur at most once. Last, under the assumption of parsimony, we define the tree with the least number of copy-number changes to be the more likely explanation of copy number evolution, similarly to previous approaches (Zaccaria et al. 2018).

1.3 Model of sequencing observations

The copy numbers of each clone are not observed from DNA sequencing data, but existing methods (Van Loo 2010; Favero 2015; Shen et al. 2016; Watkins et al. 2023; Carter 2012) allow the inference of fractional copy numbers for each allele in each tumour sample, which correspond to a weighted average of the copy numbers for all clones present in a sample. Formally, we define the **true** fractional copy number $f^{A}_{s,r} \in \mathbb{R}^{\geq 0}$ of allele A and the fractional copy number $f^{B}_{s,r} \in \mathbb{R}^{\geq 0}$ of allele B as the average copy number of all cells in each tumour sample $r \in \{1, ..., k\}$ for each segment $s \in \{1, ..., m\}$, i.e., $\sum_{i=1}^{n} c^{A}_{s,i} u_{i,r}$, and $\sum_{i=1}^{n} c^{B}_{s,i} u_{i,r}$ respectively. Existing methods do not infer $f^{A}_{s,r}$ and $f^{B}_{s,r}$ exactly but rather an estimate of these values. Moreover, they provide estimates of their upper bounds, $f^{A,+}_{s,r} \in \mathbb{R}^{\geq 0}$ and $f^{B,+}_{s,r} \in \mathbb{R}^{\geq 0}$ of $\sum_{i=1}^{n} c^{A}_{s,i} u_{i,r}$ and $\sum_{i=1}^{n} c^{B}_{s,i} u_{i,r}$ for each sample r and genomic segment s, as well as estimates of their lower bound, $f^{A,-}_{s,r} \in \mathbb{R}^{\geq 0}$ and $f^{B,-}_{s,r} \in \mathbb{R}^{\geq 0}$ of $\sum_{i=1}^{n} c^{A}_{s,i} u_{i,r}$ and $\sum_{i=1}^{n} c^{B}_{s,i} u_{i,r}$ for each sample r and genomic segment s. Overall, we thus expect that $f^{A}_{s,r} \in [f^{A,-}_{s,r}, f^{A,+}_{s,r}]$ and $f^{B}_{s,r} \in [f^{B,-}_{s,r}, f^{B,+}_{s,r}]$. As such, the copy numbers of the clones present in all sequenced samples can be inferred by solving a factorization problem in which fractional copy numbers are deconvolved into clone-specific copy numbers and the related proportions in each sample, similar to other works (Zaccaria et al. 2020; Myers et al. 2023; Ha 2014; Oesper et al. 2013; McPherson et al. 2017).

1.4 Inference's objective

The hypothesis underlying ALPACA is that copy-number inference from fractional copy numbers can be improved by integrating other two other data that can be inferred by existing methods, in addition to the estimated fractional copy numbers: the topology of the tree T defined by the related edges E and the proportion $u_{i,r}$ of each clone i in each sample r. In fact, E and $u_{0,r}, \ldots, u_{n,r}$ can be inferred using somatic single-nucleotide variants (SNVs) by other existing methods, such as CONIPHER (Grigoriadis et al. 2024), Canopy (Jiang et al. 2016), LICHeE (Popic et al. 2015), and others (Malikic et al. 2015; El-Kebir et al. 2016; Wintersinger et al. 2022; Myers et al. 2019). Using fixed clone proportions is expected to improve the performance of copy-number inference because it simplifies the complex copy-number deconvolution problem into an easier linear optimisation problem. Moreover, phylogenetic methods that infer T do not only infer extant tumour clones (i.e., clones that are present in at least one tumour sample), but they are also able to infer ancestral clones that might be extinct and not been observed in the sequenced samples. Therefore, while existing deconvolution methods can only infer the copy numbers of extant tumour clones, ALPACA is able to infer the copy numbers of both extant and ancestral clones by integrating T. Overall, ALPACA thus aims to infer the unknown copy-numbers of each clone in a tree T with a given topology, i.e., given the set of edges E, and given the fractional copy number in each sequenced tumour sample.

Formally, ALPACA's algorithm is framed as a linear optimisation problem for each segment $s \in \{1, ..., m\}$ aiming to find the copy numbers $c^{A}_{s,i}$ and $c^{B}_{s,i}$ for all clones $i \in \{1, ..., n\}$ that satisfy the following conditions:

1. Given $f^{A,+}_{s,r}, f^{B,+}_{s,r}, f^{A,-}_{s,r}, f^{B,-}_{s,r}, u_{i,r}$ for each segment s in each sample r and the edges E of T, find $c^{A}_{s,i}$ and $c^{B}_{s,i}$ for every tumour clone i that defines a parsimonious and constrained copy-number labeling of T as defined in Section 1.2.

2. Among the copy numbers that satisfy the first property, find $c^A_{s,i}$ and $c^B_{s,i}$ that minimize the absolute difference between the predicted and expected fractional copy number, $|\hat{f}^A_{s,r} - \sum_{i=1}^{n} c^A_{s,i} u_{i,r}|$ and $|\hat{f}^B_{s,r} - \sum_{i=1}^{n} c^B_{s,i} u_{i,r}|$, where the expected fractional copy numbers $\hat{f}^A_{s,r}, \hat{f}^B_{s,r}$ are defined as the mean of the upper and lower bound estimates $f^{A,+}_{s,r}$, $f^{A,-}_{s,r}$ and $f^{B,+}_{s,r}$, $f^{B,-}_{s,r}$.

ALPACA solves this optimization problem using an algorithm based on a mixed-integer linear optimization formulation and model selection approach defined in the following Section 2.

2 The ALPACA algorithm

In order to implement the optimisation problem stated above as an MIP, we define the following additional variables and constraints.

2.1 Implementation of the ALPACA evolutionary model

2.1.1 Formulating copy number changes along each edge

We model the allele-specific copy number of each clone $i \in \{1, ..., n\}$ in each segment $s \in \{1, ..., m\}$ as vectors $c^A_{s,i} \in \mathbb{N}$ and $c^B_{s,i} \in \mathbb{N}$, and diploid root clone copy numbers $c^A_{s,0} = 0$ and $c^B_{s,0} = 0$ as described above. Specifically, we constrain:

$$c^A_{s,0} = 1 \qquad \forall s \in \{1, ..., m\} \tag{1}$$

$$c^B_{s,0} = 1 \qquad \forall s \in \{1, ..., m\} \tag{2}$$

To model the copy number changes that occur between clones, we first define non-negative integer variables $a^A_{s,i,j}, a^B_{s,i,j} \in \mathbb{N}$, and $d^A_{s,i,j}, d^B_{s,i,j} \in \mathbb{N}$ that represent the copy number amplifications and deletions, respectively, affecting edge $(v_i, v_j) \in E$ of the phylogenetic tree T in segment s. Then we have:

$$c^A_{s,i} + a^A_{s,i,j} = c^A_{s,j} + d^A_{s,i,j} \qquad \forall s \in \{1, ..., m\}, \forall (v_i, v_j) \in E \tag{3}$$

$$c^B_{s,i} + a^B_{s,i,j} = c^B_{s,j} + d^B_{s,i,j} \qquad \forall s \in \{1, ..., m\}, \forall (v_i, v_j) \in E \tag{4}$$

We next define the binary variables $\bar{a}^A_{s,i,j} \in \{0, 1\}$ and $\bar{d}^A_{s,i,j} \in \{0, 1\}$ to indicate whether an amplification, or deletion, respectively, occurred on edge $(v_i, v_j) \in E$ in segment s, for allele A, and similarly for allele B. That is,

$$\bar{a}^A_{s,i,j} = \begin{cases} 1, & \text{if } c^A_{s,i} < c^A_{s,j} \\ 0, & \text{otherwise} \end{cases} \tag{5}$$

$$\bar{a}^B_{s,i,j} = \begin{cases} 1, & \text{if } c^B_{s,i} < c^B_{s,j} \\ 0, & \text{otherwise} \end{cases} \tag{6}$$

$$\bar{d}^A_{s,i,j} = \begin{cases} 1, & \text{if } c^A_{s,i} > c^A_{s,j} \\ 0, & \text{otherwise} \end{cases} \tag{7}$$

$$\bar{d}^B_{s,i,j} = \begin{cases} 1, & \text{if } c^B_{s,i} > c^B_{s,j} \\ 0, & \text{otherwise} \end{cases} \tag{8}$$

To model variables $\bar{a}^A_{s,i,j}, \bar{d}^A_{s,i,j}, \bar{a}^B_{s,i,j}, \bar{d}^B_{s,i,j}$ using linear constraints, we define $U >> 0, U \in \mathbb{R}$ such that for each segment s:

$$a^A_{s,i,j} \leq U * \bar{a}^A_{s,i,j} \qquad \forall (v_i, v_j) \in E \tag{9}$$
$$a^B_{s,i,j} \leq U * \bar{a}^B_{s,i,j} \qquad \forall (v_i, v_j) \in E \tag{10}$$
$$a^A_{s,i,j} \geq \bar{a}^A_{s,i,j} \qquad \forall (v_i, v_j) \in E \tag{11}$$
$$a^B_{s,i,j} \geq \bar{a}^B_{s,i,j} \qquad \forall (v_i, v_j) \in E \tag{12}$$

We constrain each edge and each allele to only have a copy number change either up or down, and model this with the following linear constraints:

$$\bar{a}^A_{s,i,j} + \bar{d}^A_{s,i,j} \leq 1 \qquad \forall s \in \{1, ..., m\}, \forall (v_i, v_j) \in E \tag{13}$$
$$\bar{a}^B_{s,i,j} + \bar{d}^B_{s,i,j} \leq 1 \qquad \forall s \in \{1, ..., m\}, \forall (v_i, v_j) \in E \tag{14}$$

Finally, under an assumption of parsimony, ALPACA constrains the total number of copy number changes across all edges $(v_i, v_j) \in E$ of the phylogenetic tree for segment s, by some maximum real value $\lambda_s \in \mathbb{R}$, such that for each segment $s \in \{1, ..., m\}$, $\lambda_s \in \mathbb{N}$ is an upper bound for the sum of all the copy number changes across all the edges of the tree in segment s, for alleles A and B, i.e.:

$$\sum_{(v_i, v_j) \in E} \left(|c^A_{s,i} - c^A_{s,j}| + |c^B_{s,i} - c^B_{s,j}| \right) \leq \lambda_s \tag{15}$$

Hence, to model this in our ILP, we implement the following constraint:

$$\sum_{(v_i, v_j) \in E} \left(a^A_{s,i,j} + d^A_{s,i,j} + a^B_{s,i,j} + d^B_{s,i,j} \right) \leq \lambda_s \tag{16}$$

In practice, the parameter λ_s is varied in a model selection step described in further detail in section 2.4, in which the final value of λ_s is selected to result in a number of copy number events on the tree that does not overfit the data.

2.1.2 Formulating persistent copy number loss

The ALPACA model states that a copy number cannot increase from state 0 along any edge $(v_i, v_j) \in E$ in any segment s, that is,

$$c^A_{s,i} = 0 \implies c^A_{s,j} = 0 \qquad \forall s \in \{1, ..., m\}, \forall (v_i, v_j) \in E \tag{17}$$
$$c^B_{s,i} = 0 \implies c^B_{s,j} = 0 \qquad \forall s \in \{1, ..., m\}, \forall (v_i, v_j) \in E \tag{18}$$

We enforce this in the ALPACA algorithm by including the following constraint:

$$c^A_{s,i} \geq \bar{a}^A_{s,i,j} \qquad \forall s \in \{1, ..., m\}, \forall (v_i, v_j) \in E \tag{19}$$
$$c^B_{s,i} \geq \bar{a}^B_{s,i,j} \qquad \forall s \in \{1, ..., m\}, \forall (v_i, v_j) \in E \tag{20}$$

The above constraint ensures that if a positive copy number change (i.e. an amplification) occurred along edge $(v_i, v_j) \in E$, then $\bar{a}^A_{s,i,j} \geq 1 \implies c^A_{s,i} \geq 1$ (and allele B, respectively), that is, the parent copy number must be non-zero.

2.1.3 Formulating copy number fluctuation along a tree path

As introduced above, the ALPACA model assumes that under an assumption of parsimony, copy number changes are predominantly limited to either amplifications or deletions along a tree path from the diploid root node of the tree v_0, to a leaf node of the tree $l \in L$, where L is the set of leaf nodes of the tree. Specificaly, we define set of leaf nodes of the tree as $L = \{v_l \in V : l \in \{0, 1, ..., n\}, \exists i \in \{0, 1, ..., n\}$ s.t. $(v_i, v_l) \in E, \nexists j \in \{0, 1, ..., n\}$ s.t. $(v_l, v_j) \in E\}$. We further define the set of tree paths of tree T, P as the set of sequences of vertices that connect edges from the root vertex of the tree v_0 to a leaf vertex $v_l, l \in L$. That is, $P = \{p_l = (v_0, v_1, ..., v_l) : v_i \in V, l \in L, (v_i, v_{i+1}) \in E\}$. ALPACA restricts the total amount of copy number fluctuation along a tree path from the diploid root vertex of the tree v_0 to a leaf $v_l, l \in L$ whereby for each segment $s \in \{1, ..., m\}$, for each tree path $p_l \in P$, we constrain there to be maximally one edge with a copy number change in the opposite direction to the changes occurring on edges along the rest of the path..

In order to model this in the MIP, we define integer variables $a^A_{s,l}, a^B_{s,l} \in \mathbb{N}$ and $d^A_{s,l}, d^B_{s,l} \in \mathbb{N}$ that are the total number of allele-specific amplification and deletion events, respectively, affecting all edges on path $p_l \in P$, for segment s:

$$a^A_{s,l} = \sum_{\substack{(v_i,v_j)\in E \\ v_i,v_j \in p_l}} a^A_{s,i,j} \tag{21}$$

$$a^B_{s,l} = \sum_{\substack{(v_i,v_j)\in E \\ v_i,v_j \in p_l}} a^B_{s,i,j} \tag{22}$$

$$d^A_{s,l} = \sum_{\substack{(v_i,v_j)\in E \\ v_i,v_j \in p_l}} d^A_{s,i,j} \tag{23}$$

$$d^B_{s,l} = \sum_{\substack{(v_i,v_j)\in E \\ v_i,v_j \in p_l}} d^B_{s,i,j} \tag{24}$$

We also define binary variables, $\hat{a}^A_{s,l}, \hat{a}^B_{s,l} \in \{0, 1\}$ and $\hat{d}^A_{s,l}, \hat{d}^B_{s,l} \in \{0, 1\}$ to model whether at least two amplifications, or deletions, respectively occurred on path p_l in segment s. That is,

$$\hat{a}^A_{s,l} = \begin{cases} 1 & \text{if } a^A_{s,l} \geq 2 \\ 0 & a^A_{s,l} \in \{0, 1\} \end{cases} \qquad \forall s \in \{1, ..., m\} \tag{25}$$

$$\hat{d}^A_{s,l} = \begin{cases} 1 & \text{if } d^A_{s,l} \geq 2 \\ 0 & d^A_{s,l} \in \{0, 1\} \end{cases} \qquad \forall s \in \{1, ..., m\} \tag{26}$$

$$\hat{a}^B_{s,l} = \begin{cases} 1 & \text{if } a^B_{s,l} \geq 2 \\ 0 & a^B_{s,l} \in \{0, 1\} \end{cases} \qquad \forall s \in \{1, ..., m\} \tag{27}$$

$$\hat{d}^B_{s,l} = \begin{cases} 1 & \text{if } d^B_{s,l} \geq 2 \\ 0 & d^B_{s,l} \in \{0, 1\} \end{cases} \qquad \forall s \in \{1, ..., m\} \tag{28}$$

$$\tag{29}$$

These variables are modelled using the following linear constraints, defining $H >> 0, H \in \mathbb{R}$:

$$a^A_{s,l} \geq 2 - H + H * \hat{a}^A_{s,l} \tag{30}$$

$$a^B_{s,l} \geq 2 - H + H * \hat{a}^B_{s,l} \tag{31}$$

$$a^A_{s,l} \leq 1 + H * \hat{a}^A_{s,l} \tag{32}$$
$$a^B_{s,l} \leq 1 + H * \hat{a}^B_{s,l} \tag{33}$$
$$d^A_{s,l} \geq 2 - H + H * \hat{a}^A_{s,l} \tag{34}$$
$$d^B_{s,l} \geq 2 - H + H * \hat{a}^B_{s,l} \tag{35}$$
$$d^A_{s,l} \leq 1 + H * \hat{a}^A_{s,l} \tag{36}$$
$$d^B_{s,l} \leq 1 + H * \hat{a}^B_{s,l} \tag{37}$$

Hence, in order to allow maximally one edge to have a copy number change in the opposite direction to the changes occurring on edges along the rest of the path, we implement the following linear constraint:

$$\hat{a}^A_{s,l} + \hat{d}^A_{s,l} \leq 1 \qquad \forall s \in \{1,...,m\}, \forall p_l \in P \tag{38}$$
$$\hat{a}^B_{s,l} + \hat{d}^B_{s,l} \leq 1 \qquad \forall s \in \{1,...,m\}, \forall p_l \in P \tag{39}$$

2.2 Implementation of the model objective

As described above, for each tumour sample $r \in \{1,...,k\}$, in each genomic segment $s \in \{1,...,m\}$ we model the clone proportions of every tumour clone $i \in \{1,...,n\}$, $u_{i,r} \in [0,1]$. Then, the average copy number (i.e. 'fractional' copy number) of all cells in the sample r is $\sum_{i=1}^n c^A_{s,i}u_{i,r}$, and $\sum_{i=1}^n c^B_{s,i}u_{i,r}$, for alleles A and B, respectively. We observe estimates of these fractional copy number values $f^A_{s,r} \in \mathbb{R}^{\geq 0}$ and $f^B_{s,r} \in \mathbb{R}^{\geq 0}$, as well as estimates of the uncertainty of the upper bound estimates, $f^{A,+}_{s,r} \in \mathbb{R}^{\geq 0}$ and $f^{B,+}_{s,r} \in \mathbb{R}^{\geq 0}$, and lower bound estimates, $f^{A,-}_{s,r} \in \mathbb{R}^{\geq 0}$ and $f^{B,-}_{s,r} \in \mathbb{R}^{\geq 0}$ for each sample r and genomic segment s.

ALPACA aims to minimize two hierarchical model objectives. Model objective (1) is to find $c^A_{s,i}$ and $c^B_{s,i}$ $\forall i \in \{1,...,n\}, \forall s \in \{1,...,m\}$ that minimizes the number of samples for which $\sum_{i=1}^n c^A_{s,i}u_{i,r} \notin [f^{A,-}_{s,r}, f^{A,+}_{s,r}]$ or $\sum_{i=1}^n c^B_{s,i}u_{i,r} \notin [f^{B,-}_{s,r}, f^{B,+}_{s,r}]$. Then among these solutions, model objective (2) is to find $c^A_{s,i}$ and $c^B_{s,i}$ that minimize the absolute difference between the predicted and observed fractional copy number values, $|f^A_{s,r} - \sum_{i=1}^n c^A_{s,i}u_{i,r}|$ and $|f^B_{s,r} - \sum_{i=1}^n c^B_{s,i}u_{i,r}|$.

2.2.1 Formulating the fractional copy number confidence intervals

To formulate our first model objective, we introduce the following additional variables. We first define binary variables $z^{A,+}_{s,r} \in \{0,1\}$ and $z^{B,+}_{s,r} \in \{0,1\}$, to indicate whether the predicted fractional copy numbers for each segment s and each sample r lie above the the upper bound estimates, $f^{A,+}_{s,r}$ and $f^{B,+}_{s,r}$. That is, we wish to model:

$$z^{A,+}_{s,r} = \begin{cases} 1, & \text{if } \sum_{i=1}^n c^A_{s,i}u_{i,r} \geq f^{A,+}_{s,r} \\ 0, & \text{otherwise} \end{cases} \tag{40}$$

$$z^{B,+}_{s,r} = \begin{cases} 1, & \text{if } \sum_{i=1}^n c^B_{s,i}u_{i,r} \geq f^{B,+}_{s,r} \\ 0, & \text{otherwise} \end{cases} \tag{41}$$

To model variables $z^{A,+}_{s,r}$ and $z^{B,+}_{s,r}$ using linear constraints, we define $M >> 0, M \in \mathbb{R}$ such that:

$$\sum_{i=1}^n c^A_{s,i}u_{i,r} \geq f^{A,+}_{s,r} - M + M * z^{A,+}_{s,r} \tag{42}$$

$$\sum_{i=1}^{n} c_{s,i}^{A} u_{i,r} \leq f_{s,r}^{A,+} + M * z_{s,r}^{A,+} \tag{43}$$

$$\sum_{i=1}^{n} c_{s,i}^{B} u_{i,r} \geq f_{s,r}^{B,+} - M + M * z_{s,r}^{B,+} \tag{44}$$

$$\sum_{i=1}^{n} c_{s,i}^{B} u_{i,r} \leq f_{s,r}^{B,+} + M * z_{s,r}^{B,+} \tag{45}$$

Similarly, we define binary variables $z_{s,r}^{A,-} \in \{0,1\}$ and $z_{s,r}^{B,-} \in \{0,1\}$, to indicate whether the predicted fractional copy numbers for each segment s and each sample r lie below the the lower bound estimates, $f_{s,r}^{A,-}$ and $f_{s,r}^{B,-}$. That is, we wish to model:

$$z_{s,r}^{A,-} = \begin{cases} 1, & \text{if } \sum_{i=1}^{n} c_{s,i}^{A} u_{i,r} \leq f_{s,r}^{A,-} \\ 0, & \text{otherwise} \end{cases} \tag{46}$$

$$z_{s,r}^{B,-} = \begin{cases} 1, & \text{if } \sum_{i=1}^{n} c_{s,i}^{B} u_{i,r} \leq f_{s,r}^{B,-} \\ 0, & \text{otherwise} \end{cases} \tag{47}$$

The, to model variables $z_{s,r}^{A,-}$ and $z_{s,r}^{B,-}$ using linear constraints:

$$\sum_{i=1}^{n} c_{s,i}^{A} u_{i,r} \leq f_{s,r}^{A,-} + M - M * z_{s,r}^{A,l} \tag{48}$$

$$\sum_{i=1}^{n} c_{s,i}^{A} u_{i,r} \geq f_{s,r}^{A,-} - M * z_{s,r}^{A,-} \tag{49}$$

$$\sum_{i=1}^{n} c_{s,i}^{B} u_{i,r} \leq f_{s,r}^{B,-} + M - M * z_{s,r}^{B,l} \tag{50}$$

$$\sum_{i=1}^{n} c_{s,i}^{B} u_{i,r} \geq f_{s,r}^{B,-} - M * z_{s,r}^{B,-} \tag{51}$$

Next, we define binary variables $z_{s,r}^{A} \in \{0,1\}$ and $z_{s,r}^{B} \in \{0,1\}$ that indicates whether prediction $\sum_{i=1}^{n} c_{s,i}^{A} u_{i,r}$ lies above $f_{s,r}^{A,+}$, or below $f_{s,r}^{A,-}$ for segment s in sample r, and similarly for allele B. That is,

$$z_{s,r}^{A} = \begin{cases} 1, & \text{if } \sum_{i=1}^{n} c_{s,i}^{A} u_{i,r} \notin [f_{s,r}^{A,-}, f_{s,r}^{A,+}] \\ 0, & \text{otherwise} \end{cases} \tag{52}$$

$$z_{s,r}^{B} = \begin{cases} 1, & \text{if } \sum_{i=1}^{n} c_{s,i}^{B} u_{i,r} \notin [f_{s,r}^{B,-}, f_{s,r}^{B,+}] \\ 0, & \text{otherwise} \end{cases} \tag{53}$$

We model these with the following constraints:

$$z_{s,r}^{A} \geq z_{s,r}^{A,-} \tag{54}$$

$$z_{s,r}^{B} \geq z_{s,r}^{A,-} \tag{55}$$

$$z_{s,r}^{A} \geq z_{s,r}^{A,+} \tag{56}$$

$$z_{s,r}^{B} \geq z_{s,r}^{A,+} \tag{57}$$

$$z_{s,r}^{A} \leq z_{s,r}^{A,+} + z_{s,r}^{A,-} \tag{58}$$

$$z^B_{s,r} \leq z^{A,+}_{s,r} + z^{A,-}_{s,r} \tag{59}$$

Hence, for model objective (1): the sum of samples $r \in \{1, ..., k\}$ in which the predicted fractional copy number values lie $\sum_{i=1}^n c^A_{s,i} u_{i,r}$ and $\sum_{i=1}^n c^B_{s,i} u_{i,r}$ lie outside $[f^{A,-}_{s,r}, f^{A,+}_{s,r}]$ and $[f^{B,-}_{s,r}, f^{B,+}_{s,r}]$, can be expressed as

$$\sum_{r=1}^{k} z^A_{s,r} + z^B_{s,r} \qquad \forall s \in \{1, ..., m\} \tag{60}$$

2.2.2 Formulating the distance between predicted and observed fractional copy number estimates

For our second model objective, we define variables $d^A_{s,r}$ and $d^B_{s,r}$ that represent the total predicted L_1 distance between the predicted ($\sum_{i=1}^n c^A_{s,i} u_{i,r}$ and $\sum_{i=1}^n c^B_{s,i} u_{i,r}$) and observed ($f^A_{s,r}$ and $f^B_{s,r}$) fractional copy numbers across all samples $r \in \{1, ..., k\}$, for each segment $s \in \{1, ..., m\}$. We model $d^A_{s,r}$ and $d^B_{s,r}$ using the following linear constraints:

$$d^A_{s,r} \geq \sum_{i=1}^{n} c^A_{s,i} u_{i,r} - f^A_{s,r} \tag{61}$$

$$d^B_{s,r} \geq \sum_{i=1}^{n} c^B_{s,i} u_{i,r} - f^B_{s,r} \tag{62}$$

$$-d^A_{s,r} \leq \sum_{i=1}^{n} c^A_{s,i} u_{i,r} - f^A_{s,r} \tag{63}$$

$$-d^B_{s,r} \leq \sum_{i=1}^{n} c^B_{s,i} u_{i,r} - f^B_{s,r} \tag{64}$$

Hence, for model objective (2): the total distance between predicted and observed fractional copy number estimates across all tumour samples $r \in \{1, ..., k\}$ can be expressed as:

$$\sum_{r=1}^{k} (d^A_{s,r} + d^B_{s,r}) \qquad \forall s \in \{1, ..., m\} \tag{65}$$

2.3 Complete ILP formulation

Hence, the complete ILP formulation of ALPACA can be expressed as follows.
For fixed $U, M, H, \lambda_s \in \mathbb{R}$:

$$\text{Objective 1: } \min \sum_{r=1}^{k} z_{s,r}^{A} + z_{s,r}^{B} \qquad \forall s \in \{1, ..., m\} \tag{66}$$

$$\text{Objective 2: } \min \sum_{r=1}^{k} (d_{s,r}^{A} + d_{s,r}^{B}) \qquad \forall s \in \{1, ..., m\} \tag{67}$$

$$c_{s,i}^{A} \in \mathbb{N}^{+} \qquad \forall s \in \{1, ..., m\}, \forall i \in \{0, 1, ..., n\} \tag{68}$$

$$c_{s,i}^{B} \in \mathbb{N}^{+} \qquad \forall s \in \{1, ..., m\}, \forall i \in \{0, 1, ..., n\} \tag{69}$$

$$c_{s,0}^{A} = 1 \qquad \forall s \in \{1, ..., m\} \tag{70}$$

$$c_{s,0}^{B} = 1 \qquad \forall s \in \{1, ..., m\} \tag{71}$$

$$a_{s,i}^{A} \in \mathbb{N}^{+} \qquad \forall s \in \{1, ..., m\}, \forall i \in \{0, 1, ..., n\} \tag{72}$$

$$a_{s,i}^{B} \in \mathbb{N}^{+} \qquad \forall s \in \{1, ..., m\}, \forall i \in \{0, 1, ..., n\} \tag{73}$$

$$d_{s,i}^{A} \in \mathbb{N}^{+} \qquad \forall s \in \{1, ..., m\}, \forall i \in \{0, 1, ..., n\} \tag{74}$$

$$d_{s,i}^{B} \in \mathbb{N}^{+} \qquad \forall s \in \{1, ..., m\}, \forall i \in \{0, 1, ..., n\} \tag{75}$$

$$\bar{a}_{s,i,j}^{A} \in \{0, 1\} \qquad \forall s \in \{1, ..., m\}, \forall (v_i, v_j) \in E \tag{76}$$

$$\bar{a}_{s,i,j}^{B} \in \{0, 1\} \qquad \forall s \in \{1, ..., m\}, \forall (v_i, v_j) \in E \tag{77}$$

$$a_{s,l}^{A} \in \mathbb{N}^{+} \qquad \forall s \in \{1, ..., m\}, \forall p_l \in P \tag{78}$$

$$a_{s,l}^{B} \in \mathbb{N}^{+} \qquad \forall s \in \{1, ..., m\}, \forall p_l \in P \tag{79}$$

$$d_{s,l}^{A} \in \mathbb{N}^{+} \qquad \forall s \in \{1, ..., m\}, \forall p_l \in P \tag{80}$$

$$d_{s,l}^{B} \in \mathbb{N}^{+} \qquad \forall s \in \{1, ..., m\}, \forall p_l \in P \tag{81}$$

$$\hat{a}_{s,l}^{A} \in \{0, 1\} \qquad \forall s \in \{1, ..., m\}, \forall p_l \in P \tag{82}$$

$$\hat{a}_{s,l}^{B} \in \{0, 1\} \qquad \forall s \in \{1, ..., m\}, \forall p_l \in P \tag{83}$$

$$\hat{d}_{s,l}^{A} \in \{0, 1\} \qquad \forall s \in \{1, ..., m\}, \forall p_l \in P \tag{84}$$

$$\hat{d}_{s,l}^{B} \in \{0, 1\} \qquad \forall s \in \{1, ..., m\}, \forall p_l \in P \tag{85}$$

$$z_{s,r}^{A,+} \in \{0, 1\} \qquad \forall s \in \{1, ..., m\}, \forall r \in \{1, ..., k\} \tag{86}$$

$$z_{s,r}^{B,+} \in \{0, 1\} \qquad \forall s \in \{1, ..., m\}, \forall r \in \{1, ..., k\} \tag{87}$$

$$z_{s,r}^{A,-} \in \{0, 1\} \qquad \forall s \in \{1, ..., m\}, \forall r \in \{1, ..., k\} \tag{88}$$

$$z_{s,r}^{B,-} \in \{0, 1\} \qquad \forall s \in \{1, ..., m\}, \forall r \in \{1, ..., k\} \tag{89}$$

$$z_{s,r}^{A} \in \{0, 1\} \qquad \forall s \in \{1, ..., m\}, \forall r \in \{1, ..., k\} \tag{90}$$

$$z_{s,r}^{B} \in \{0, 1\} \qquad \forall s \in \{1, ..., m\}, \forall r \in \{1, ..., k\} \tag{91}$$

$$c_{s,i}^{A} + a_{s,i,j}^{A} = c_{s,j}^{A} + d_{s,i,j}^{A} \qquad \forall s \in \{1, ..., m\}, \forall (v_i, v_j) \in E \tag{92}$$

$$c_{s,i}^{B} + a_{s,i,j}^{B} = c_{s,j}^{B} + d_{s,i,j}^{B} \qquad \forall s \in \{1, ..., m\}, \forall (v_i, v_j) \in E \tag{93}$$

$$a_{s,i,j}^{A} \leq U * \bar{a}_{s,i,j}^{A} \qquad \forall s \in \{1, ..., m\}, \forall (v_i, v_j) \in E \tag{94}$$

$$a_{s,i,j}^{B} \leq U * \bar{a}_{s,i,j}^{B} \qquad \forall s \in \{1, ..., m\}, \forall (v_i, v_j) \in E \tag{95}$$

$$a^A_{s,i,j} \geq \bar{a}^A_{s,i,j} \qquad \forall s \in \{1,...,m\}, \forall (v_i,v_j) \in E \tag{96}$$

$$a^B_{s,i,j} \geq \bar{a}^B_{s,i,j} \qquad \forall s \in \{1,...,m\}, \forall (v_i,v_j) \in E \tag{97}$$

$$\bar{a}^A_{s,i,j} + \bar{d}^A_{s,i,j} \leq 1 \qquad \forall s \in \{1,...,m\}, \forall (v_i,v_j) \in E \tag{98}$$

$$\bar{a}^B_{s,i,j} + \bar{d}^B_{s,i,j} \leq 1 \qquad \forall s \in \{1,...,m\}, \forall (v_i,v_j) \in E \tag{99}$$

$$\sum_{(v_i,v_j)\in E} \left(a^A_{s,i,j} + d^A_{s,i,j} + a^B_{s,i,j} + d^B_{s,i,j}\right) \leq \lambda_s \qquad \forall s \in \{1,...,m\}, \forall (v_i,v_j) \in E \tag{100}$$

$$c^A_{s,i} \geq \bar{a}^A_{s,i,j} \qquad \forall s \in \{1,...,m\}, \forall (v_i,v_j) \in E \tag{101}$$

$$c^B_{s,i} \geq \bar{a}^B_{s,i,j} \qquad \forall s \in \{1,...,m\}, \forall (v_i,v_j) \in E \tag{102}$$

$$a^A_{s,l} = \sum_{\substack{(v_i,v_j)\in E \\ v_i,v_j \in p_l}} a^A_{s,i,j} \qquad \forall s \in \{1,...,m\}, \forall p_l \in P \tag{103}$$

$$a^B_{s,l} = \sum_{\substack{(v_i,v_j)\in E \\ v_i,v_j \in p_l}} a^B_{s,i,j} \qquad \forall s \in \{1,...,m\}, \forall p_l \in P \tag{104}$$

$$d^A_{s,l} = \sum_{\substack{(v_i,v_j)\in E \\ v_i,v_j \in p_l}} d^A_{s,i,j} \qquad \forall s \in \{1,...,m\}, \forall p_l \in P \tag{105}$$

$$d^B_{s,l} = \sum_{\substack{(v_i,v_j)\in E \\ v_i,v_j \in p_l}} d^B_{s,i,j} \qquad \forall s \in \{1,...,m\}, \forall p_l \in P \tag{106}$$

$$a^A_{s,l} \geq 2 - H + H * \hat{a}^A_{s,l} \qquad \forall s \in \{1,...,m\}, \forall p_l \in P \tag{107}$$

$$a^B_{s,l} \geq 2 - H + H * \hat{a}^B_{s,l} \qquad \forall s \in \{1,...,m\}, \forall p_l \in P \tag{108}$$

$$a^A_{s,l} \leq 1 + H * \hat{a}^A_{s,l} \qquad \forall s \in \{1,...,m\}, \forall p_l \in P \tag{109}$$

$$a^B_{s,l} \leq 1 + H * \hat{a}^B_{s,l} \qquad \forall s \in \{1,...,m\}, \forall p_l \in P \tag{110}$$

$$d^A_{s,l} \geq 2 - H + H * \hat{a}^A_{s,l} \qquad \forall s \in \{1,...,m\}, \forall p_l \in P \tag{111}$$

$$d^B_{s,l} \geq 2 - H + H * \hat{a}^B_{s,l} \qquad \forall s \in \{1,...,m\}, \forall p_l \in P \tag{112}$$

$$d^A_{s,l} \leq 1 + H * \hat{a}^A_{s,l} \qquad \forall s \in \{1,...,m\}, \forall p_l \in P \tag{113}$$

$$d^B_{s,l} \leq 1 + H * \hat{a}^B_{s,l} \qquad \forall s \in \{1,...,m\}, \forall p_l \in P \tag{114}$$

$$\hat{a}^A_{s,l} + \hat{d}^A_{s,l} \leq 1 \qquad \forall s \in \{1,...,m\}, \forall p_l \in P \tag{115}$$

$$\hat{a}^B_{s,l} + \hat{d}^B_{s,l} \leq 1 \qquad \forall s \in \{1,...,m\}, \forall p_l \in P \tag{116}$$

$$\sum_{i=1}^{n} c^A_{s,i} u_{i,r} \geq f^{A,+}_{s,r} - M + M * z^{A,+}_{s,r} \qquad \forall s \in \{1,...,m\}, \forall r \in \{1,...,k\} \tag{117}$$

$$\sum_{i=1}^{n} c^A_{s,i} u_{i,r} \leq f^{A,+}_{s,r} + M * z^{A,+}_{s,r} \qquad \forall s \in \{1,...,m\}, \forall r \in \{1,...,k\} \tag{118}$$

$$\sum_{i=1}^{n} c^B_{s,i} u_{i,r} \geq f^{B,+}_{s,r} - M + M * z^{B,+}_{s,r} \qquad \forall s \in \{1,...,m\}, \forall r \in \{1,...,k\} \tag{119}$$

$$\sum_{i=1}^{n} c^B_{s,i} u_{i,r} \leq f^{B,+}_{s,r} + M * z^{B,+}_{s,r} \qquad \forall s \in \{1,...,m\}, \forall r \in \{1,...,k\} \tag{120}$$

$$\sum_{i=1}^{n} c^A_{s,i} u_{i,r} \leq f^{A,-}_{s,r} + M - M * z^{A,l}_{s,r} \qquad \forall s \in \{1,...,m\}, \forall r \in \{1,...,k\} \tag{121}$$

$$\sum_{i=1}^{n} c_{s,i}^{A} u_{i,r} \geq f_{s,r}^{A,-} - M * z_{s,r}^{A,-} \quad \forall s \in \{1, ..., m\}, \forall r \in \{1, ..., k\} \tag{122}$$

$$\sum_{i=1}^{n} c_{s,i}^{B} u_{i,r} \leq f_{s,r}^{B,-} + M - M * z_{s,r}^{B,l} \quad \forall s \in \{1, ..., m\}, \forall r \in \{1, ..., k\} \tag{123}$$

$$\sum_{i=1}^{n} c_{s,i}^{B} u_{i,r} \geq f_{s,r}^{B,-} - M * z_{s,r}^{B,-} \quad \forall s \in \{1, ..., m\}, \forall r \in \{1, ..., k\} \tag{124}$$

$$z_{s,r}^{A} \geq z_{s,r}^{A,-} \quad \forall s \in \{1, ..., m\}, \forall r \in \{1, ..., k\} \tag{125}$$

$$z_{s,r}^{B} \geq z_{s,r}^{A,-} \quad \forall s \in \{1, ..., m\}, \forall r \in \{1, ..., k\} \tag{126}$$

$$z_{s,r}^{A} \geq z_{s,r}^{A,+} \quad \forall s \in \{1, ..., m\}, \forall r \in \{1, ..., k\} \tag{127}$$

$$z_{s,r}^{B} \geq z_{s,r}^{A,+} \quad \forall s \in \{1, ..., m\}, \forall r \in \{1, ..., k\} \tag{128}$$

$$z_{s,r}^{A} \leq z_{s,r}^{A,+} + z_{s,r}^{A,-} \quad \forall s \in \{1, ..., m\}, \forall r \in \{1, ..., k\} \tag{129}$$

$$z_{s,r}^{B} \leq z_{s,r}^{A,+} + z_{s,r}^{A,-} \quad \forall s \in \{1, ..., m\}, \forall r \in \{1, ..., k\} \tag{130}$$

$$d_{s,r}^{A} \geq \sum_{i=1}^{n} c_{s,i}^{A} u_{i,r} - f_{s,r}^{A} \quad \forall s \in \{1, ..., m\}, \forall r \in \{1, ..., k\} \tag{131}$$

$$d_{s,r}^{B} \geq \sum_{i=1}^{n} c_{s,i}^{B} u_{i,r} - f_{s,r}^{B} \quad \forall s \in \{1, ..., m\}, \forall r \in \{1, ..., k\} \tag{132}$$

$$- d_{s,r}^{A} \leq \sum_{i=1}^{n} c_{s,i}^{A} u_{i,r} - f_{s,r}^{A} \quad \forall s \in \{1, ..., m\}, \forall r \in \{1, ..., k\} \tag{133}$$

$$- d_{s,r}^{B} \leq \sum_{i=1}^{n} c_{s,i}^{B} u_{i,r} - f_{s,r}^{B} \quad \forall s \in \{1, ..., m\}, \forall r \in \{1, ..., k\} \tag{134}$$

In the current implementation of ALPACA, we choose fixed parameters $U = M = H = 1000$. The parameter λ_s, which represents the maximum number of copy number amplifications and deletions permitted across all edges of the phylogenetic tree T for segment s, is varied across various thresholds as part of a model selection step. This procedure is described in the following section.

2.4 Model selection

For each segment $s \in \{1, ..., m\}$, we search for allele-specific clone copy numbers $c_{s,i}^{A}$ and $c_{s,i}^{A}$ that minimise the objectives subject to the constraints defined in section 2.3 above, for a certain value of $\lambda_s \in \mathbb{N}$. This value is varied in a model selection step, in which the following steps are undertaken.

To select the optimal solution for each genomic segment, we iterate ALPACA multiple times to generate solutions covering a range of allowed number of SCNA events, λ_s, and limit the maximum runtime of each iteration to 60 seconds. In each successive iteration λ_s is increased by 1.

To define the maximum number of iterations for each segment we apply the following heuristic: 1) by default the minimum number of iterations is 3 and maximum equals to either 20 or maximum rounded fractional copy number observed in samples, whichever is greater; 2) iterations are terminated early if optimisation time becomes longer than previously defined limit of 60 seconds and there is no improvement in the objective function in the last 3 iterations (defined as difference of at least 0.1 in objective function). These steps ensure that a wide range of solutions are produced while limiting consumption of computational resources.

3 Implementation

The ALPACA ILP was solved using the Gurobi solver (version 11.0.1) Gurobi Optimization, LLC 2023 and implemented in Python (version 3.9.13).

4 Computing the number of SCNAs on an edge

ALPACA infers copy-number events for each segment independently. However, it is known that SCNAs can overlap multiple genomic segments (Kaufmann et al. 2022; El-Kebir et al. 2017; Zeira et al. 2017; Zaccaria et al. 2018). Thus, we introduce a summary statistic, called *number of SCNAs on a tree edge N*, to count the actual number of copy-number events that occurred in the copy-number evolutionary tree inferred by ALPACA while taking into account neighbouring SCNAs. In contrast to previous studies, we acknowledge that this statistic does not necessarily compute the most parsimonious, or minimal number of interval events required to transform copy number profile $\mathbf{c}_i^A$ to $\mathbf{c}_j^A$, as other methods aim to derive, for example in Kauffman et al. (Kaufmann et al. 2022), El-Kebir et al. (El-Kebir et al. 2017) and Shamir et al. (Zeira et al. 2017). Also, similar to the other methods, we considered the SCNA evolution of alleles A and B separately.

We compute the number of copy number events occurring on each tree edge, or N, as follows for each edge $(v_i, v_j) \in E$ of the inferred phylogenetic tree T, and for each chromosome $P \in \{1, ..., 22\}$. Suppose chromosome P has p segments, and that ALPACA has inferred clone copy numbers $c_{s,i}^A, c_{s,i}^B, c_{s,j}^A, c_{s,j}^B$ for edge $(v_i, v_j) \in E$ and segment $s \in \{1, ..., p\}$. As such, for allele A (and equally for allele B), we define $\delta_{s,i,j} \in$ to be the copy number change for each segment on edge (v_i, v_j) such that:

$$\delta_{s,i,j} = c_{s,i} - c_{s,j} \qquad \forall s \in \{1, ..., p\} \tag{135}$$

Note that $\delta_{s,i,j}$ can be positive (reflecting an amplification) or negative (reflecting a deletion). Then, we defined a binary variable to indicate whether there was a difference between the copy number change that occurred on edge (v_i, v_j) on segment $s - 1$ and the following segment s. That is, we aimed to capture the first instances where $\delta_{s-1,i,j} \neq \delta_{s,i,j}$. Specifically, we defined $\bar{d}_{s,i,j}$ such that:

$$\bar{d}_{s,i,j} = \begin{cases} 1, & \text{if } s = 1 \\ \mathbb{1}_{\delta_{s-1,i,j} \neq \delta_{s,i,j}}, & \text{if } s \in \{2, ..., p\} \end{cases} \tag{136}$$

$$\tag{137}$$

Next, in order to extract the first segments of every contiguous stretch of the genome that was affected by a copy number event in the same direction, of the same amplitude, we computed the following multiplication:

$$\delta_{s,i,j} * \bar{d}_{s,i,j} \qquad \forall s \in \{1, ..., p\} \tag{138}$$

Hence, to quantify the number of copy number gains, $g_{i,j}$, that occurred on edge (v_i, v_j) within chromosome P, we computed:

$$g_{i,j} = \sum_{s=1}^{p} \mathbb{1}_{(\bar{d}_{s,i,j} * \delta_{s,i,j}) > 0} \tag{139}$$

Similarly, to quantify the number of copy number losses, $l_{i,j}$, that occurred on edge (v_i, v_j) within chromosome P, we computed:

$$l_{i,j} = \sum_{s=1}^{p} \mathbb{1}_{(\bar{d}_{s,i,j} * \delta_{s,i,j})<0} \tag{140}$$

To compute the total number of copy number events on chromosome P, for allele A, we computed the sum of $g_{i,j}$ and $l_{i,j}$:

$$e_{i,j}^{A,P} = g_{i,j} + l_{i,j} \tag{141}$$

Finally, to enumerate the total number of copy number events on edge (v_i, v_j), we took the sum across both alleles, across all chromosomes:

$$e_{i,j} = \sum_{P=1}^{22} e_{i,j}^{A,P} + e_{i,j}^{B,P} \tag{142}$$

5 Preparing input required by ALPACA

5.1 Preprocessing pipeline

To obtain input for ALPACA we recommend using Refphase (Watkins et al. 2023) and CONIPHER (Grigoriadis et al. 2024). See `https://github.com/McGranahanLab/ALPACA-pipeline` for an example pipeline for processing BAM files, running Refphase, CONIPHER and generating input required by ALPACA.

Example pipeline parameters

1. Germline Variants: Use only SNPs with coverage of at least 20 in all samples; use VAF threshold of 0.3 - 0.7 for heterozygous SNPs.
2. ASCAT: When running ASCAT for whole exome sequencing (WES) or whole genome sequencing (WGS) data, Gamma = 1 Van Loo 2010.
3. Refphase: Use default parameters.
4. Somatic variants: Use Genome Analysis Toolkit (GATK) best practices.
5. CONIPHER: We recommend applying the following two criteria to each SNV when transforming output from a variant calling tool (for example Mutect2 Benjamin et al. 2019) into CONIPHER input:
    - retain variants with total depth (across all samples) of at least 30
    - retain variants with at least 10 variant reads
    - reject variants with germline read count of 5 or more and with germline VAF of 1% or more
    - reject variants with tumour VAF below 5%

    Additionally, mutations should be 'force called' in all the samples, that is, if a variant is detected in one sample (and passes the thresholds described above), but is not detected in another sample, one should still count reads at the second sample at the variant locus. While running CONIPHER, use default parameters.
6. ALPACA: Use default parameters.

TRACERx pipeline parameters For detailed description of the processing pipeline used in TRACERx experiments see previous publications (Jamal-Hanjani et al. 2017; Frankell et al. 2023; Bakir et al. 2023)

5.2 Required inputs

Alternatively, if different tools are used in pre-processing, the user should create the following inputs for each tumour.

5.2.1 Fractional copy-numbers for each sample and each genomic segment

These should be stored in a data frame with the following columns:

segment	sample	cpnA	cpnB	tumour_id
1_6204266_6634901	U_LTXSIM001_SU_T1.R1	3.2	2.0	LTXSIM001
1_6204266_6634901	U_LTXSIM001_SU_T1.R2	3.3	2.3	LTXSIM001
1_6204266_6634901	U_LTXSIM001_SU_T1.R3	3.4	2.0	LTXSIM001

Table 1: First three rows of an example fractional copy number table input to ALPACA.

The table above (Table 1) shows the input for one genomic segment located on chromosome 1, starting at the base 6204266 and ending at 6634901 (encoded in the segment name as `<chr>_<start>_<end>`). Column 'sample' contains sample names of the tumour: this example contains 3 different samples (R1, R2 and R3) obtained from a single tumour (`U_LTXSIM001_SU_T1`). The sample names are arbitrary, but must be coherent within the entire input (including other input files). Allele-specific fractional copy-numbers are stored in columns `cpnA` and `cpnB`. Column `tumour_id` stores the identifier of the tumour.

The segments are stored in the `ALPACA_input_table.csv` file.

IMPORTANT

Pay special attention to underscore _ character - it is used by ALPACA during file parsing and its usage must conform to the example pattern shown above. *Do not use it in your tumour identifier.*

5.2.2 Confidence intervals associated with each allele-specific fractional copy-number

This table (called `ci_table.csv` is similar to the ALPACA_input_table but contains lower and upper confidence intervals for each genomic segment (Table 2)).

segment	sample	lower_CI_A	upper_CI_A	lower_CI_B	upper_CI_B	tumour_id	ci_value
10_38599060_42906137	LTXSIM001_SU_T1-R1	3.218	4.196	2.200	3.085	LTXSIM001	0.5
10_38599060_42906137	LTXSIM001_SU_T1-R2	1.468	1.695	2.703	2.977	LTXSIM001	0.5

Table 2: First two rows of an example table of segment-level copy number confidence intervals for input to ALPACA.

5.2.3 Clone proportions table

This is a table containing the cellular prevalence of each clone in each sample, saved as a comma separated file under the name `cp_table.csv` (Table 3). Clone proportion values can be derived from cancer cell fractions (CCF), for example by subtracting the CCF values of children clones from CCF values of their parents. For

example, the `compute_subclone_proportions` function from the CONIPHER R package (`https://github.com/McGranahanLab/CONIPHER`) can be used to infer clone proportions from CCF values.

The format of the clone proportions table is as follows. The table contains an index column specifying clone names (which must match the name of clones in phylogenetic tree - see below) and one column for each sample. Proportions should sum to 1 in each sample, but small deviations from 1 are tolerated.

clone	U_LTXSIM001_SU_T1.R1	U_LTXSIM001_SU_T1.R2	U_LTXSIM001_SU_T1.R3
clone1	0.0309	0.0006	0.1383
clone12	0.2810	0.0	0.0
clone13	0.0	0.0253	0.1112
clone14	0.1557	0.0	0.0021
clone15	0.0	0.0	0.1598
clone19	0.0	0.4785	0.2534
clone20	0.0202	0.4460	0.3176
clone21	0.0684	0.0	0.0174
clone8	0.4434	0.0495	0.0
clone16	0.0	0.0	0.0
clone18	0.0	0.0	0.0

Table 3: Example clone proportion table for input to ALPACA.

5.2.4 Phylogenetic tree

A json file (named `tree_paths.json`) containing the SNV tree structured encoded as an array of arrays - each of the sub-arrays represents the phylogenetic path from the trunk (most recent common ancestor) to a terminal clone (leaf). For example, consider a simple tree with a MRCA clone and three subclones. Subclones A and B are direct descendants of MRCA, and clone C is the child of clone B:

```
MRCA
├── A
└── B
    └── C
```

Such a tree would be encoded as following in ALPACA format: [['MRCA','A'],['MRCA','B','C']] A more complex tree, with name of clones consistent with names used in the `cp_table.csv` above would look like this:

```
[["clone12","clone13","clone14","clone8"],["clone12","clone13","clone14",
"clone15"],["clone12","clone13","clone16","clone18","clone1"],["clone12",
"clone19","clone20"],["clone12","clone19","clone21"]]
```

5.2.5 Example input file structure

Overall, for each tumour we expect the following files:

```
LTXSIM001
├── ALPACA_input_table.csv
├── ci_table.csv
├── cp_table.csv
└── tree_paths.json
```

5.2.6 Running ALPACA

See `https://github.com/McGranahanLab/ALPACA-model` for detailed instructions describing the installation and running of ALPACA.

6 Validation of the model selection procedure

In an idealised scenario, where the true values of the fractional copy-numbers and clone proportions were known, the optimal model would be characterised by the objective function value of zero. In practice, however, observed fractional values are point estimates of the underlying values and are influenced by stochastic variations. Therefore, the objective function value is expected to decrease with increasing complexity, but never reach an exact value of zero. The small improvements in the objective function at high complexities are likely representing over-fitting rather than reflecting the true copy-number events. To select the best solution while balancing between increased model complexity and better fit to the data, we thus employed a model selection approach based on the previously proposed Kneedle algorithm (Satopaa et al. 2011 - implemented in Python package kneed version 0.8.5 with the following parameters: sensitivity=1-200, interp_method="interp1d", online=True), similarly to previous combinatorial models (Zaccaria and Raphael 2020; Myers et al. 2023).

To determine whether the most selected solution is optimal, we reran both the CONIPHER and ALPACA on a subcohort of simulated tumours. We created this subcohort by sorting all the tumours by the complexity of their phylogenetic trees, and choosing every 15th tumour, which yielded a representative subset of 11 tumours. For these simulated tumours, we retained the computed potential solutions for all the complexity values, alongside the solution selected by ALPACA. Next, for each tumour, each segment and each solution, we computed the total variation distance (TVD, see Methods section in the main manuscript) between the chosen ALPACA solution and the ground truth copy-number state. TVD value of 0 represents solutions where copy-number states and clone-proportions associated with them match exactly the values found in the ground truth. Conversely, TVD values of 1 represent solutions without any such match. We then focused on the following 4 groups of solutions:

1. "Diploid": in this case, a diploid solution is chosen regardless of model fit.
2. "ALPACA": solutions selected with our model selection procedure,
3. "ALPACA-1": solutions where complexity equals the complexity of ALPACA solutions minus one,
4. "ALPACA+1": solutions where complexity equals the complexity of ALPACA solutions plus one,
5. and "Minimal TVD" solutions, i.e. solutions with the lowest TVD, representing solutions closest to the ground truth. Note that solutions in this group might overlap with solutions in other groups, i.e. a solution with the minimal TVD might also be the one selected by ALPACA. This is the best potential solution that could be chosen.

The mean TVD value for the diploid solutions in this subcohort was 0.65, the value for ALPACA solutions was 0.10, while the value for the minimal TVD was 0.05. Both ALPACA-1 and ALPACA+1 solutions had higher TVD compared to ALPACA solutions (0.35 and 0.12, respectively) (Figure 1).

Next, we categorised segments into three groups: (1) segments where the TVD of the ALPACA solution was minimal, (2) segments where another, more complex solution with lower TVD existed, 3) segments where

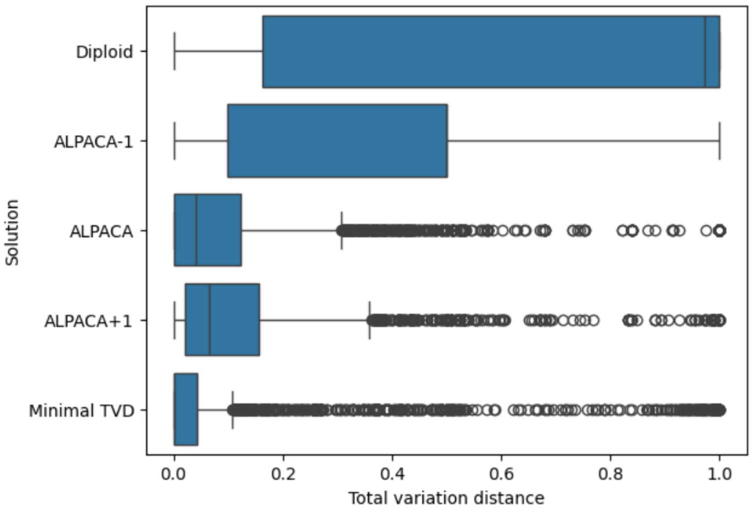

Figure 1: Total variation distance per genomic segment in the subcohort of 11 simulated tumours, split by solution type.

another, less complex solution with lower TVD existed. In other words, these three categories correspond to solutions where our elbow point was correct, too simple or too complex. Using this classification, we plotted a histogram of segment frequency against complexity error (Figure 2). Reassuringly, the results showed that in 51% of segments, no alternative solution had a lower TVD than the ALPACA solution. Moreover, when the elbow solution was suboptimal, the error was relatively small: in 82% of segments, the absolute complexity error was 4 or less (Figure 3). It is also worth noting that the complexity error was clearly centred at 0 (Figure 2), suggesting a reasonable balance between picking an elbow point that is too simple or too complex.

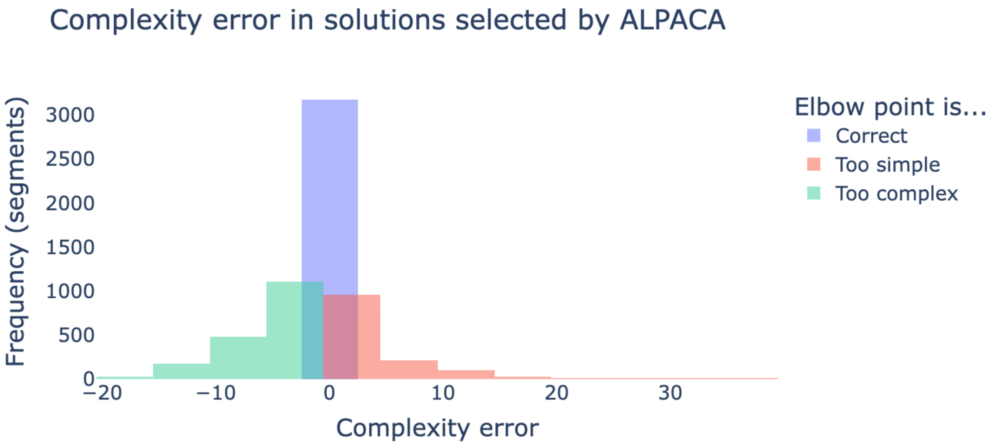

Figure 2: Frequency of complexity error of the elbow solution. "Complexity error" represents the difference in complexity between the solution found with elbow search and the solution most similar to the ground truth (i.e. minimal TVD), from the pool of all the solutions found by ALPACA in the analysed subcohort (n=11).

To provide an estimate of how the results of our analysis would change if ALPACA always selected the solution with minimal TVD, we calculated the difference between the copy-number profiles of the solutions selected by ALPACA and the minimal TVD solutions and obtained the mean value of 0.12 per genomic

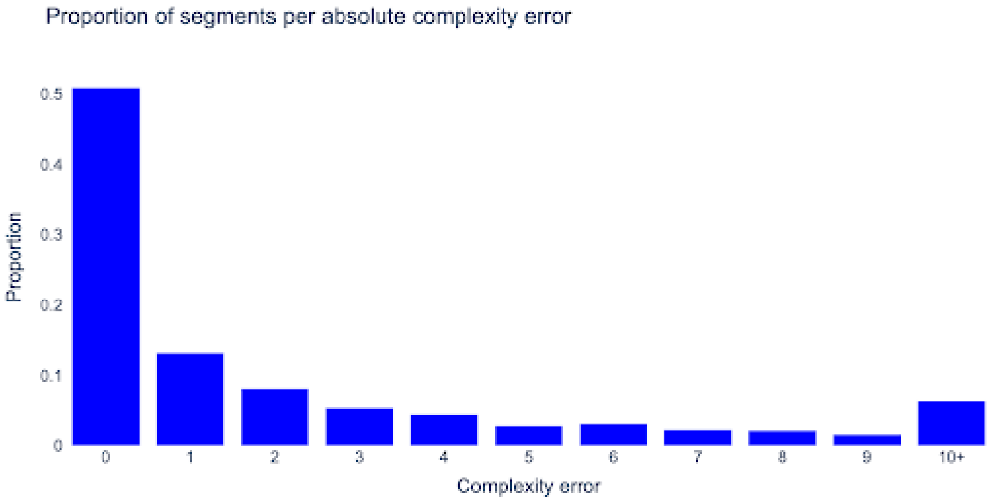

Figure 3: Proportion of segments per absolute complexity error in the analysed subcohort (n=11).

segment. The results of this comparison are presented in the histogram in Figure 4.

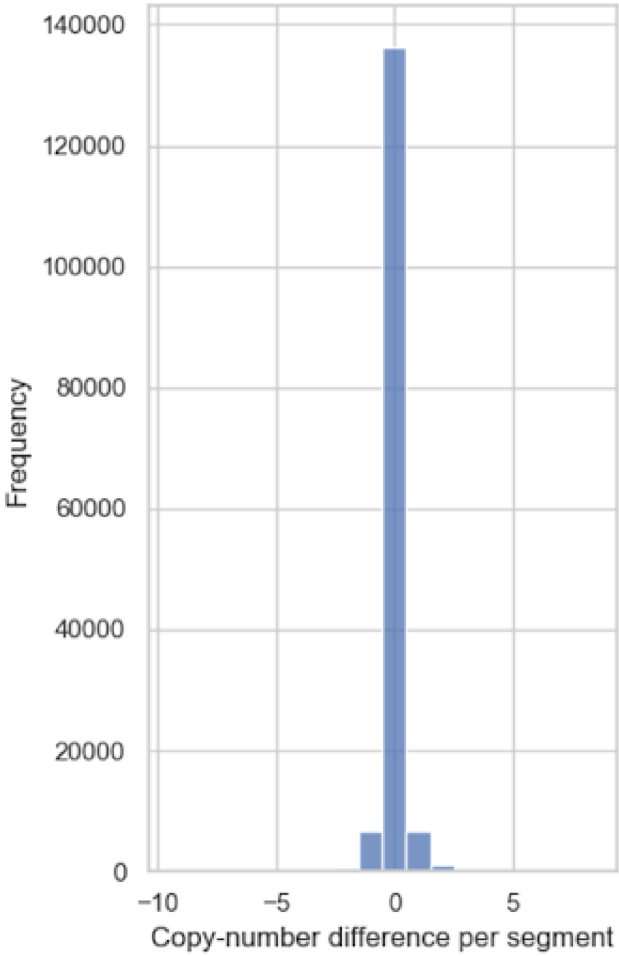

Figure 4: Histogram of the frequency of copy-number differences per segment between the solutions selected by ALPACA and minimal TVD solutions in the analysed subcohort (n=11).

Taken together, these data suggest that ALPACA does not always pick the perfect solution. However, in the context of the simulations, the model selection procedure achieves a reasonable balance between too complex and too simple solutions. Furthermore, on average, the difference between TVD of solutions selected by ALPACA and diploid solutions is much larger compared to the difference between ALPACA solutions and minimal TVD solutions (0.55 vs 0.05). Additionally, the mean per-segment copy-number differences between ALPACA solutions and minimal TVD solutions were low, suggesting that the analysis presented in the manuscript is not strongly affected by errors in the model selection procedure.

7 Validation of ALPACA assumptions

7.1 Estimating sufficient SNV acquisition rate with simulations

In this section, we describe a validation study we performed to investigate the underlying assumption in ALPACA that tumour clonal structure can be inferred solely from SNVs and then subsequently used to infer copy number evolution.

7.1.1 Background and aims

Algorithms which aim to reconstruct tumour phylogenies from sequencing data first require the identification of related *clones*, groups of cells which have particular mutations in common. Parent-child relationships between these clones are then inferred from their frequencies and genetic relatedness, and the clones are then placed within a lineage tree. When the input data consists of bulk sequencing of samples from discrete tumour regions, clones are identified by grouping together mutations at similar frequencies. When single-cell data is used as input, individual cells are grouped together using their particular mutations.

Many such clustering algorithms use information from single-nucleotide variants (SNVs), and ignore ancestral information which may be encoded in copy-number alterations (CNAs). During cancer development, cells acquire both SNVs and CNAs on division, and so both types of mutations encode 'barcoding' information about the lineage of a cell. As a result, important information about clonal structure may be gained when CNAs are included. We may discern finer clonal distinctions (such that cells are reassigned to different clones), or discover that two apparently unrelated cells have a CNA in common (such that cells are reassigned to the same clone). The amount of information provided by CNAs will depend on the relative rates with which SNVs and CNAs are acquired. If SNVs are much more common than CNAs, then all relevant details about cell lineage will be captured by SNVs, and CNAs may be safely ignored. If CNAs are relatively common, however, their exclusion may have a significant impact the clonal structure.

Here we use simulations of cell division, mutation and death to determine the threshold CNA and SNV mutation rates at which clonal structure may be safely inferred from SNVs alone. We find that, regardless of the rate of acquisition of CNAs, SNVs alone are sufficient to assign cells to clones when they are acquired in the exome at a rate of roughly 0.3 per cell division. The mutation rate of the exome in non-small cell lung cancer (NSCLC) has been estimated at between 0.42 and 1.56 per cell division (Werner et al. 2020), so we conclude that in this context CNAs can be safely excluded when estimating clonal structure.

7.1.2 Methods

Modelling tumour-cell division and death We use an agent-based simulation framework in which cells divide, mutate and die, with variable mitotic age and at a fixed population size (roughly $N_0 = 1000$

cells, about the maximum size of a single-cell sequencing sample from an individual tumour region). We use timesteps of one day. The population begins from a single cell with no mutations. The founding cell divides with probability $p_0 = 0.631$ per day, based on a cell-cycle time of 24 hours and a Poisson-distributed division time. (This is lengthened slightly from experimental estimates of 22 hours taken from NSCLC cell lines (Matthews et al. 1989; Eden et al. 2011), on the assumption that cells are less well-adapted and thus slower to divide in the early stages of tumour evolution.) At each division, cells can acquire either CNAs or SNVs. As it is computationally unfeasible to represent all $M_0 = 30$ million base pairs in the exome, we assume there are $M_1 = 1000$ 'effective loci' at which SNVs can occur, and another $M_1 = 1000$ possible CNA mutations, represented by vectors of length M_1 in which all entries are initially 0. Each CNA 'locus' represents a region of the genome, rather than an individual base pair. By setting $M_0 = M_1 = 1000$, we are modelling a system in which there are 1000 *possible* CNAs or SNVs that can occur in a cell (and many more possible combinations thereof). For simplicity, we assume that CNAs and SNVs do not interact, and are fundamentally different types of mutation.

We examine mutation rates $\mu_{\text{CNA}}, \mu_{\text{SNV}} \in [0.0, 0.8]$, measured in mutations per cell division. We scale the mutation rate of each 'effective' base pair to accommodate its condensed size, so that at each timestep, every un-mutated base pair has a $\mu' = \frac{\mu}{M_1}$ of flipping from 0 to 1. At the upper limit, $\mu = 0.8$, we expect around a fifth of all sites in a cell to be mutated by the end of the simulation. (Each cell has roughly a $p_0 = 0.631$ chance of dividing at each timestep, and so each locus has a $\mu' p_0$ chance of mutating; the probability that it will have mutated after 365 days is $1 - (1 - \mu' p_0)^{365} \approx 0.17$. This result is fairly robust to the effect of selection; if the division rate p is 20% higher than p_0, we expect 0.2 sites to have mutated after a year.)

We simulate scenarios where selective advantages are conferred by particular CNA sites, particular SNV and CNA sites, or neither. Where selection is present, each site is allocated 'driver' status with probability 1 in 1,000 (about two orders of magnitude higher than literature estimates (Bozic et al. 2010), to ensure that there is likely to be at least one driver site in the condensed exome). If a cell has k mutated driver sites, and assuming a selection strength s, then its division probability in a timestep is

$$p_p(s, k, p_0) = p_0 + (1 - p_0)(1 - (1 - s)^k)$$

This is equal to p_0 when $s = 0$ or $k = 0$, and approaches 1 asymptotically as $s \to 1$ or $k \to \infty$, to avoid nonsensical division probabilities ($p_p > 1$). We assume a selection strength of $s = 0.1$, which represents 'strong selection' by literature convention.

Once cells have divided and mutated, we implement cell death, to keeo population size roughly constant. In the absence of selection, this is achieved by enforcing a random death rate of $\delta_0 = \frac{p_0}{1+p_0}$ per timestep where $N > N_0$ (calculated such that the expected population size after division and death is $N(1+p_0)(1-\delta_0) = N$). In the presence of selection, cells directly compete for survival based on fitness when $N > N_0$. A cell's survival-fitness f_s is multiplicatively dependent on its k driver mutations, $f_s = (1 + s)^k$, and its probability of survival is $p_s = N_0 \frac{f_s}{F}$, where F is the sum of the fitness of all cells in the system. (We note that when all cells have the same fitness, and the system begins a timestep at capacity $N = N_0$, this is equivalent to the neutral-growth scheme: we expect a population size $N = N_0(1 + p_0)$, so this results in an average survival probability of $p_s = \frac{1}{1+p_0}$ and so a death rate $1 - p_s = \frac{p_0}{1+p_0} = \delta_0$).

Measuring the impact of CNAs on observed clonal structure After 1 simulated year, simulated cells are sampled and grouped together into clones. We are interested in measuring the consistency in clonal assignments when clustering is performed with and without incorporating CNAs.

We assume perfect sequencing, such that we are able to detect with 100% certainty whether a particular locus is mutated within a cell. The ith cell is represented by the set S_i of indices which represent mutated loci. When only SNVs are used to perform clustering, integers in S_i can take M_1 possible values between 0 and $M_1 - 1$. When CNAs are included, the possible sites of SNVs and CNAs are treated as a single 'condensed exome' with effective size $2M_1$, and so integers in S_i can take $2M_1$ possible values between 0 and $2M_1 - 1$.

We group cells together using hierarchical clustering with complete linkage and a precomputed affinity matrix, using the clustering package in scikit-learn (Pedregosa et al. 2011). The (i, j)th entry in the affinity matrix, a_{ij} describes the genetic similarity between cells i and j, defined as

$$a_{ij} = \frac{|S_i \cap S_j|}{|S_i \cup S_j|}$$

i.e. the fraction of all mutations appearing in either cell which appear in both cells. a_{ij} is 1 where two cells are genetically identical (and is set to 1 by default where $|S_i \cup S_j| = 0$, i.e. where no mutations are detected in either cell), and 0 where two cells have no mutations in common.

We set out to identify the optimal number of clusters, K, which describes the population. We assume the population contains at least 2 and at most 10 clones. For each $K \in [2, 10]$, we cluster cells into K clones, and measure the optimality of this K using silhouette scoring (Rousseeuw 1987). The average silhouette score of a clustering scheme measures how much closer a cell is to its assigned cluster than to other clusters, and takes value $[-1, 1]$, with a higher value indicating a better fit. We use silhouette scoring as implemented by the scikit-learn metrics package, which requires a distance matrix; for this we define the distance between two cells as the complement of the affinity, $d_{ij} = 1 - a_{ij}$, such that $d_{ij} = 1$ for cells with no mutations in common and 0 for identical cells.

This scheme results in an optimal K and a corresponding set of cell labels, L_i, which take integer values. Cells with the same labels are assigned to the same clone. We run this procedure twice, once combining SNV and CNA mutations and once with SNVs alone, to obtain two sets of optimal labels. We then seek to measure the consistency between two clustering schemes, defined as the fraction of all possible cell pairs which are consistently clustered. A pair of cells is clustered consistently if they are assigned to the same cluster in both schemes, or to different clusters in both schemes. Clustering consistency C takes values between 0 and 1.

7.1.3 Results

We explore values of CNA and SNV mutation rates between 0 and 0.8 exome mutations per division ($\mu_{\text{CNA}}, \mu_{\text{SNV}} \in [0.0, 0.8]$). For neutral growth (see Figure 5), we find that a consistency of roughly 80% is achieved for $\mu_{\text{SNV}} \geq 0.3$, regardless of μ_{CNA}, and thus we can conclude that within the range of mutation rates characteristic to NSCLC ($\mu_{\text{SNV}} \geq 0.4$), SNVs are sufficient to barcode relevant lineages. Total consistency (greater than 90%) is only achieved when no CNVs are present. We notice a 'valley' of inconsistency when SNV and CNA mutation rates are low but nonzero (approximately$\mu_{\text{CNA}}, \mu_{\text{SNV}} < 0.15$), when mutations are rare enough that the addition of CNAs completely reconfigures the clustering scheme. Consistency is lowest (< 0.5) when $\mu_{\text{SNV}} < 0.15$ and $\mu_{\text{CNA}} > 0.15$, when most clonal structure is encoded in CNAs and not SNVs.

When drivers are present, either as CNAs or within both mutation types, this region of inconsistency becomes narrower, and an SNV mutation rate $\mu_{\text{SNV}} \geq 0.2$ is sufficient to capture clonal structure. This decreased threshold can be explained by the fact that selection pressures tend to decrease the diversity in a

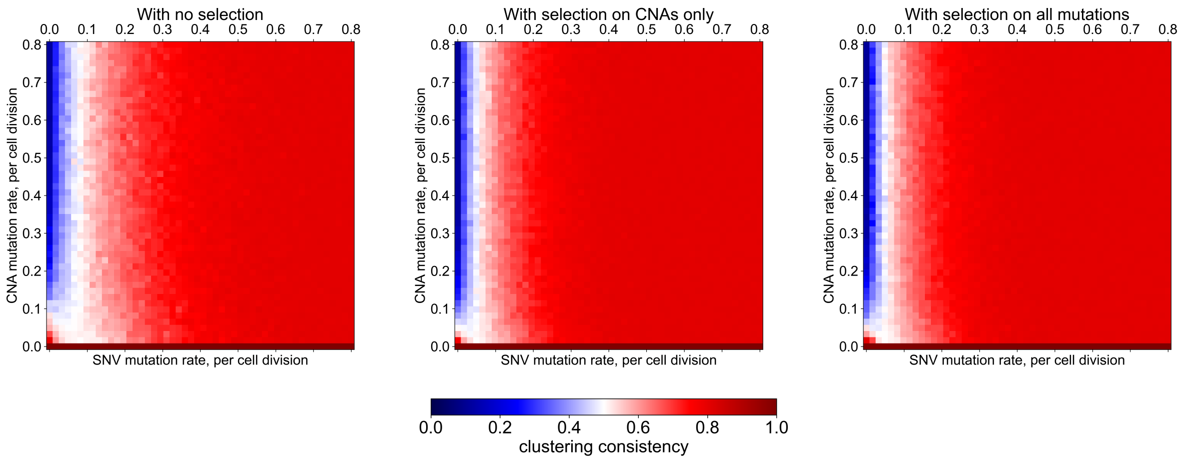

Figure 5: Variation of clustering consistency with SNV and CNA mutation rates, assuming neutral growth (left), selection on CNAs only (middle), and selection on both CNAs and SNVs (right). For all pairs of mutation rates, consistency values are averaged across 10 simulations.

population, leading to deeper, broader trees and fewer surviving lineages. This increases the informational redundancy in all mutations, as cells are in general are more closely related. CNAs are less helpful in identifying a cell's place on a phylogenetic tree, and so a lower SNV rate is necessary to identify relevant clones.

Given these results and published estimations of mutations rates (Werner et al. 2020) we postulate that phylogeny reconstructed with SNVs is suitable for subsequent copy-number inference in cancer types such as non-small cell lung cancer, renal cell carcinoma and colorectal cancer both with and without microsatellite instability (see Table 4).

Tumour mutation burden (TMB: defined in this context as the number of somatic mutations per megabase) can be considered a proxy of mutation rate. Therefore, we postulate that our method could be applied to to cancer types exhibiting TMB similar or above the value estimated for kidney cancer, for example ovarian cancer or melanoma (Sha et al. 2020; Alexandrov et al. 2013).

Cancer type	Low estimate	High estimate	Pass 0.3 threshold
NSCLC	0.42	1.59	yes
Renal cell carcinoma	0.063	0.936	partially
Colorectal MSI	28.48	98.88	yes
Colorectal MSS	0.375	0.903	yes

Table 4: Exome mutations per cell division in non-small cell lung cancer (NSCLC), renal cell carcinoma and colorectal cancers (MSI - with microsatellite instability, MSS - without microsatellite instability).

7.2 Evaluating influence of SCNA affected genomic segments on SNV-based phylogeny reconstruction

ALPACA leverages SNV-derived phylogeny as a 'scaffold' to assign copy-numbers to each clone identified by clustering mutations from across the genome. However, these mutations are sometimes found on genomic segments affected by copy-number events. Tools such as CONIPHER (Grigoriadis et al. 2024) perform a

copy-number correction to account for the overlap between SNV mutations and SCNA events to minimize the effect such overlap could have on the estimate of cancer cell fractions and in consequence the structure of the phylogenetic tree. When applying the CONIPHER algorithm to reconstruct the phylogenetic trees from all tumours in the TRACERx421 dataset, the authors investigated the mutations that were removed by CONIPHER that were driven by SCNAs (see Supplementary Figure 1 in Grigoriadis et al. 2024). These "CN removed" mutations had similar mutational signature distribution to truncal mutations, indicating they were affected by missed subclonal SCNA and were truncal. These would result in incorrect CCF so were rightly removed.

Nevertheless, to further evaluate whether copy number alterations could result in errors in the inferred phylogenetic tree, we also evaluated whether removal of mutations in regions subject to extensive copy number alteration altered the inferred phylogeny. If copy number alterations lead to erroneous CCFs, this should lead to corresponding erroneous clusters, and thereby an incorrect phylogenetic tree. Reassuringly, however, we observed highly consistent inferred phylogenies and clusters when these mutations were removed.

To conduct this analysis, we first calculated the coefficient of variation for each allele's fractional copy number across all samples within a genomic segment. We then averaged these values to obtain the mean coefficient of variation for each segment. Next, we ranked the genomic segments within each tumour based on their mean coefficient of variation and created four cohorts by progressively filtering out the top 5%, 15%, 25%, or 50% of the genome with the highest variation. Next, we ran CONIPHER on each of these cohorts and compared the results to the output created without any filtering, i.e. containing all the genomic segments, including the ones affected by SCNA. To evaluate the results, we calculated clustering concordance scores for each pair of mutations, by checking if mutations in both sets of results retain the same phylogenetic relationships. We classified each pair of mutations as either: a) belonging to the same cluster, b) ancestor-descendent, c) descendant-ancestor or d) parallel (not belonging to any other class). We scored each pair as concordant, if the class of phylogenetic relationship was found to be the same in the full cohort and filtered cohort. The final score represents the fraction of concordant pairs out of all the possible pairs (Figure 6).

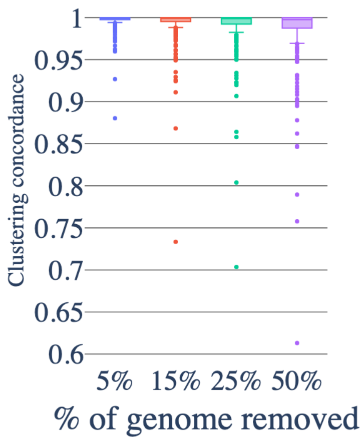

Figure 6: Clustering concordance for 310 tumours from TRACERx primary cohort. Each box plot represents one subcohort, where 5%, 15%, 25%, or 50% of the genome with extensive SCNA has been removed

Additionally, we present an example of phylogenetic trees obtained with CONIPHER for a single case CRUK0416 comparing the results using unaltered input with four test cohorts describe above. As can be

seen in Figure 7, although there are slight fluctuations in the tree (as would be expected), the structure is highly concordant, even when over 50% of the genome is ignored. Together, these results strongly support the notion that CONIPHER is robust to heterogeneous copy number alterations and that the SNV-based phylogenies required as input to ALPACA are sufficiently accurate when using CONIPHER.

7.3 Evaluating effects of using alternative CONIPHER trees

CONIPHER can generate multiple plausible phylogenetic trees, referred to as "alternative trees," each accompanied by a likelihood metric. In this manuscript, we always used the optimal tree output by CONIPHER, referred to as the "lowest error tree". However, to explore the impact of using alternative trees, we followed this procedure: From the simulated cohort described in the manuscript (TRACERx simulations), we excluded cases with fewer than 10 alternative trees. We then sorted the remaining tumours based on the number of alternative trees and selected 10 tumours evenly spaced across this range, from the simplest case (12 alternative trees) to the most complex (47,608 alternative trees). For each of these tumours, we further selected 9 alternative trees using the following method: we sorted the alternative trees by their likelihood metric (provided by CONIPHER) and selected them at regular intervals. Each selected tree was assigned a rank from 1 to 9, where rank 1 corresponded to the first alternative tree and rank 9 to the last alternative tree with the highest CONIPHER error. The "lowest error tree" was assigned rank 0. We then evaluated each rank by comparing its results to the known ground truth, using the same scoring method described previously (mean Hamming distance to the most similar copy-number profile; see "Methods" section of the main manuscript). Our results show that the outcomes remain consistent across all tested alternative trees, as illustrated in Figure 8.

Thus, this suggests that the output of ALPACA is not strongly influenced by which alternative tree is used, provided the trees are considered sufficiently accurate by CONIPHER.

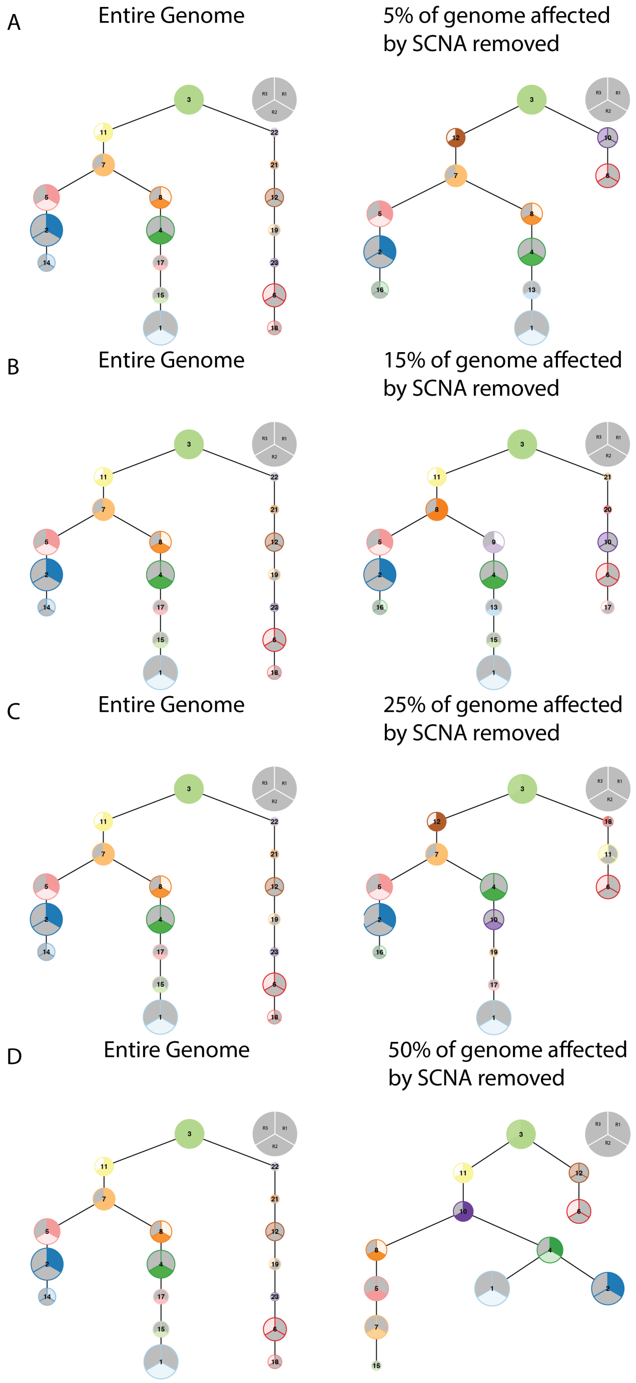

Figure 7: Four panels (A, B, C, D) comparing the phylogenetic tree of a 3-sample TRACERx case (CRUK0416) obtained with CONIPHER using the full available input (left side of each panel) with a tree based on a truncated input. In each of the panels, 5% (A), 15% (B), 25% (C) or 50% (D) of the genome characterised by most pronounced SCNA events has been removed. Each node represents a single SNV clone and is divided into three wedges, each representing a single sample. Colouring of each wedge represents presence or absence of clone in a sample.

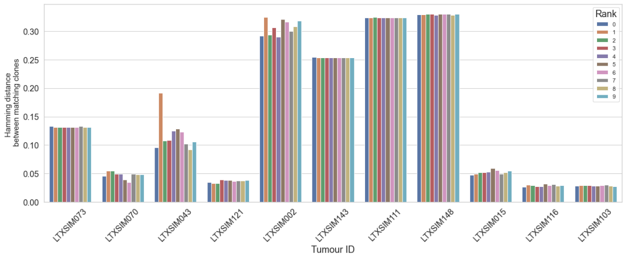

Figure 8: Mean Hamming distance between matching clones in the ground truth dataset and model predictions for a subset of simulated tumours. Scores are shown for trees of each rank from 0 to 9, with rank 0 representing the "lowest error tree."

7.4 Evaluating effects of uncertainty in clone proportions derived from SNVs

ALPACA takes clone proportion values as fixed inputs, which can be inferred from cancer cell fractions (CCFs) after phylogenetic reconstruction of SNVs using standard methodology (as described above in Section 1).

To evaluate the effects of upstream errors in CCF estimation on ALPACA results, we ran ALPACA on a simulated dataset that was generated with downsampled effective sequencing coverage (n=10) (Grigoriadis et al. 2024). We reconstructed the clone proportions and tumour phylogeny using CONIPHER based on SNVs, then ran ALPACA on these inputs, plus the ground truth fractional copy number in each sample. We observed that as coverage increased, the total variation distance between the simulated and predicted copy number states and clone sizes decreased (Figure 9).

Thus, this suggests that increased coverage improves the accuracy of the trees and phylogenetic reconstruction (using SNVs) and, accordingly, this improves the accuracy of ALPACA.

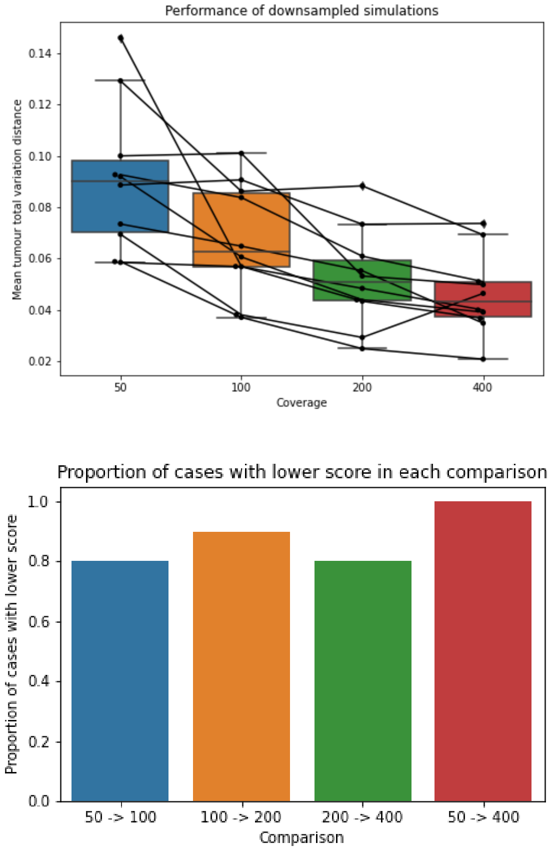

Figure 9: A) The total variation distance (mean per tumour) between the inferred clone copy number states and ground truth copy number states for a set of simulations with downsampled coverage (n=10 simulations each with coverage values of 50x, 100x, 200x, 400x). B) The proportion of cases with a lower total variation distance score when comparing the datasets with downsampled coverage 50x vs 100x, 100x vs 200x, 200x vs 400x, and 50x vs 400x.

References

Alexandrov, L. B. et al. (Aug. 2013). "Signatures of mutational processes in human cancer". en. In: *Nature* 500.7463, pp. 415–421.

Bakir, M. A. et al. (Dec. 2023). "The evolution of non-small cell lung cancer metastases in TRACERx". In: *Nature* 616 (7957), pp. 534–542. ISSN: 14764687. DOI: 10.1038/s41586-023-05729-x.

Benjamin, D., T. Sato, K. Cibulskis, G. Getz, C. Stewart, and L. Lichtenstein (2019). "Calling Somatic SNVs and Indels with Mutect2". In: *bioRxiv*. DOI: 10.1101/861054. URL: https://www.biorxiv.org/content/early/2019/12/02/861054.

Bozic, I., T. Antal, H. Ohtsuki, H. Carter, D. Kim, S. Chen, R. Karchin, K. W. Kinzler, B. Vogelstein, and M. A. Nowak (Dec. 2010). "Accumulation of driver and passenger mutations during tumor progression". In: *Proceedings of the National Academy of Sciences* 107 (43), pp. 18545–18550. ISSN: 0027-8424. DOI: 10.1073/pnas.1010978107.

Carter, S. (2012). "Absolute quantification of somatic DNA alterations in human cancer". In: *Nature Biotechnology* 30.5, pp. 413–321. DOI: https://doi.org/10.1038/nbt.2203.

Eden, E., N. Geva-Zatorsky, I. Issaeva, A. Cohen, E. Dekel, T. Danon, L. Cohen, A. Mayo, and U. Alon (Feb. 2011). "Proteome Half-Life Dynamics in Living Human Cells". In: *Science* 331 (6018), pp. 764–768. ISSN: 0036-8075. DOI: 10.1126/science.1199784.

Favero, F. (2015). "Sequenza: allele-specific copy number and mutation profiles from tumor sequencing data". In: *Annals of Oncology* 26 (1), pp. 64–70. DOI: https://doi.org/10.1093/annonc/mdu479.

Frankell, A. M. et al. (Apr. 2023). "The evolution of lung cancer and impact of subclonal selection in TRACERx". In: *Nature* 616 (7957), pp. 525–533. ISSN: 0028-0836. DOI: 10.1038/s41586-023-05783-5.

Grigoriadis, K. et al. (Jan. 2024). "CONIPHER: a computational framework for scalable phylogenetic reconstruction with error correction". en. In: *Nature Protocols* 19.1, pp. 159–183.

Gurobi Optimization, LLC (2023). *Gurobi Optimizer Reference Manual.* URL: https://www.gurobi.com.

Ha, G. (2014). "TITAN: inference of copy number architectures in clonal cell populations from tumor whole-genome sequence data". In: *Genome Research* 24 (11), pp. 1881–1892. DOI: https://doi.org/10.1101/gr.180281.114.

— (2018). "Copy-number analysis and inference of subclonal populations in cancer genomes using Sclust". In: *Nature Protocols* 13.6, pp. 1488–1501. DOI: https://doi.org/10.1038/nprot.2018.033.

Jamal-Hanjani, M. et al. (2017). "Tracking the Evolution of Non–Small-Cell Lung Cancer". In: *New England Journal of Medicine* 376.22, pp. 2109–2121. DOI: 10.1056/NEJMoa1616288. eprint: https://www.nejm.org/doi/pdf/10.1056/NEJMoa1616288. URL: https://www.nejm.org/doi/full/10.1056/NEJMoa1616288.

Jiang, Y., Y. Qiu, A. J. Minn, and N. R. Zhang (Sept. 2016). "Assessing intratumor heterogeneity and tracking longitudinal and spatial clonal evolutionary history by next-generation sequencing". en. In: *Proc. Natl. Acad. Sci. U. S. A.* 113.37, E5528–37.

Kaufmann, T. L. et al. (Nov. 2022). "MEDICC2: whole-genome doubling aware copy-number phylogenies for cancer evolution". en. In: *Genome Biol.* 23.1, p. 241.

El-Kebir, M., B. J. Raphael, R. Shamir, R. Sharan, S. Zaccaria, M. Zehavi, and R. Zeira (May 2017). "Complexity and algorithms for copy-number evolution problems". en. In: *Algorithms Mol. Biol.* 12, p. 13.

El-Kebir, M., G. Satas, L. Oesper, and B. J. Raphael (July 2016). "Inferring the Mutational History of a Tumor Using Multi-state Perfect Phylogeny Mixtures". en. In: *Cell Syst* 3.1, pp. 43–53.

Malikic, S., A. W. McPherson, N. Donmez, and C. S. Sahinalp (May 2015). "Clonality inference in multiple tumor samples using phylogeny". en. In: *Bioinformatics* 31.9, pp. 1349–1356.

Matthews, J., B. Meeker, and J. Chapman (Jan. 1989). "Response of human tumor cell lines in vitro to fractionated irradiation". In: *International Journal of Radiation Oncology*Biology*Physics* 16 (1), pp. 133–138. ISSN: 03603016. DOI: 10.1016/0360-3016(89)90020-5.

McPherson, A. W. et al. (July 2017). "ReMixT: clone-specific genomic structure estimation in cancer". en. In: *Genome Biol.* 18.1, p. 140.

Myers, M. A., G. Satas, and B. J. Raphael (June 2019). "CALDER: Inferring Phylogenetic Trees from Longitudinal Tumor Samples". en. In: *Cell Syst* 8.6, 514–522.e5.

Myers, M. A., B. J. Arnold, V. Bansal, K. M. Mullen, S. Zaccaria, and B. J. Raphael (2023). "HATCHet2: clone- and haplotype-specific copy number inference from bulk tumor sequencing data". In: *bioRxiv*. DOI: 10.1101/2023.07.13.548855. eprint: https://www.biorxiv.org/content/early/2023/07/15/2023.07.13.548855.full.pdf. URL: https://www.biorxiv.org/content/early/2023/07/15/2023.07.13.548855.

Nik-Zainal, S. (2012). “The life history of 21 breast cancers”. In: *Cell* 149, pp. 994–1007. DOI: https://doi.org/10.1016/j.cell.2012.04.023.

Oesper, L., A. Mahmoody, and B. J. Raphael (July 2013). “THetA: inferring intra-tumor heterogeneity from high-throughput DNA sequencing data”. en. In: *Genome Biol.* 14.7, R80.

Pedregosa, F. et al. (2011). “Scikit-learn: Machine Learning in Python”. In: *Journal of Machine Learning Research* 12, pp. 2825–2830.

Popic, V., R. Salari, I. Hajirasouliha, D. Kashef-Haghighi, R. B. West, and S. Batzoglou (May 2015). “Fast and scalable inference of multi-sample cancer lineages”. en. In: *Genome Biol.* 16.1, p. 91.

Rousseeuw, P. J. (Nov. 1987). “Silhouettes: A graphical aid to the interpretation and validation of cluster analysis”. In: *Journal of Computational and Applied Mathematics* 20, pp. 53–65. ISSN: 03770427. DOI: 10.1016/0377-0427(87)90125-7.

Sha, D., Z. Jin, J. Budczies, K. Kluck, A. Stenzinger, and F. A. Sinicrope (Dec. 2020). “Tumor Mutational Burden as a Predictive Biomarker in Solid Tumors”. In: *Cancer Discovery* 10.12, pp. 1808–1825. ISSN: 2159-8274. DOI: 10.1158/2159-8290.CD-20-0522. eprint: https://aacrjournals.org/cancerdiscovery/article-pdf/10/12/1808/1712782/1808.pdf. URL: https://doi.org/10.1158/2159-8290.CD-20-0522.

Shen, R. and V. E. Seshan (June 2016). “FACETS: allele-specific copy number and clonal heterogeneity analysis tool for high-throughput DNA sequencing”. In: *Nucleic Acids Research* 44.16, e131–e131. ISSN: 0305-1048. DOI: 10.1093/nar/gkw520. eprint: https://academic.oup.com/nar/article-pdf/44/16/e131/17437623/gkw520.pdf. URL: https://doi.org/10.1093/nar/gkw520.

Van Loo, P. (2010). “Allele-specific copy number analysis of tumors”. In: *Proceedings of the National Academy of Sciences of the United States of America* 107 (39), pp. 16910–16915. DOI: https://doi.org/10.1073/pnas.1009843107.

Watkins, T. B. K. et al. (Oct. 2023). “Refphase: Multi-sample phasing reveals haplotype-specific copy number heterogeneity”. In: *PLOS Computational Biology* 19.10, pp. 1–31. DOI: 10.1371/journal.pcbi.1011379. URL: https://doi.org/10.1371/journal.pcbi.1011379.

Werner, B. et al. (Feb. 2020). “Measuring single cell divisions in human tissues from multi-region sequencing data”. In: *Nature Communications* 11 (1), p. 1035. ISSN: 2041-1723. DOI: 10.1038/s41467-020-14844-6.

Wintersinger, J. A., S. M. Dobson, E. Kulman, L. D. Stein, J. E. Dick, and Q. Morris (May 2022). “Reconstructing Complex Cancer Evolutionary Histories from Multiple Bulk DNA Samples Using Pairtree”. en. In: *Blood Cancer Discov* 3.3, pp. 208–219.

Zaccaria, S. and B. J. Raphael (2020). “Accurate quantification of copy-number aberrations and whole-genome duplications in multi-sample tumor sequencing data”. In: *Nature Communications* 11 (1), p. 4301. DOI: https://doi.org/10.1038/s41467-020-17967-y.

Zaccaria, S., M. El-Kebir, G. W. Klau, and B. J. Raphael (2018). “Phylogenetic Copy-Number Factorization of Multiple Tumor Samples”. In: *Journal of Computational Biology* 25.7. PMID: 29658782, pp. 689–708. DOI: 10.1089/cmb.2017.0253. eprint: https://doi.org/10.1089/cmb.2017.0253. URL: https://doi.org/10.1089/cmb.2017.0253.

Zeira, R., M. Zehavi, and R. Shamir (Dec. 2017). “A Linear-Time Algorithm for the Copy Number Transformation Problem”. en. In: *J. Comput. Biol.* 24.12, pp. 1179–1194.
