# Supplementary material for: Clone copy number diversity is linked to survival in lung cancer
Source: Nature. 2025 Aug 13;646(8083):190–7. doi: 10.1038/s41586-025-09398-w (PMC12488491; doi:10.1038/s41586-025-09398-w)
Supplement: Supplementary file 2 — Reporting Summary [file 41586_2025_9398_MOESM2_ESM.pdf]

nature portfolio

Corresponding author(s): Nicholas McGranahan

Last updated by author(s): Jul 8, 2025

Reporting Summary

Nature Portfolio wishes to improve the reproducibility of the work that we publish. This form provides structure for consistency and transparency in reporting. For further information on Nature Portfolio policies, see our Editorial Policies and the Editorial Policy Checklist.

Statistics

For all statistical analyses, confirm that the following items are present in the figure legend, table legend, main text, or Methods section.

n/a	Confirmed	
☐	☒	The exact sample size (*n*) for each experimental group/condition, given as a discrete number and unit of measurement
☒	☐	A statement on whether measurements were taken from distinct samples or whether the same sample was measured repeatedly
☐	☒	The statistical test(s) used AND whether they are one- or two-sided<br>*Only common tests should be described solely by name; describe more complex techniques in the Methods section.*
☐	☒	A description of all covariates tested
☐	☒	A description of any assumptions or corrections, such as tests of normality and adjustment for multiple comparisons
☐	☒	A full description of the statistical parameters including central tendency (e.g. means) or other basic estimates (e.g. regression coefficient) AND variation (e.g. standard deviation) or associated estimates of uncertainty (e.g. confidence intervals)
☐	☒	For null hypothesis testing, the test statistic (e.g. *F*, *t*, *r*) with confidence intervals, effect sizes, degrees of freedom and *P* value noted<br>*Give P values as exact values whenever suitable.*
☒	☐	For Bayesian analysis, information on the choice of priors and Markov chain Monte Carlo settings
☒	☐	For hierarchical and complex designs, identification of the appropriate level for tests and full reporting of outcomes
☐	☒	Estimates of effect sizes (e.g. Cohen's *d*, Pearson's *r*), indicating how they were calculated

Our web collection on statistics for biologists contains articles on many of the points above.

Software and code

Policy information about availability of computer code

Data collection	No data was collected in this study. For details, see previously published TRACERx study (https://doi.org/10.1038/s41586-023-05783-5).
Data analysis	All code for running the model and reproducing the figures, as well as the full list of libraries, packages and dependencies can be found at: https://github.com/McGranahanLab/ALPACA-paper (figures reproduction) and https://github.com/McGranahanLab/ALPACA-model (running the model). The end-to-end pipeline is available at https://github.com/McGranahanLab/ALPACA-pipeline.<br><br>HATCHet2<br>CONIPHER(v2.2.0)<br>MEDICC2(v1.1.2)<br>TUSV-ext<br>cloneMap<br>R(v4.3.3)<br>Python(v3.8.19)<br>bradleyterry2 (v1.1_2)<br>survival (v3.6_4)<br>gurobipy (v11.0.1)<br>kneed (v0.8.5)<br>scipy (v1.10.1)

For manuscripts utilizing custom algorithms or software that are central to the research but not yet described in published literature, software must be made available to editors and reviewers. We strongly encourage code deposition in a community repository (e.g. GitHub). See the Nature Portfolio guidelines for submitting code & software for further information.

Data

Policy information about availability of data

All manuscripts must include a data availability statement. This statement should provide the following information, where applicable:

- Accession codes, unique identifiers, or web links for publicly available datasets
- A description of any restrictions on data availability
- For clinical datasets or third party data, please ensure that the statement adheres to our policy

Processed data used in this study has been deposited at Zenodo - a platform maintained by CERN serving for sharing and preserving data for scientific publications. The TRACERx primary and matched primary-metastasis processed data (Zenodo record 7822002) has been deposited at Zenodo https://doi.org/10.5281/zenodo.7822002. Single-cell study from TRACERx PEACE study (Zenodo record 13754279) has been deposited at https://doi.org/10.5281/zenodo.13754279. Processed data used in this publication (Zenodo record 15519765) has been deposited at (https://doi.org/10.5281/zenodo.15519765).

The WES data (from the TRACERx study) used during this study have been deposited at the European Genome–Phenome Archive (EGA), which is hosted by the European Bioinformatics Institute (EBI) and the Centre for Genomic Regulation (CRG) under accession code EGAS00001006494; raw scDNA-seq data used in this study from the patient enrolled in the TRACERx and PEACE studies have been deposited at the European Genome–Phenome Archive (EGA) under accession code EGAD00001015411. Access is controlled by the TRACERx and PEACE data access committees. Details on how to apply for access are available on the ega-archive.org page.

Research involving human participants, their data, or biological material

Policy information about studies with human participants or human data. See also policy information about sex, gender (identity/presentation), and sexual orientation and race, ethnicity and racism.

Reporting on sex and gender	N/A
Reporting on race, ethnicity, or other socially relevant groupings	N/A
Population characteristics	N/A
Recruitment	N/A
Ethics oversight	N/A

Note that full information on the approval of the study protocol must also be provided in the manuscript.

Field-specific reporting

Please select the one below that is the best fit for your research. If you are not sure, read the appropriate sections before making your selection.

☒ Life sciences ☐ Behavioural & social sciences ☐ Ecological, evolutionary & environmental sciences

For a reference copy of the document with all sections, see nature.com/documents/nr-reporting-summary-flat.pdf

Life sciences study design

All studies must disclose on these points even when the disclosure is negative.

Sample size	This study used publicly available TRACERx data, and no additional sample size or power calculations were performed. However, the size of the TRACERx cohort was selected to study the relation between intra-tumour heterogeneity and disease-free survival. For details, see the original TRACERx study https://doi.org/10.1038/s41586-023-05783-5. 395 primary tumours and 126 paired primary-metastatic tumours had all the required ALPACA inputs.
Data exclusions	Tumours without the required input to ALPACA (where only single sample was available or where phylogenetic analysis identified only single clone) were not used in the analysis. This exclusion criterion was pre-established.
Replication	The analysis can be reproduced using the published TRACERx study data (https://doi.org/10.1038/s41586-023-05783-5) and ALPACA repositories deposited on the GitHub platform. Necessary dependencies can be found in these repositories. Attempted replications were successful.
Randomization	Randomization is not relevant as this is an observational study.
Blinding	Randomization is not relevant as this is an observational study.

Reporting for specific materials, systems and methods

We require information from authors about some types of materials, experimental systems and methods used in many studies. Here, indicate whether each material, system or method listed is relevant to your study. If you are not sure if a list item applies to your research, read the appropriate section before selecting a response.

Materials & experimental systems

n/a	Involved in the study
☒	☐ Antibodies
☒	☐ Eukaryotic cell lines
☒	☐ Palaeontology and archaeology
☒	☐ Animals and other organisms
☒	☐ Clinical data
☒	☐ Dual use research of concern
☒	☐ Plants

Methods

n/a	Involved in the study
☒	☐ ChIP-seq
☒	☐ Flow cytometry
☒	☐ MRI-based neuroimaging

Plants

Seed stocks	N/A
Novel plant genotypes	N/A
Authentication	N/A
